# Systems Genetics Analysis of Mouse Chondrocyte Differentiation

**DOI:** 10.1002/jbmr.271

**Published:** 2010-10-14

**Authors:** Jaijam Suwanwela, Charles R Farber, Bau-lin Haung, Buer Song, Calvin Pan, Karen M Lyons, Aldons J Lusis

**Affiliations:** 1 Department of Oral Biology, School of DentistryUCLA, Los Angeles, CA, USA; 2 Department of Prosthodontics, Faculty of Dentistry, Chulalongkorn UniversityBangkok, Thailand; 3 Center for Public Health Genomics, Departments of Medicine and Biochemistry and Molecular Biology, University of VirginiaCharloltesville, VA, USA; 4 Department of Molecular, Cell, and Developmental Biology and Department of Orthopedic Surgery, David Geffen School of MedicineUCLA, Los Angeles, CA, USA; 5 Department of Medicine, David Geffen School of MedicineUCLA, Los Angeles, CA, USA; 6 Department of Human Genetics, David Geffen School of MedicineUCLA, Los Angeles, CA, USA; 7 Department of Microbiology, Immunology, and Molecular Genetics, David Geffen School of MedicineUCLA, Los Angeles, CA, USA; 8 Molecular Biology InstituteUCLA, Los Angeles, CA, USA

**Keywords:** CARTILAGE DEVELOPMENT, SYSTEMS GENETICS, COEXPRESSION NETWORK, MODULE, QUANTITATIVE TRAIT LOCUS, GENE KNOCKDOWN

## Abstract

One of the goals of systems genetics is the reconstruction of gene networks that underlie key processes in development and disease. To identify cartilage gene networks that play an important role in bone development, we used a systems genetics approach that integrated microarray gene expression profiles from cartilage and bone phenotypic data from two sets of recombinant inbred strains. Microarray profiles generated from isolated chondrocytes were used to generate weighted gene coexpression networks. This analysis resulted in the identification of subnetworks (modules) of coexpressed genes that then were examined for relationships with bone geometry and density. One module exhibited significant correlation with femur length (*r* = 0.416), anteroposterior diameter (*r* = 0.418), mediolateral diameter (*r* = 0.576), and bone mineral density (*r* = 0.475). Highly connected genes (*n* = 28) from this and other modules were tested in vitro using prechondrocyte ATDC5 cells and RNA interference. Five of the 28 genes were found to play a role in chondrocyte differentiation. Two of these, *Hspd1* and *Cdkn1a*, were known previously to function in chondrocyte development, whereas the other three, *Bhlhb9*, *Cugbp1*, and *Spcs3,* are novel genes. Our integrative analysis provided a systems-level view of cartilage development and identified genes that may be involved in bone development. © 2011 American Society for Bone and Mineral Research.

## Introduction

Mesenchymal cell condensation is the starting point of skeletal patterning, outlining the shapes of future bones.(1) In endochondral ossification, the main type of bone formation, mesenchymal cells condense and differentiate into chondrocytes to shape the early skeleton. The hyaline cartilage then becomes infiltrated with blood vessels and osteoblasts, forms periosteum, and finally becomes mineralized.(2–4)

The limbs are formed from buds of mesoderm surrounded by ectodermal cells. The apical ectodermal cells regulate the distal outgrowth of the developing limb, and the dorsal ectoderm regulates dorsal-ventral patterning. The mesodermal cells subsequently differentiate into chondrocytes to form the templates of the long bones. Chondrocytes in the growth plates of long bones undergo a coordinated differentiation process. They begin to proliferate, hypertrophy, and undergo terminal differentiation. Chondrocyte differentiation is a multistep process characterized by successive changes in cell morphology and gene expression. This multistep process requires the coordinated expression of many genes, including genes encoding proteins for extracellular matrix (ECM), morphogenesis, proliferation, angiogenesis, and apoptosis.(5,6) Disturbances of these processes can lead to skeletal dysplasias.(6,7)

We now report the application of a systems genetics approach to identify cartilage gene networks and to examine their involvement in bone development. Most of the knowledge regarding the function of genes involved in bone developmental processes has been derived from studies in animal models and cell lines, as well as from the identification of disease genes in skeletal disorders. However, such studies provide insights only into the roles of individual genes or developmental pathways. In contrast, integrative genomics is an approach that links different levels of a biologic system, such as the genome, transcriptome, and phenome, to understand their relationships. It analyzes all genes in the genome simultaneously. This approach organizes gene expression data into a functionally relevant framework in order to explore the physiology of skeletal development from a systems perspective.(8,9)

A central concept of systems biology is that of networks consisting of nodes and edges. In coexpression networks, the nodes represent gene transcripts, and the edges represent correlations between transcripts. The networks in our study were generated by coexpression analysis of mouse rib cartilage cells in 27 different recombinant inbred strains of mice. One highly coregulated subnetwork, or module, was significantly correlated with bone-development parameters among strains. Genes from this module were used to identify the potential regulators of chondrocyte development.(10) Highly connected genes in this and other modules then were studied with respect to expression profiles during differentiation of a chondrocyte cell line, and those exhibiting significant developmental regulation were further examined by siRNA knockdown for effects on chondrocyte differentiation. These studies resulted in the identification of two genes previously shown be important in bone development as well as three novel genes.

## Materials and Methods

### Animals and chondrocyte isolation

The recombinant inbred strains that have been proven to have significant differences in bone traits and that were available from the Jackson Laboratories (Bar Harbor, ME, USA) were used. Those strains were C3H/HeJ, C57BL/6J, DBA/2J, B6Cc3-1/KccJ, BXH2/TyJ, BXH4/TyJ, BXH6/TyJ, BXH7/TyJ, BXH8/TyJ, BXH9/TyJ, BXH14/TyJ, BXH19/TyJ, BXH22/KccJ, BXD1/TyJ, BXD2/TyJ, BXD6/TyJ, BXD 12/TyJ, BXD16/TyJ, BXD 19/TyJ, BXD21/TyJ, BXD 24a/TyJ, BXD27/TyJ BXD 28/TyJ, BXD 32/TyJ, BXD 39/TyJ, BXD 40/TyJ, and BXD 42/TyJ recombinant inbred (RI) strains. All mouse protocols were performed according to the guidelines of the American Association for Accreditation of Laboratory Animal Care (AAALAC). Cartilage from the rib cage of 1- to 2-day-old male mice was dissected to remove bone and any adherent noncartilage tissue for microarray analysis. The cartilage was digested in 0.3% bacterial collagenase (Invitrogen, Carlsbad, CA, USA) for 10 hours, and the cells were collected by centrifugation. RNA was isolated and purified using the Rneasy kit (Qiagen, Valencia, CA, USA). It then was quantified and assessed for purity using a NanoDrop spectrophotometer (Rockland, DE, USA). RNA integrity was verified with a BioAnalyzer 2100 (Agilent, Santa Clara, CA, USA). Given the small amount of RNA we could isolate from the rib cages, we had to pool the RNA from three mice from the same strain. All 27 strains were applied separately to the Illumina arrays.

### Microarray experiments

Illumina Mouse-6 V1 BeadChip mouse whole-genome expression arrays (Illumina, Inc., San Diego, CA, USA) were used in this study. Of the 27 RNA samples from 27 strains, 3 were hybridized twice and were used as technical replicates. A total of 200 ng of DNA-free, quality-checked RNA was amplified using the Ambion Illumina RNA amplification kit with biotin UTP (Enzo, Farmingdale, NY, USA) labeling. The Ambion Illumina RNA amplification kit uses T7 oligo(dT) primers to generate single-stranded cDNA, followed by a second-strand synthesis to generate double-stranded cDNA, which then is column-purified. In vitro transcription was done to synthesize biotin-labeled cRNA using T7 RNA polymerase. The cRNA then was column-purified. The cRNA then was checked for size and yield using the Molecular Probes Quant-iT RiboGreen assay (Invitrogen). A total of 1.5 µg of cRNA was hybridized to each array using standard Illumina protocols with streptavidin-Cy3 (Amersham, Sigma, St. Louis, Mo, USA) being used for detection. Slides were scanned on an Illumina Beadstation and processed using BeadStudio (Illumina, Inc.).

### Data extraction and normalization

The R software (http://cran.r-project.org/), a system for statistical computation and graphics, was used to analyze the data.(11) Data were normalized using Lumi,(12) a Bioconductor (Seattle, WA, USA) package designed to analyze Illumina microarray data that includes data input, quality control, variance stabilization, normalization, and gene annotation. The function “lumiExpresso,” which uses a variance-stabilizing transformation (VST) algorithm, was used to take advantage of the technical replicates available on every Illumina microarray.

### Construction of a weighted gene coexpression network and functional categorization of genes

**Fig. 1:**
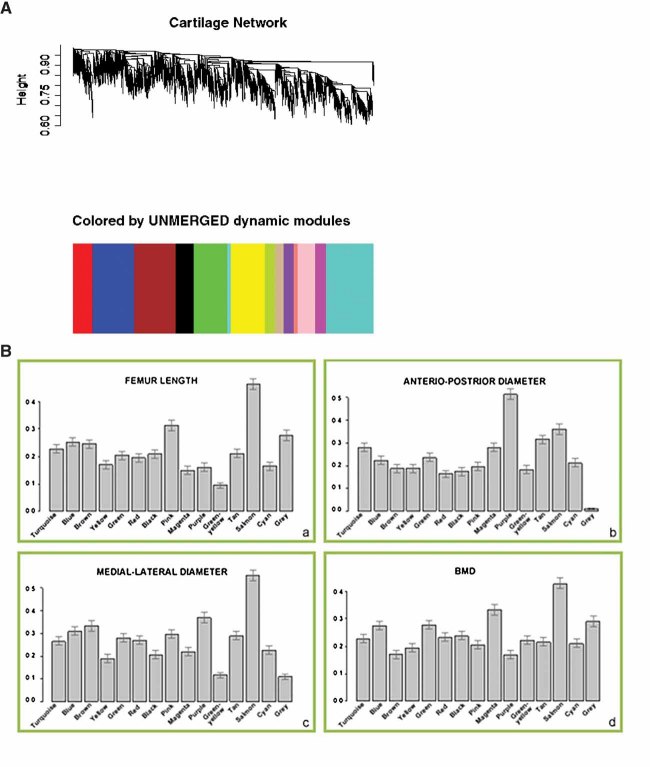
Generation of a cartilage gene coexpression network and associations with bone traits. *(A)* Cluster dendrogram of 3600 genes grouped into distinct modules. The *y*-axis corresponds to the distance determined by the extent of topological overlap (1–TO). Dynamic tree cutting was used to divide modules at significant branch points in the dendrogram (see “Materials and Methods”). The colored bars correspond directly to each module color designation. *(B)* Module significance values for four different bone traits. Module significance was defined as the average of the correlations between each module gene's expression and trait. A module significance of 0.40 is statistically significant at a *p*-value of 0.05. Module significant scores for all modules and femur length (a), anterio-posterior diameter (b), medial-lateral diameter (c), and BMD (d).

Network methods have been applied to identify and characterize various biologic interactions.(13–15) Weighted gene coexpression network analysis (WGCNA) was performed on variably expressed genes in this study, as described previously.(8,16,17) Briefly, the data were filtered to minimize noise owing to biologically irrelevant genes. First, the array detection values were used to select the 9623 genes with statistical evidence of expression in at least 50% of the strains. The 3600 most varying genes then were selected for network construction. A correlation matrix was obtained by calculating the Pearson correlations between all variable probe sets across all strains. In weighted networks, soft thresholding of the Pearson correlation matrix is used for determining the connection strengths between two genes. A soft power adjacency function *a*_*ij*_ = |cor(*x*_*i*_, *x*_*j*_)|^β^, where *a*_*ij*_ represents the resulting adjacency, then was used to construct an adjacency matrix. We chose a power of β = 6 based on the scale-free topology criterion proposed in Zhang and Horvath.(8) This power was chosen such the resulting network exhibited approximate scale-free topology and a high mean number of connections.(12,17–19) For module identification, topologic overlap (TO), a biologically meaningful measure of node similarity, was calculated. A pair of genes was said to have high TO if they were both strongly connected to the same group of genes. Next, the probe sets were clustered hierarchically using dissimilarity, 1 – TO, as the distance measure, and modules were determined using a dynamic tree-cutting algorithm (www.genetics.ucla.edu/labs/horvath/binzhang/DynamicTreeCut). Modules were defined as sets of genes with high “topologic overlap.”(8,21) The algorithm for dynamic tree cut was based on an adaptive process of cluster decomposition and combination, and the process was iterated until the number of clusters became stable. As a result, modules corresponded to branches of the dendogram (Fig. 1*A*). The whole network connectivity (*k*_all_) for each gene was determined by summing the connectivities of that gene with each of the other genes in the network, and intramodular connectivity (*k*_in_) for each gene was determined by summing the connectivities of that gene with all other genes in that module. Genes with the highest *k*_in_ were defined as module hub genes. Networks were depicted graphically using WebQTL (www.genenetwork.org). Group analyses were performed on all generated gene lists using the Database for Annotation, Visualization and Integrated Discovery (DAVID) online analysis program (Version 6, http://david.niaid.nih.gov/david/ ease.htm). Genes from each module in the coexpression network were submitted separately to the DAVID database, which clusters genes according to a series of common gene ontology and pathway categories. The proportion of module genes in each category then was compared with the proportion in the whole genome to compute enrichment scores and corresponding *p* values.(22,23)

### Integrating network analysis, bone geometry, and bone mineral density (BMD)

Adult mice from the same strains that were used for microarray were euthanized at 16 weeks of age. Femurs from five mice of each strain were dissected free of soft tissue, and length, anteroposterior diameter, and mediolateral diameter at the midshaft were measured to the nearest 0.01 mm with digital calipers.(24) BMD scans were performed using a Lunar PIXImus II Densitometer (GE Healthcare, Piscataway, NJ, USA). The averages of trait measurements from five mice from each strain were used. To examine the overall correlations of module genes with each of the phenotypes, we made use of gene significance (GS) measures. The GS was calculated as the absolute value of the correlation between each trait and gene expression values. For example, GS femur length(*i*) = |cor[x(*i*), femur length]|, where *x*(*i*) is the gene expression profile of the *i*th gene. We could assume that the higher the absolute value of GS(*i*), the more biologically significant was the *i*th gene. Module significance (MS) was determined as the average GS measured for all genes in a given module. Therefore, MS provided a measurement for overall correlation between the trait and the module. To assign a *p* value to the MS measure, we used the Fisher transformation as implemented in the *R* function “cor.test.”

### Genotype and module QTL analysis

Genotype data for the BXD and BXH strains were obtained from Gene Network (www.genenetwork.org).(25) The BXD genotype file contained genotypes for 3795 markers (mean spacing of 0.6 cM or 0.7 Mb). This genotype file included all markers, both single-nucleotide polymorphisms (SNPs) and microsatellites, with unique strain distribution patterns (SDPs). Further information can be found at www.genenetwork.org/dbdoc/BXDGeno.html and www.genenetwork.org/ dbdoc/BXHGeno.html. QTL linkage mapping was carried out in a combined data set from BXD and BXH strains with the QTL Reaper software package (qtlreaper.sourceforge.net). A genotype file that was a combination of all the BXD and BXH strains was created. For all alleles that were D or H, we renamed it to N. Essentially, we treated this cross as BXN, so the BXHs and BXDs all were the same cross. One thousand permutations of the strain labels were performed to estimate the genome-wide *p* values.(26) QTL graphics were generated using the multiple mapping tool from GeneNetwork. Interval mapping was used to map genetic loci associated with mRNA abundance.(27,28) One thousand permutations were used to calculate the likelihood ratio statistic (LRS) thresholds to assess genome-wide significance in linkage analysis. Logarithms of odds (LOD) values were obtained by dividing the LRS by 4.6. To identify module quantitative trait loci (module QTLs), expression data from the 41 genes in the salmon module were mapped using the QTL “cluster” function. Module QTLs were defined as loci with a significant enrichment for expression quantitative trait locus (eQTL) of the genes within a predetermined gene module. Empirical *p* values and hypergeometric *p* values were used to determine whether the proportion of module genes that map to the module QTL was significantly higher than that of the 3600 network genes. Empirical *p* values were calculated by using 10,000 permutations of 41 randomly sampled genes from the whole genome.

### Cells and culture conditions

Mouse ATDC5 prechondrocyte cells(29) were maintained in medium consisting of a 1:1 mixture of Dulbecco's modified Eagle's medium and Ham's F-12 medium (DMEM/Ham's F12; Gibco, Carlsbad, CA, USA) containing 5% fetal bovine serum (FBS; Atlanta Biologicals, Atlanta, GA, USA), 10 mg/mL of human transferrin (Roche, San Francisco, CA, USA), and 3 × 10^−8^ M sodium selenite (the maintenance medium; Sigma, St Louis, MO, USA) at 37°C in a humidified atmosphere consisting of 5% CO_2_. These cells were passaged every 4 days.(30–32) Chondrogenesis and cartilage nodule formation were induced only in a postconfluent phase when cells were cultured in the maintenance medium supplemented further with 10 µg/mL of bovine insulin (the differentiation medium; BD Biosciences, San Jose, CA, USA). ATDC5 cells were plated in 6-well plastic plates at an initial cell density of 1 × 10^4^ cells/well. Four days after plating (day 0), chondrogenesis was induced by growth in differentiation medium. Cells were grown for another 28 days, with the medium being replaced every other day. The synthesis of ECM was used to evaluate chondrocyte differentiation. Sulfated glycosaminoglycans were visualized by staining with alcian blue. Cells were washed twice with PBS, fixed with methanol at –20°C for 2 minutes, stained with 0.1% alcian blue 8GX (Sigma) in 0.1 N HCl overnight, and rinsed repeatedly with distilled water. The preparations were evaluated by phase-contrast microscopy.

### RNA isolation and real-time RT-PCR

Total RNA was extracted from ATDC5 cells on days 0, 7, 14, 21, and 28 using the RNeasy Mini Kit (Qiagen) and treated with DNase I. First-strand cDNA was synthesized from total RNA (500 ng) using Superscript II enzyme (Invitrogen) and random hexamer primers. A total of 50 ng of cDNA was used as a template for real-time PCR. Sequences for primers were chosen using the Primer 3 program (http://fokker.wi.mit.edu/ primer3/input.html). The sequences of primers are presented in Supplemental Table 1. Primers were used at 5 µM with 10 µL of SYBR Green Master Mix (Qiagen) in a final volume of 20 µL. SYBR Green PCR amplification and real-time fluorescence detection were performed using the iCycler instrument (Bio-Rad, Hercules, CA, USA) PCR, and cycling conditions were as follows: 95 °C for 15 minutes (denaturation), followed by 45 cycles at 94 °C for 15 seconds, 60 °C for 20 seconds (annealing), and 72 °C for 20 seconds (amplification).

### siRNA construction and transfection

Small interfering RNAs (siRNAs) directed against target genes were designed using Ambion Cenix software (Austin, TX, USA) and analyzed by a BLAST search to ensure gene specificity. Two of siRNAs were used per one target gene. An siRNA with a nonsilencing oligonucleotide sequence, 5'-UUCUCCGAACGUGUCACGUTT-3' and 5'-ACGUGACA CGUUCGGAGAATT-3', showing no known homology to mammalian genes was used as a negative control (Qiagen). The ATDC5 cells were plated in 6-well plastic plates at an initial cell density of 1 × 10^4^ cells/well. One day later, cells were transfected with siRNA (260 nM) using the RNAiFect transfection reagent (Qiagen) according to manufacturer's instructions. The differentiation medium was replaced 1 day after each siRNA transfection. On days 0, 7, 14, 21, and 28 after transfection, mRNA was harvested, treated with DNase I, and subjected to reverse transcription and real-time quantitative PCR.

### In situ hybridization

Nonradioactive images of frozen sections of E14.5 embryos were extracted from the GenePaint.org database (www.genepaint.org).(33) Images were exported to Photoshop, and the tonal range was adjusted using the “Levels” command to extend across the full range.

## Results

### Mouse cartilage coexpression network construction and the relationship to underlying biologic processes

The WGCNA network algorithim was applied to a subset of cartilage gene expression data from 27 male BXH and BXD RI strain mice (see “Materials and Methods”). The 3600 most varying genes were used for network construction. Fourteen gene modules were identified (Fig. 1*A*). A full list of genes by module appears in Supplemental Table 1. Genes in each module shared expression patterns that were more similar to one another than to the expression patterns of genes in other modules. We designated each module by an arbitrary color in order to distinguish between modules. The number of genes included in the modules ranged from 38 (cyan) to 569 (turquoise), and their mean overall connectivity (*k*_all_) ranged from 6.14 (cyan) to 79.30 (turquoise).

We were able to biologically characterize the modules using DAVID, a program that measures overrepresentation of genes with specific GO, KEGG, and BioCarta terms relative to a reference list. We examined the functional significance of all modules by testing for enrichment, for molecular function, and for biologic pathway memebership for genes in each module. For molecular function, 11 of 14 modules, not including yellow, cyan, and tan, had significant enrichment in protein-binding function (*p* < .05). The yellow and cyan modules were enriched for catalytic activity (*p* = 4.6 × 10^−8^ and *p* = 4.8 × 10^−3^, respectively), and the tan module was enriched for peroxidase activity (*p* = 1.5 × 10^−2^). Although the expression patterns in each module were different, many of the modules shared similar GO categorizations, suggesting that some modules may be functionally related. For the biologic pathway analysis, we found that eight modules were significantly enriched. Overall, each module was enriched with distinct gene sets belonging to separate biologic pathways (Table 1).

**Table 1:** Analysis of Enrichment of Chondrocyte Module for Known Pathways Using DAVID

Module	Biologic pathway	Bonferroni corrected *p* value
Purple	Glycolysis/gluconeogenesis	1 × 10^−9^
Salmon	Cyclin and cell cycle regulation	4.2 × 10^−4^
Magenta	MAPK signaling	4.3 × 10^−4^
Green yellow	Proteasome	1.2 × 10^−2^
Brown	Ribosome	1.3 × 10^−2^
Blue	Adherens junction	1.3 × 10^−2^
Turquoise	Focal adhesion	1.5 × 10^−2^
Yellow	Wnt signaling	2.2 × 10^−2^

### Identification of hub genes in cartilage coexpression network

By definition, *hub* genes are genes that have high intramodular connectivity (*k*_in_). Previous studies have shown that the most highly connected genes tend to have critical cellular functions.(10,13–17) We defined the *k*_in_ for each gene based on the sum of adjacencies with all other module genes(10,16,34,35) (see “Materials and Methods” and Supplemental Table 2). Here, we selected the three genes with the highest *k*_in_ in each module as hub genes (Supplemental Table 2). Many modules contained hub genes important in chondrocyte differentiation, such as *SRY-box-containing gene 5* (*Sox5*),(36–38) *lamin A* (*Lmna*),(39) *C1q and tumor necrosis factor related protein 3* (*C1qtnf3*),(40,41) *disabled homologue 2* (*Dab2*), and *tumor protein, translationally controlled 1* (*Tpt1*).

### Salmon module genes have high GS with BMD and femoral geometry traits

**Fig. 2:**
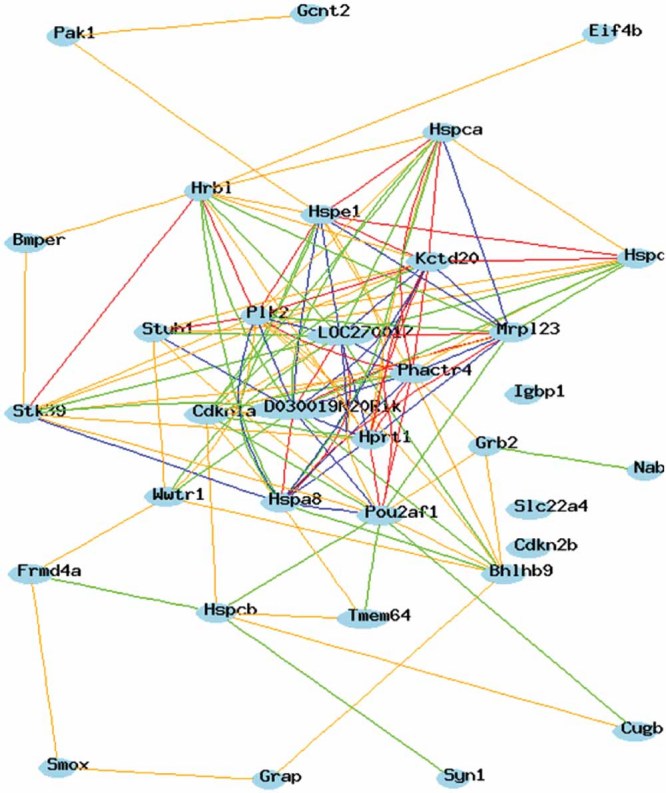
Network view of salmon module genes that have correlation >0.5 and <–0.5. Blue lines represent correlation >0.8. Red lines represent correlation <–0.8. Green lines represent correlation >0.5. Yellow lines represent correlation <–0.5.

The BXH and BXD mice showed a wide range of values for BMD and femoral geometry traits (Supplemental Table 2). Correlation analyses performed from all bone traits showed that only femur length and anteroposterior diameter were significantly correlated (*p* < .0001; Pearson's *r* = 0.53). To determine the physiologic relevance of each module, we measured overall correlations between the traits and the module using the MS scores (see “Materials and Methods”). A module with a high MS value for a particular trait is, on average, composed of genes highly correlated with that trait. Among all modules, the most significant MS scores were observed for the salmon module with all traits, including femur length (MS = 0.42), anteroposterior diameter (MS = 0.42), mediolateral diameter (MS = 0.58), and BMD (MS = 0.48) (*p* < .05), except anteroposterior diameter, with which the purple module had the highest MS score (MS = 0.52) (Fig. 1*B*). The first principal component of the modules (module eigengene) also was used to measure the correlation between the traits and the modules, and this yielded similar, although weaker, correlation (data not shown). Since the salmon module was the most significantly related to bone traits, our follow-up analysis focused on this module. A visualization of the salmon module subnetwork is presented in Fig. 2. General information for all salmon module genes is presented in Supplemental Table 2.

### Genetic control of module gene expression

**Fig. 3:**
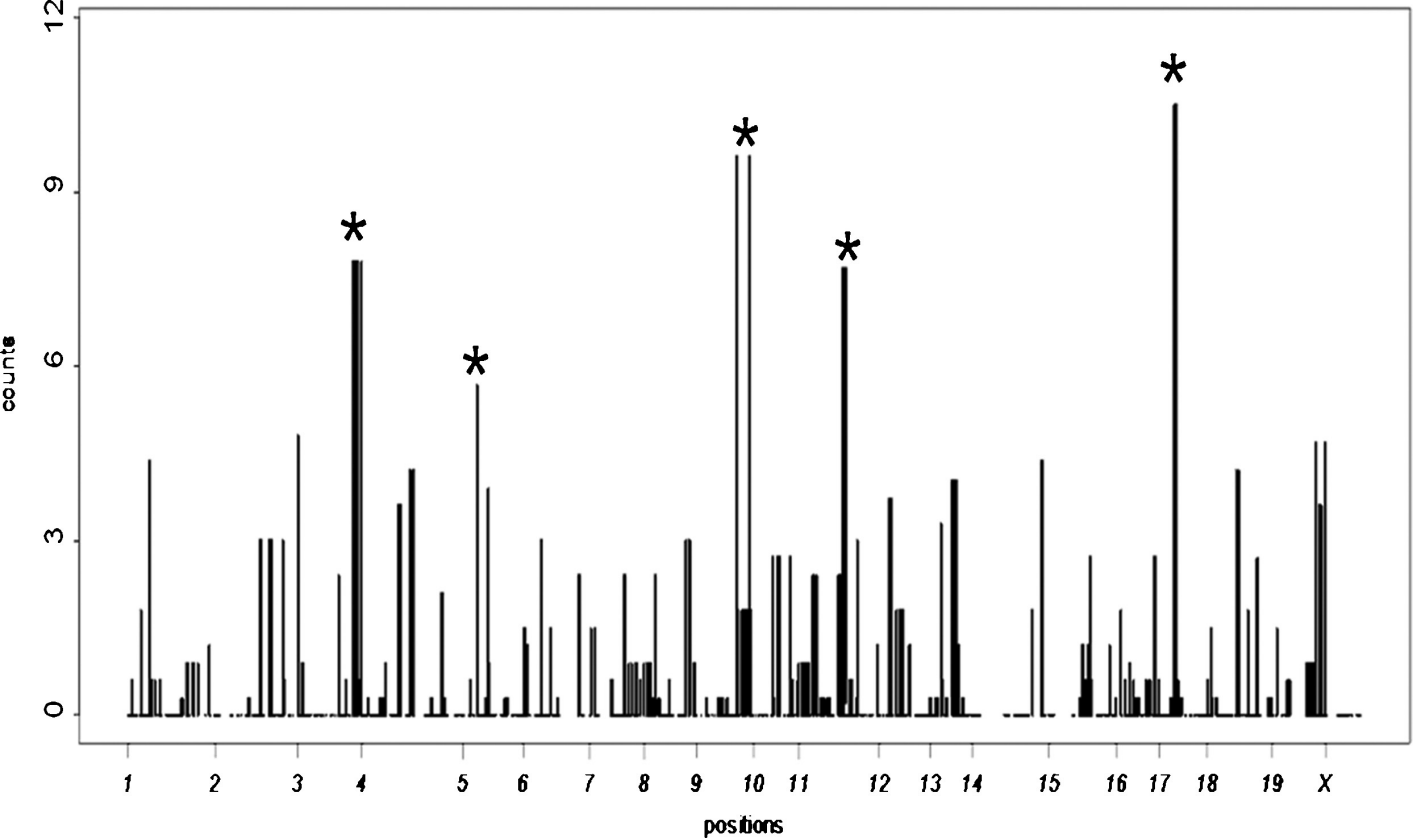
Identification of salmon module QTL. For each marker, the black vertical line represents the number of significant salmon module genes, that is, the number of genes whose single-point LRS at this marker is larger than 13.8 (LOD score >3). The asterisks denote the locations of the module eQTLs reported in Table 2.

We performed QTL mapping to identify genomic regions influencing the expression of modules.(42–44) We used the Web-based eQTL mapping tool WebQTL to visualize putative regulatory loci controlling each transcript. Genomic regions with a significant enrichment for salmon module gene eQTLs were defined as module QTL. The LRS values for the 41 transcripts from the salmon module were plotted against the strength of LRS values. A QTL clustering of the LRS values for the 41 salmon module genes shows patterns of genetic control of salmon module gene expression (Fig. 3*A*). Chromosome 3, 5, 9, 11, and 17 contain clusters that consist of a set of transcripts with significant QTLs (Fig. 3*B*). The details of the peak markers for each chromosome are given in Table 2. These loci are likely to contain genes that regulate the chondrocyte subnetwork related to bone development. Their identification will require fine mapping using congenic strain or other strategies.

**Table 2:** Salmon Genes Module QTL

Module QTL	SNP ID	SNP physical position	Number of transcripts	Mean LRS	Empirical *p* value	Hypergeometric *p* value
Chr 3	rs13477528	chr3:159,474,770-159,475,270	8	23.2	1.04E-02	2.04E-03
Chr 5	rs13478595	chr5:151,148,287-151,148,787	5	26.3	4.10E-03	1.10E-04
Chr 9	rs3705403	chr9:97,259,504-97,260,004	10	20.6	5.30E-03	6.80E-04
Chr 11	rs13481262	chr11:119,215,875-119,216,375	7	27.4	1.30E-04	7.30E-05
Chr 17	rs3706382	chr17:94,530,102-94,530,602	10	30.4	4.30E-05	2.80E-06

### Identification of ATDC5 cell culture stages using chondrogenic markers

**Fig. 4:**
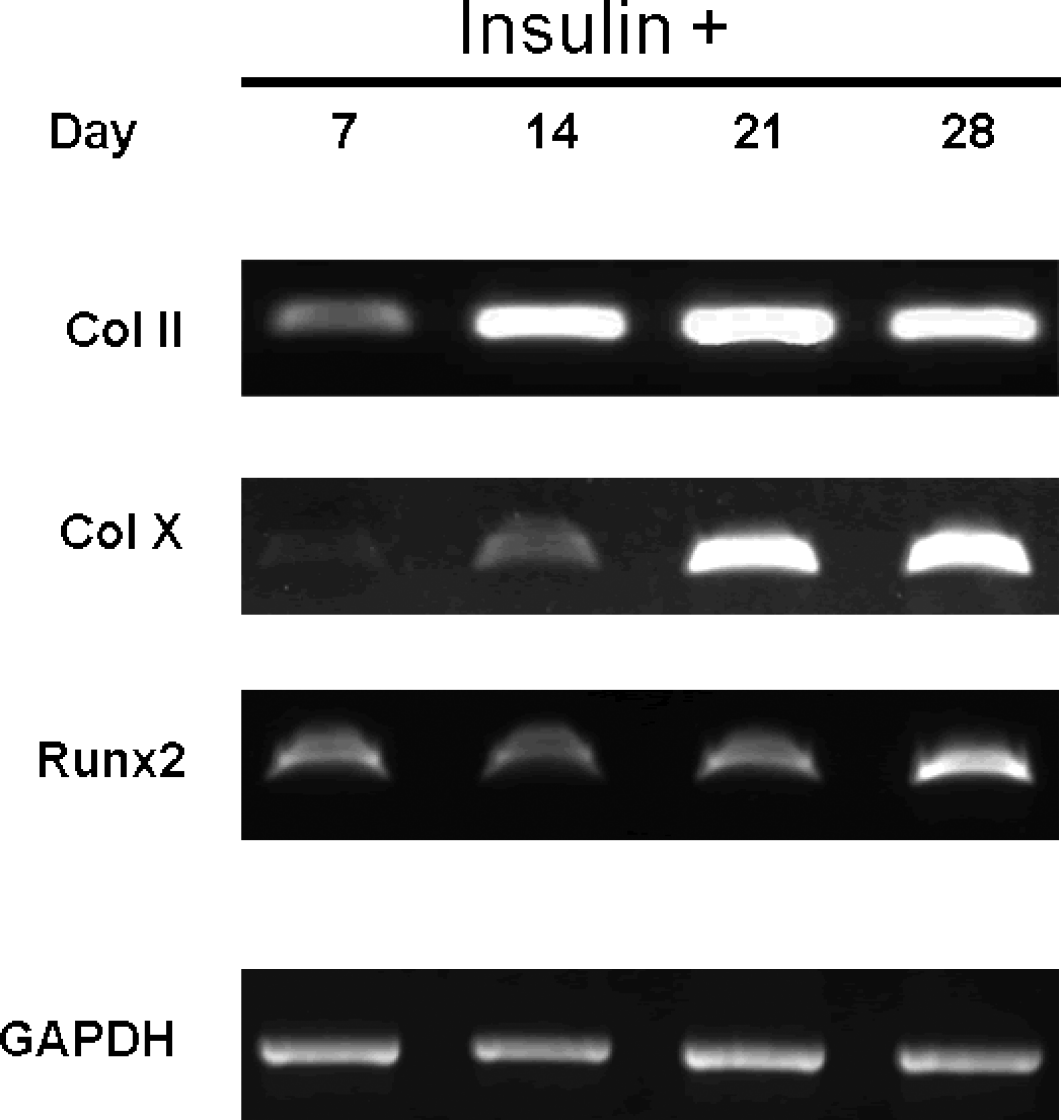
Chondrogenic differentiation of ATDC5 cells. The time-course expression of cartilage genes, *ColII, ColX,* and *Runx2,* are markers for three stages of chondrogenic differentiation in ADTC5 cells.

ATDC5 cells that represent a chondroprogenitor clone were maintained as a growing population of undifferentiated cells and then were induced through an insulin-dependent pathway to differentiate along a pathway corresponding to cartilage development in vivo.(32) As shown in a representative experiment (Fig. 4*A*), the cellular condensations first appeared 5 days after induction, and the formation, growth, and maturation of cartilaginous nodules occurred thereafter on days 11 through 31. The timely pattern of expression of cartilage marker genes characteristic of early and late stages, *collagen type II* (*ColII*), *collagen type X* (*ColX*), and *Runx2*, indicated an orderly progression of chondrogenic differentiation (Fig. 4*B*). *ColII*, which is known to be expressed in proliferating cells, was most highly induced on day 14 and declined gradually from days 21 to 28, whereas *ColX*, which is known to be expressed in hypertrophic cells, was highly induced from days 21 to 28. *Runx2* is known to be expressed in precartilagenous condensations, downregulated in proliferating chondrocytes, and then strongly upregulated in prehypertrophic and hypertrophic chondrocytes. Thus cells on day 7 represented precartilagenous chondrocytes, whereas cells on day 14 are predominantly proliferating chondrocytes. Cells on days 21 and 28 were enriched for prehypertrophic and hypertrophic chondrocytes, respectively. These initial investigations validated our experimental system with regard to the previously established parameters.(32)

### Comparative profile of 28 candidate gene expressions during differentiation of ATDC5

**Fig. 5:**
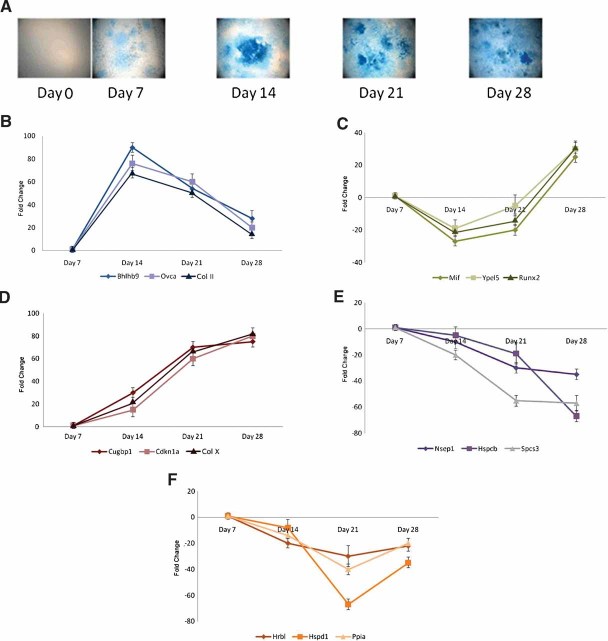
*(A)* ATDC5 cells were induced by insulin and the sulfated glycosaminoglycans (GAGs) were stained with alcian blue. The hypertrophic cells are in dark blue color. Patterns of expression of genes during chondrocyte cell line (ATDC5) differentiation. *(B)* Time-course expression patterns of *Bhlhb9, Ovca,* and *ColII; (C) Mif, Ypel5,* and *Runx2*; *(D) Cugbp1, Cdkn1a,* and *ColX; (E) Nsep1, Hspcb,* and *Spcs3;* and *(F) Hrbl, Hspd1,* and *Ppia.* Total RNA was extracted and reverse transcription was performed. cDNA was analyzed by quantitative real-time PCR using specific primers for candidate genes. Level of expression is normalized to *Gapdh.* Each measure corresponds to the mean ± SE of three independent experiments.

From the results of our network analyses, we selected 28 genes for further investigation. These candidates included the 15 most connected genes in the salmon module and one of the most connected genes from the other 13 modules. The expression patterns of all 28 genes were monitored during the differentiation of ATDC5 cells using quantitative real-time RT-PCR. The aim was to exclude genes that did not exhibit a change in expression during differentiation in order to identify genes whose function can be limited to cell proliferation and differentiation. Twelve of 28 genes demonstrated at least a threefold difference in expression during differentiation. Four distinct expression patterns were found among the genes assayed. The first expression pattern was observed for *basic helix-loop-helix domain–containing class B9* (*Bhlhb9*) and *heparin-binding EGF-like growth factor* (*Ovca*), in which the expression levels increased from day 1 with a peak on day 14 and decreased on days 21 through 28 (Fig. 5*A*). This expression pattern corresponds to *ColII*, which is expressed in proliferating growth plate chondrocytes but not in postmitotic hypertrophic chondrocytes. The second pattern, in which expression increased progressively from days 1 to 28, was observed for *CUG triplet repeat RNA binding protein 1* (*Cugbp1*) and *cyclin-dependent kinase inhibitor 1A* (*Cdkn1a*) (Fig. 5*B*). This expression pattern was seen for *ColX*, a marker for hypertrophic chondrocyte. The third expression pattern was observed for *macrophage migration inhibitory factor* (*Mif*) and *yippee-like 5* (*Ypel5*), in which expression was induced on day 7, gradually reduced on days 14 through 21, and increased again on day 28 (Fig. 5*C*). This expression pattern was similar to the expression pattern of *Runx2*, a gene characteristic of early- and late-stage chondrocytes. The fourth expression pattern, characterized by decreasing expression from the beginning to the end of culture period, was found in *nuclease-sensitive element binding protein 1* (*Nsep1*), *heat shock 90-kDa protein 1, beta* (*Hspcb*), *signal peptidase complex subunit 3 homologue* (*Spcs3*), *ArfGAP with FG repeats 2* (*Hrbl*), *heat shock protein 1* (*Hspd1*), and *peptidylprolyl isomerase A* (*Ppia*). The expression of *Nsep1*, *Hspcb*, and *Spcs3* decreased gradually from days 1 to 28 (Fig. 5*D*), whereas *Hrbl*, *Hspd1*, and *Ppia* decreased from days 1 through 21 with a small increase on day 28 (Fig. 5*E*). The expression patterns of these genes did not follow the patterns of our marker genes, *ColII*, *ColX*, and *Runx2*. However, many of them have functions associated with cellular stress, degradation, apoptosis, etc. *Nsep1* is a member of the EFIA/NSEP1/YB-1 family of DNA-binding proteins. Gene targeting of *Nsep1* induces an early lethal phenotype in embryos owing to either haploinsufficiency of *Nsep1* or formation of a dominant-negative form of the protein.(45) Hence its specific role in chondrogenesis is unknown. *Spcs3* has not been studied in mammalian cells but is essential for protein secretion. Several studies have reported that *Hspd1*, a chaperone protein gene of molecular weight of 60 kDa, is involved in carcinogenesis and apoptosis. It has been reported to be a ligand of toll-like receptor 4 (TLR-4) and induces apoptosis via the TLRs.(46,47) It is also expressed in chondrocytes in response to endoplasmic reticulum (ER) stress.(48) *Ppia*, also as known as *cyclophilin A* (*CyPA*), is a 20-kDa chaperone protein secreted from vascular smooth muscle cells (VSMCs) in response to reactive oxygen species.(49) It is also produced by chondrocytes in response to activation of stress pathways.(50)

We also used previously published microarray data to find the most highly variable gene expression patterns in the in vitro ATDC5 cell model.(51) The 200 most variably expressed genes are shown in Supplemental Table 3 and Supplemental Figs. 1 and 2. We did not detect our candidate genes among those identified in these previous data, but it should be noted that major cartilage-specific genes, such as *Col2a1*, *Col10a1*, *aggrecan*, *Sox9*, and *Runx2* are also not represented. Hence our systems approach identifies a unique set of genes.

### The role of candidate cartilage-related genes in chondrocyte differentiation

**Fig. 6:**
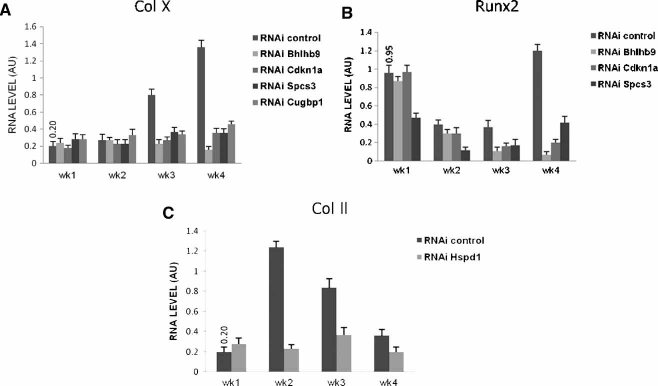
SiRNA knockdown of candidate genes alters differentiation of chondrocyte cell line ATDC5. *(A)* Level of expression for *ColX* after *Bhlhb9*, *Cdkn1a*, *Spcs3,* and *Cugbp1* siRNA transfection. *(B)* Level of expression for *Runx2* after *Spcs3*, *Bhlhb9,* and *Cdkn1a* siRNA transfection. *(C)* Level of expression for *ColII* after *Hspd1* siRNA transfection. Total RNA was extracted and reverse transcription was performed. cDNA was analyzed by quantitative real-time PCR using specific primers for candidate genes or chondrocytes markers. Levels of expression were normalized to *Gapdh.* Each measure corresponds to the mean ± SE of three independent experiments.

To test whether any of the candidate genes we identified are involved in chondrocyte differentiation, the genes that exhibited altered expression throughout the time course of ATDC5 differentiation were knocked down with siRNA. After inhibiting expression, the expression of markers was monitored to determine if reduced expression of these candidate genes affected chondrocyte differentiation. ATDC5 cells treated with nonspecific siRNA had no effect on gene expression during chondrocyte differentiation. In contrast, cells treated with candidate gene–specific siRNAs exhibited reduced expression of the target gene in both siRNAs, on average, of 60% to 70%. This inhibition was specific because *Gapdh* RNA expression was not affected by candidate gene–specific siRNA. Of the 12 candidates, 5 were found to reduce the expression of key chondrocyte differentiation genes. *ColX* mRNA levels were reduced significantly in weeks 3 and 4 when the cells were transfected with *Bhlhb9*, *Cdkn1a*, *Spcs3*, and *Cugbp1* siRNA (Fig. 6*A*). *Runx2* mRNA levels were reduced significantly from weeks 1 to 4 when the cells were transfected with *Spcs3* siRNA and were significantly reduced in weeks 3 and 4 when the cells were transfected with *Bhlhb9* and *Cdkn1a* siRNA (Fig. 6*B*). In contrast, the level of *ColII* was not affected significantly by these genes. *ColII* expression was reduced significantly from weeks 2 to 4 when the cells were transfected with *Hspd1* siRNA (Fig. 6*C*).

### Expression of candidate cartilage-related genes during chondrogenesis

**Fig. 7:**
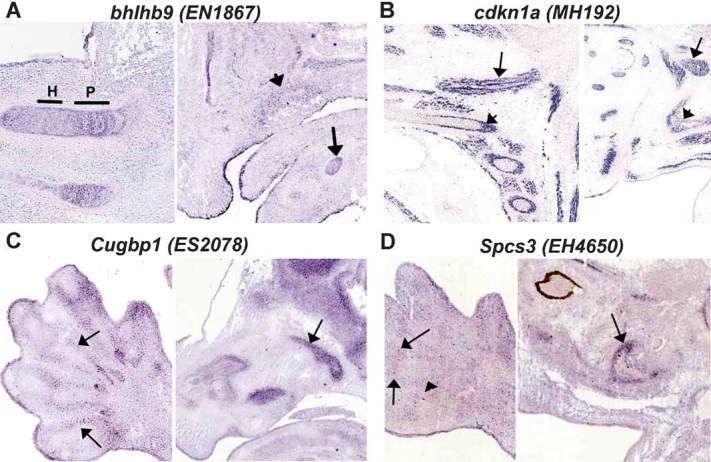
Expression of candidate genes in skeletal tissues. All images are from GenePaint.org (see “Materials and Methods”). The GenePaint dataset is indicated in parentheses. *(A)* Expression of *Bhlhb9* in tibia/fibula (*left panel*) and Meckel's cartilage *(right panel)* at E14.5. *Bhlhb9* is expressed strongly throughout the proliferative zone, the reduced expression in hypertrophic chondrocytes. *Arrow,* Meckel's cartilage. *Arrowhead,* low levels of *Bhlhb9* expression in developing maxillary membrane bone osteoblasts. P, proliferative zone; H, hypertrophic zone. *(B) Cdkn1a* expression at E14.5. *Left panel:* Section through ribs showing low levels of expression in proliferating chondrocytes. *Arrow,* high levels of expression in muscle; *arrowhead,* high levels of expression in perichondrium. *Right panel:* Section through head showing expression in osteoblasts *(arrowhead)*. *Arrow,* expression in muscle. *(C) Left panel: Cugbp1* expression in perichondrium surrounding developing digits *(arrows)*. *Right panel* shows *Cugbp1* expression in developing maxillary bone *(arrow). (D)* Expression of *Spc3*. *Left panel:* Expression is seen at low levels in developing perichondrium within the autopod *(arrowhead)*, and in the area of the developing joints *(arrows)*. *Right panel:* Expression in the periosteal region of developing maxillary bone *(arrow)*.

We examined the patterns of expression of genes shown to affect chondrogenesis in vitro in order to determine whether or not they may have a related function in vivo (Fig. 7). As discussed earlier, expression of *Bhlhb9* follows the same pattern as that of *Col2a1* during ATDC5 cell differentiation, and siRNA against *Bhlhb9* inhibits differentiation. Consistent with an essential function in chondrogenesis, *Bhlhb9* is highly expressed in proliferating growth plate chondrocytes (Fig. 7*A*). The expression levels of *Cdkn1a* and *Cugbp1* increase throughout differentiation, and siRNAs against these genes block terminal maturation, as monitored by *Col10a1* and *Runx2* expression. In situ hybridization of E14.5 embryos reveals that *Cdkn1* is expressed prominently in perichondrium and in osteoblasts but not in proliferating chondrocytes (Fig. 7*B*). Within the fully formed growth plate, *Cdkn1* expression has been shown to be restricted to hypertrophic chondrocytes.(52,53) This pattern of expression is consistent with the block in terminal maturation seen when *Cdkn1a* is inhibited by siRNA. *Cugbp1*, which shows a related pattern of expression during ATDC5 differentiation, shows a similar pattern of expression in vivo (Fig. 7*C*). *Spcs3* levels decline throughout ATDC5 differentiation. Consistent with this time course, siRNA against *Spcs3* leads to impaired ATDC5 function in progenitor cells prior to the onset of chondrocyte differentiation, as monitored by the onset of its effects in week 1.(54–57) Expression of *Spcs3* is seen at highest levels in perichondrium, where progenitor cells reside. Thus the time courses of expression of genes identified in our microarray that show an effect in ATDC5 cells correlate with patterns of expression in vivo.

## Discussion

We used a systems-based approach to identify candidate pathways and genes involved in chondrocyte differentiation. We then tested a subset of these using analyses in a tissue culture model of differentiation. Five genes were shown to influence chondrocyte differentiation significantly, as judged by siRNA knockdown, two of which have been implicated in bone biology in previous studies.

Our strategy was to use a systems genetics experimental design to organize global expression patterns into biologic subnetworks relevant to chondrocyte and bone biology. Thus we isolated chondrocytes from two sets of recombinant inbred strains known to segregate for various bone phenotypes, including size and density, for global expression quantification. The genes then were subjected to weighted gene coexpression network (WGCNA) analysis to identify functional modules. These modules then were related to bone traits among the recombinant inbred strains using correlation. One module (salmon) was highly correlated with several bone traits, in some cases (bone diameter) explaining over 25% of the trait variance.

There are several lines of evidence suggesting that our cartilage coexpression networks are biologically significant. First, WGCNA sorts variable genes and delineates genes into modules enriched for specific molecular functions and biologic pathways. With respect to molecular function, we found that many of the modules share similar GO categorizations. Eleven of 14 modules showed significant enrichment in protein-binding function, even though the expression patterns in each module were different. This suggests that some modules may be functionally related. For biologic pathways, each module was enriched with distinct gene sets belonging to separate biologic pathways. It is noteworthy that many of the resulting enrichment pathways, such as cyclin and cell cycle regulation, as well as the Wnt signaling pathway, are crucial for chondrocyte development. Second, many modules contained hub genes that are important in chondrocyte differentiation. For example, the blue module had a hub gene that is important for cartilage development (*Sox5*). *Sox5* is a critical effector of chondroblast differentiation.(36) It is activated in prechondrocytes and highly expressed in chondroblasts in all developing cartilage elements of the mouse embryo.(37) *Sox5*^−/−^ mutants die in utero with rudimentary and poorly developed cartilage anlagen, whereas *Sox5* heterozygous knockout mice are born with few skeletal abnormalities.(38) One of the hub genes in the green yellow module (*Lmna*) has an effect on bone strength. Heterozygous knockout of *Lmna* increases the frequency of fracture in mice on certain genetic backgrounds.(39) *C1qtnf3*, a hub gene in the yellow module, has been reported to be expressed predominantly in cartilage.(40) Because its product is a secretory protein produced by cartilage tissue, this gene was later named *cartducin*. In situ hybridization analysis showed that *cartducin* transcripts were restricted to the proliferating chondrocytes in the growth plate cartilage. Chondrogenic differentiation stimulated by combined treatment with bone morphogenetic protein 2 and insulin induced cartducin expression along with type II and IX collagen expression in chondrogenic progenitor N1511 cells.(40,41) The hub genes in the magenta (*Dab2*) and turquoise (*Tpt1*) modules both have effects on cell growth. *Dab2* is involved in mesenchymal cell differentiation, whereas *Tpt1* is involved in cell apoptosis. Overall, we found many hub genes that have known biologic functions related to skeletal development. Therefore, other unknown hub genes within the network may have important roles in skeletogenesis. The advantage of our analysis is that these results would never have been uncovered by using traditional microarray analysis methods. Moreover, hub genes from many modules are key players in the biologic processes suggested by DAVID analysis. Finally, most of the modules are conserved when BXH and BXD are analyzed separately (data not shown). These data support the notion that gene coexpression networks can be used to organize gene expression data into a functionally relevant framework to explore skeletal development from a systems perspective.

Because size, shape, and mineral content are all critical components of bone strength, the phenotype under study becomes critical for attempting to understand the biomechanical mechanisms that ultimately define bone morphology and strength. Overall correlation between relevant bone traits and module expression showed that the salmon module is directly related to all bone traits with significant MS scores. This relationship also was evident when examining the list of genes within this module. Three of the 10 most connected genes in the salmon module have known functions related to bone or cartilage: *cyclin-dependent kinase inhibitor 1A* (*Cdkn1a*), *heat shock 60-kDa protein 1* (*Hspd1*), and *Ngfi-A binding protein 1* (*Nab1*). *Cdkn1a* was found to affect the proliferation of chondrocytes. It has been shown to be expressed in postmitotic, hypertrophic chondrocytes but not in proliferating chondrocytes.(58) *Cdkn1a* induces the expression of fibroblast growth factor 2 (FGF-2), a negative regulator of chondrocytic growth, and the inhibitory effect of FGF-2 on chondrocytic proliferation was reduced in part in *Cdkn1a* null limbs.(59) Increased expression of *Cdkn1a* in cartilage has been demonstrated in thanatophoric dysplasia human embryos.(60) The expression of *Hspd1* was found to be increased in secondary hyperparathyroidism, in which the secretion of parathyroid hormone (PTH), the hormone that regulates the rate of growth plate chondrocyte development, is excessive.(61) It is thus reasonable to expect that the highly related hormone, PTHrP, exerts a similar inductive effect on *Hspd1* expression. *Nab1* has a function in bone remodeling. *Nab1* is an endogenous repressor of C-8 sterol isomerase (product of *Erg2*, a gene important for osteoclast survival). Inhibition of *Egr2* increases osteoclast apoptosis. Wild-type *Egr2* or *Egr2* point mutants unable to bind Nab1/2 suppress basal osteoclast apoptosis and rescue osteoclasts from apoptosis induced by *Egr2* inhibition.(62)

The yellow module is enriched with genes involved in Wnt signaling, which is one of the most important pathways for bone development. For example, genes in this module include *connective tissue growth factor* (*Ctgf*), *nephroblastoma overexpressed* (*Nov* or *Ccn3*), and *catenin* (*cadherin-associated protein*) β*1* (*Ctnnb1*). *Ctgf* belongs to a larger CCN gene family and acts downstream of Wnt in cartilage.(63) It is required for endochondral ossification.(64) *Nov*, also a member of the CCN family, is also expressed in growth plate chondrocytes, and is essential for chondrogenesis.(65) *Ctnnb1* or β*-catenin* is as an integral component in the WNT signaling pathway. Through several cytoplasmic relay components, Wnt signal is transduced to β-catenin, which enters the nucleus and forms a complex with transcription factor (TCF) to activate transcription of Wnt target genes. Overall, the yellow module seems to have important function in chondrogenesis and bone development. Therefore, this module would be an interesting module to study in the future. However, the yellow module had low correlation with bone traits. This might be because the Wnt pathway has such significant functions at early stages of skeletal development. We used cartilage from neonatal (P_2_) mice (day 2 mice) for microarray and enrichment analysis, and the correlation with the phenotypes from later-stage (16-week) mice may be low. Clearly, additional phenotypic correlation studies are warranted in the future.

We also used the modulated modularity clustering (MMC) method(66) to analyze our 3600-gene expression data to see how the modules fractionate with other methods (Supplemental Figs. 3 and 4). Some interesting overlap groups of genes are found. Data from MMC Pearson correlation shows that 29 of 41 genes in our salmon module that have high correlation with all bone traits are clustered in one module in MMC. The list of these overlaping 28 genes is in Supplemental Table 4. Data from Spearman correlation shows more scattering of group of genes. However, the genes in the salmon module are still clustered in some modules, as shown in Supplemental Table 4.

As far as the global transcriptional profiles are concerned, only a few investigations have focused on monitoring changes in expression levels of chondrocyte genes in a temporal fashion.(67,68) This study provides a complete determination of expression patterns that can make accurate assessments of candidate gene function during the chondrogenic differentiation pathway. Time-course expression profiles were described for 12 highly regulated genes, including *Bhlhb9*, *Ovca*, *Cugbp1*, *Cdkn1a*, *Mif1*, *Ypel5*, *Ybx1*, *Hspcb*, *Spcs3*, *Hrbl*, *Hspd1*, and *Ppia*. Ten of these had not been associated with chondrogenesis previously. Our knockdown experiments indicated that 5 of the 12 candidate genes regulate the expression of chondrocyte marker genes and that all 5 of these genes are in salmon module. Interestingly, when *Bhlhb9*, the gene that has an expression pattern corresponding to *ColII*, was knocked down, the expression levels of *ColX* and *Runx2* were decreased. This suggests that *Bhlhb9* may play a role in chondrocyte differentiation. When *Cugbp1*, the gene that has an expression pattern similar to *ColX,* was knocked down, the expression level of *ColX* was decreased, raising the possibility that *Cugbp1* is also required for differentiation. The findings that *Cdkn1a* and *Hspd1*, previously known skeletogenesis-associated genes, regulated the expression of chondrogenesis marker genes also validate the approach and analysis. A limitation of this study is that it is a board study to define a network of genes involved in chondrocyte development with some validations and predictions. However, detailed mechansisms of action of the genes identified remain to be determined. We have identified a requirement for *Hspd1*, *Cdkn1a*, *Bhlhb9*, *Cugbp1*, and *Spcs3* in vitro. The in vivo functions of these genes will be investigated in the future studies. Overall, this study suggests that systems-level analyses can provide significant insights not easily reached through conventional approaches.
