# Supplementary material for: Systems Genetics Analysis of Mouse Chondrocyte Differentiation
Source: J Bone Miner Res. 2010 Oct 14;26(4):747–60. doi: 10.1002/jbmr.271 (PMC3179327; doi:10.1002/jbmr.271)
Supplement: Supplementary file 5 [file jbmr0026-0747-SD5.pdf]

Supplementary Table 1 Primer Sequences

Primers	*Forward primers*	*Reverse primers*
RUNX2	5'-GAGGGCACAAGTTCTATCTG-3'	5'-GGCCAAATGTGTCTTCCAGT-3'
TYPE-II COLLAGEN	5'-AGGCGGAGACAGGAACTATC-3'	5'-ACAGAATAGCACCATTGTGTAGG-3'
TYPE-X COLLAGEN	5'-CCAGTAAGAGGAGAACAAGGC-3'	5'-CCTTGGAGTCCAGGACTTCC-3'
GAPDH	5'-CTTGTCATCAACGGGAAGCC-3'	5'-AGACTCCACGACATACTCAGC-3'
YBX1	5'-CCAGGAAGTACCTTCGCAGT-3'	5'-CACCAGGGCCTGTAACATTT-3'
PPIA	5'-GCCAGGGTGGTGACTTTACA-3'	5'-GATGCCAGGACCTGTATGCT-3'
C1QBP	5'-GAAGATGAGGCCGAGAGTGA-3'	5'-CCAGTCCAGGGAATCTGTGT-3'
SPCS3	5'-GCCTCAAGGGAAACAGGAAT-3'	5'-TGGAAATGGCACAGACACAT-3'
GNB1	5'-GAGACTGGCCAGCAGACAAC-3'	5'-GAAGCATCACAAGCACCAGA-3'
ERGIC3	5'-CATGCCTTGTGCCTACTTGA-3'	5'-CGCCATCCTTGTCTAGTCGT-3'
YPEL5	5'-CAAAGGACTGGGTCTGTGCT-3'	5'-CAAATCTGGCCTCTTTCCAA-3'
OVCA	5'-CCCAGAGAGCCTGTGAGAAG-3'	5'-GCAGGAGAAAGGCATTCAAC-3'
SLC39A6	5'-CAAGCCCCCTTCATGAACTA-3'	5'-GTGTCGCTGCATGGTAACTG-3'
CNIH	5'-CACGCGTTCTTCTGTGTCAT-3'	5'-GCCGCTCATCACTGGTCTAC-3'
MIF	5'-CCATGCCTATGTTCATCGTG-3'	5'-GATGTACTGTGCGGGCTTG-3'
NME7	5'-GCCCTTGCGTAGCAATAGAG-3'	5'-GGGTCTCAGGTCGTAAATGC-3'
ATG16L2	5'-CTGTGTGGATGTGGTGA-3'	5'-GACACACACACAGGGATGCT-3'
CAMK2D	5'-CCCTTCTGGGATGAAGATCA-3'	5'-GAGGTCTTTGGCTTCAGGTG-3'
TMEM64	5'-CAGAACAGCGACAAGCTGAG-3'	5'-GGGGACGTCAGTAATCGAAA-3'
SLC22A4	5'-CAAGCTGAGGAGGAGAGGTG-3'	5'-GCGCTGAGCAGAAAGAAGAT-3'
HSPD1	5'-TGTTTGGAGAAGAGGGGTTG-3'	5'-CTTTCAAAAGCATGGCATCA-3'
SMOX	5'-CAAGGACGTGGTTGAGGAAT-3'	5'-TGTTCTGACTCTCGGCATTG-3'
FRMD4A	5'-GGGAGATGAAGTCCCCAGTT-3'	5'-CCAACCTGGCTCACAACATA-3'
PHACTR4	5'-GTCCCCATCTCCTCAAACCT-3'	5'-GTTAGCAGCTCTGGCAACCT-3'
HSPE1	5'-GAAGCTGCCATTCCACTGAT-3'	5'-CAATGGCTGTCAGTTTAGGC-3'

HRBL	5'-CCTTTGGAGCCTACACCAAC-3'	5'-AACACTGCTCATCCCAAAGC-3'
HSPCB	5'-GCGGCAAAGACAAGAAAAAG-3'	5'-GTGATGTCATCCGGGTTTCT-3'
BHLHB9	5'-CCAAGGCAGCCAGAGATATG-3'	5'-GATGGCCACAGCACTAACAA-3'
CUGBP1	5'-GAGTACCATGGAAGCCCTCA-3'	5'-CAGCAGCACCAATACTCTGC-3'
CDKN1A	5'-CTTGTCGCTGTCTTGCACTC-3'	5'-AATCTGTCAGGCTGGTCTGC-3'
POU2AF1	5'-GCAGTTTTTGCTGGAGAAGC-3'	5'-GTAAACAATGGGCCAGTTCC-3'
NAB1	5'-GGCAAGTCAGAAGTGGGAAG-3'	5'-TGCTGGGGAGAGACTATGCT-3'
HSPCA	5'-GCCAGTTTGGTGTTGGTTTT-3'	5'-CTGAGGACTCCCAGGCATAC-3'
