# Supplementary material for: Systems Genetics Analysis of Mouse Chondrocyte Differentiation
Source: J Bone Miner Res. 2010 Oct 14;26(4):747–60. doi: 10.1002/jbmr.271 (PMC3179327; doi:10.1002/jbmr.271)
Supplement: Supplementary file 6 [file jbmr0026-0747-SD6.pdf]

Supplementary Table 1: A complete list of all module genes, their intramodular connectivity ir

Probe Set	Gene	Module	Kin
scl50907.6_	Sfrs3	black	53.571861
scl000646.1	2310061C1!	black	52.662158
scl0011569.	Aebp2	black	51.574699
scl0020383.	Sfrs3	black	50.769539
scl38855.11	3110049J23	black	49.722441
scl0093765.	Ube2n	black	49.672151
scl0067204.	Eif2s2	black	48.179308
scl027058.3	Srp9	black	47.109735
scl013972.9	Gnb1l	black	46.397709
scl020310.4	Cxcl2	black	46.325962
scl48754.10	2410005K2(	black	46.104977
scl25799.5.:	4921520G1:	black	45.591389
scl0014528.	Gch1	black	45.29282
scl33393.12	Nfatc3	black	44.366546
scl0002318.	Ddx24	black	44.196684
scl16319.6.:	Il19	black	43.632601
scl46290.12	1500001L1!	black	43.455828
ri|E030007N	E030007N0	black	43.179676
scl45992.1.:	Slitrk5	black	42.790927
scl29679.10	Trnt1	black	42.722702
scl39780.1.:	C730049P2	black	42.678853
scl36902.11	Scamp2	black	42.397609
scl0022635:	2500001K1:	black	41.871566
scl37697.2.:	1500041O1(	black	41.547731
scl16520.16	Ncl	black	40.212492
ri|C230095C	C230095O0	black	39.515825
ri|9630029C	Nisch	black	38.879725
scl39882.5_	AI316787	black	38.852113
scl019349.2	Rab7	black	37.884384
scl50305.48	Igf2r	black	37.789125
scl020197.3	S100a3	black	37.223127
scl50684.6.:	Mrps18a	black	37.15851
scl25948.44	Gtf2i	black	37.055941
scl54514.7_	Eif1ay	black	36.862291
scl46086.2_	Kctd4	black	36.558457
GI_3809464	LOC385256	black	36.052407
scl0053378.	Sdcbp	black	35.977061
scl26456.10	Nmu	black	35.721172
ri|C230078J	C230078J1:	black	35.297866
scl52512.21	AF233884	black	35.1344
scl0056550.	Ube2d2	black	34.97784
scl078672.2	9530057J2(	black	34.962347
scl012700.3	Cish	black	34.938671
scl098193.1	Wdr42a	black	34.824743
scl097484.1	Cog8	black	34.038503
scl25057.15	Eif2b3	black	33.871829
scl22910.4.:	S100a10	black	33.728143
ri|2600006C	Nudel-pendi	black	33.547359
ri|A430073A	A430073A1	black	33.539022
scl011848.5	Rhoa	black	33.482171

scl066961.2	2310043N1(	black	33.315433
scl46929.4_	Phf5a	black	33.186733
scl51789.1.:	C230075M2	black	33.183881
scl18985.4_	Slc35c1	black	33.17779
scl0013499.	Drr1	black	32.972254
ri|2810002M	Grb10	black	32.613602
scl35231.15	Plcd1	black	32.488159
scl49850.14	BC032203	black	32.449303
scl46821.2.2	2700088M2	black	32.206569
scl013063.3	Cycs	black	32.194299
GI_3809551	LOC385274	black	31.744621
scl013681.1	Eif4a1	black	31.555039
scl0002678.	Magoh	black	31.522932
scl0052040.	Ppp1r10	black	31.512164
scl36356.8_	Ctdspl	black	31.326357
scl078938.5	Fbxo34	black	31.320069
scl39638.4.:	Rpl23	black	31.186259
scl53255.10	D330024H0	black	30.613626
GI_3808425	2410003K15	black	30.609706
scl50683.12	Gtpbp2	black	30.298138
GI_3809389	LOC382163	black	30.277361
GI_3808945	LOC380625	black	30.248884
scl37179.8.:	1700001L23	black	29.951945
scl32119.15	Uqcrc2	black	29.884758
scl41644.4_	Crsp9	black	29.849333
scl0032097(	C430041B1:	black	28.961501
scl013000.1	Csnk2a2	black	28.945128
scl40361.13	Rars	black	28.736162
scl16572.10	Serpine2	black	28.522048
scl31801.4.:	Il11	black	28.48798
scl53420.6.:	Gal	black	28.445131
ri|C330023M	C330023M0	black	28.271935
GI_3808212	AI854251	black	28.13885
scl48709.7_	Ube2l3	black	28.106572
scl22694.6_	Extl2	black	28.086632
scl016391.8	Isgf3g	black	27.572822
ri|E130314B	Capn2	black	27.413302
scl32648.17	Gtf2h1	black	27.039574
ri|9430009A	Zfp288	black	27.028222
scl058520.1	0610007P14	black	26.80893
scl36257.10	Yap1	black	26.733677
ri|8030459N	8030459N02	black	26.587681
scl51376.2_	Synpo	black	26.190918
scl0011603.	Agrn	black	25.481585
scl0078397.	2810449M0	black	25.437606
scl53989.25	Zfp261	black	25.35896
scl24530.11	Ripk2	black	25.357184
scl35326.4.:	Camp	black	25.313169
scl074132.1	Rnf6	black	25.194137
scl47576.1.:	3110045A19	black	24.979974
scl0104303.	Arl1	black	24.838133
scl0011095(	D7Rp2e	black	24.75628
scl48108.3_	Gdnf	black	24.751083
GI_3809099	LOC216443	black	24.5171

ri|A730018N	Camk2d	black	24.498818
scl20297.9_	Ptpns1	black	24.418291
scl0002482.	Slc38a2	black	24.103157
scl43508.21	Map3k1	black	24.01592
scl0077480.	C330002I19	black	23.918872
scl50577.16	Trip10	black	23.709361
ri|7530433C	AU022508	black	23.624993
scl47450.4_	Hoxc6	black	23.592162
scl30753.7.1	Mir16	black	23.434824
scl40848.1_	Clp1	black	23.014372
scl00006.1_	Spec1	black	22.972213
scl068114.7	Mum1	black	22.922245
scl0105278.	Ccrk	black	22.797305
ri|6720464D	Gpc6	black	22.769029
scl36296.9.1	Exosc7	black	22.682083
scl27491.7_	Lrrc5	black	22.408729
ri|E130309L	E130309L16	black	22.086927
scl0002723.	2210021A15	black	22.076581
scl0019883.	Rora	black	21.85751
scl18467.8_	Eif2s2	black	21.661011
ri|6330441H	Ilf2	black	21.608507
scl45065.22	B130016L12	black	21.322669
scl37772.15	Pwp2h	black	21.216525
GI_3807952	LOC208055	black	21.070601
scl0055989.	Nol5	black	20.663797
scl50768.5_	2310014H01	black	20.598252
scl0020678.	Sox5	black	20.440327
scl0018432.	Mybbp1a	black	20.405058
scl0068493.	1110007M04	black	20.23033
scl36156.3_	Edg5	black	20.209046
scl19785.7_	Ogfr	black	20.148965
scl27050.15	Eif3s9	black	20.04549
scl0001581.	Tmc6	black	20.005462
scl0057875.	Angptl4	black	19.76707
scl31534.6.1	Ckap1	black	19.552306
ri|D930007C	Klf7	black	19.085066
scl067105.1	1700034H14	black	19.041942
GI_6677846	Sap18	black	18.967205
GI_6671508	Actb	black	18.950402
scl0019043.	Ppm1b	black	18.881383
scl0001695.	Ppm1b	black	18.7917
scl21371.8.1	Rabggtb	black	18.657331
scl012867.3	Cox7c	black	18.626514
scl0010027	Osbpl9	black	18.429822
ri|E430002C	Nktr	black	17.93166
scl0017134.	Mafg	black	17.91781
GI_3807748	LOC380992	black	17.851229
scl49599.3_	Cdc42ep3	black	17.848521
scl015529.6	Sdc2	black	17.780315
scl24307.19	Catnal1	black	17.65648
scl0022195.	Ube2l3	black	17.627401
ri|D030069G	D030069G1	black	17.528998
scl0077597.	5830406C1	black	17.431978
scl52081.14	Ik	black	17.403902

scl066942.2	Ddx18	black	17.269761
scl49175.14	6820449I09	black	17.235576
scl17564.8_	Mki67ip	black	17.160002
scl33247.15	6430548M0	black	17.125195
scl52738.5_	D19Ertd678	black	17.027122
scl0012321.	Calu	black	16.675986
scl012842.2	Col1a1	black	16.454297
scl069233.1	2810407D0	black	16.403469
scl000949.1	Fn1	black	16.403412
scl0027677(	Eif5a	black	16.394787
scl48768.2_	Socs1	black	16.26401
scl056289.4	Rassf1	black	15.823572
scl51698.9_	Map3k8	black	15.820276
scl16555.3_	Irs1	black	15.795085
scl059013.8	Hnrph1	black	15.78641
scl33231.3_	Gm22	black	15.608985
scl028071.4	Twistnb	black	15.574261
scl34686.15	Slc5a5	black	15.244109
scl37704.3.1	Mrpl54	black	15.196342
scl0022792.	Zrf2	black	15.068852
scl0002898.	Sms	black	15.058052
scl0215160.	Rhbdl7	black	15.003205
scl52395.11	Rnf134	black	14.972229
GI_3807965	LOC381633	black	13.754278
scl0105522.	Ankrd28	black	13.672566
scl0020747.	Spop	black	13.460021
ri|9030013K	9030013K1(	black	13.44707
scl0004168.1_6-S		black	13.155911
scl0001166.	Usp39	black	13.02039
scl48644.15	C330012H0	black	12.875507
GI_6678306	Tff1	black	12.827537
scl38953.16	Prep	black	12.808377
scl0019212.	Pter	black	12.565435
scl066612.1	Ormdl3	black	12.501664
ri|D330021F	Neo1	black	12.411844
scl018950.7	Pnp	black	12.390912
scl17530.23	4732493F0	black	11.380776
ri|1700066F	1700066F0	black	11.280638
scl0003726.	Trim27	black	11.264461
GI_3808186	LOC380617	black	11.174694
scl0004117.	Wdr1	black	11.160787
ri|C230050J	C230050J1	black	10.95197
scl071957.1	2410006F1	black	10.951793
scl0002595.1_82-S		black	10.892073
scl0032813	Slc39a9	black	10.875466
scl31928.9_	Gpr123	black	10.266799
GI_4640229	D330050I23	black	10.210414
scl0100072.	Camta1	black	10.090834
ri|8030469K	Camk2d	black	9.816245
scl49235.18	Tfrc	black	9.7137493
scl0023482	Klhdc4	black	9.6740965
scl0004033.	Arpc1a	black	9.0773359
scl25798.3_	Jtv1	black	8.9753392
scl29824.1.5	Npm3	black	8.9554318

ri|D930027C	Mtssk	black	8.8874481
scl00110842	Etfa	black	8.770327
ri|B230350I(	B230350I06	black	8.417017
GI_3809002	LOC245020	black	8.0327214
scl072515.1	Wrd43	black	7.5977672
scl0170942.	Erdr1	black	6.0002478
GI_3808402	LOC269029	black	5.8881303
scl018186.2	Nrp	black	5.8631596
GI_3807410	LOC381327	black	5.536796
scl34341.1_	D8Ertd587e	blue	40.378303
scl9887.1.1_	2310051F07	blue	37.477006
ri|C130078G	Sox5	blue	37.384837
scl012388.2	Catns	blue	37.093335
scl23675.18	Srrm1	blue	36.639865
ri|2900041I1	Ccnt2	blue	36.288371
ri|4632413C	4632413C1(	blue	35.322708
GI_2848857	C920003I06	blue	34.153615
ri|9830138H	Trps1	blue	33.971941
scl26971.4.5	Rpo1-3	blue	33.831874
scl7952.1.1_	2010309L07	blue	33.688167
scl27279.25	C330023M0	blue	33.397086
scl27030.9_	Foxk1	blue	33.305589
scl5468.1.1_	2900009C1(	blue	33.033035
scl23790.8_	4930429A08	blue	33.000583
scl36131.12	Ankrd25	blue	32.86494
scl0269878.	Egfl4	blue	32.572446
ri|9530073A	9530073A13	blue	32.567989
scl0268377.	BC023928	blue	32.556376
scl0066973.	Mrps18b	blue	32.481311
ri|D030065F	Mark3	blue	32.27091
scl46678.1.1	5830427D02	blue	31.880321
scl25696.1.1	C530036F05	blue	31.576656
scl47510.32	AI317237	blue	31.498567
scl30268.11	2410127E18	blue	31.467066
ri|A130047F	A130047F11	blue	31.356635
ri|5930420P	Msn	blue	31.301009
ri|D430033K	Rps24	blue	31.271616
ri|A430106D	A430106D1	blue	30.921304
scl46838.37	Kif21a	blue	30.875524
scl011538.1	Adnp	blue	30.67386
scl25924.32	Hip1	blue	30.496719
scl0109328.	D6Ertd245e	blue	30.263891
ri|1110034C	1110034O24	blue	30.160425
scl0001376.	AI642036	blue	29.855257
ri|D030025E	Slc11a2	blue	29.424774
ri|D130032J	D130032J17	blue	28.96361
scl073196.2	Rhobtb1	blue	28.959452
ri|D230017C	D230017C0	blue	28.946333
scl41662.1_	9630023C09	blue	28.704052
ri|E430020H	Gs2na-penc	blue	28.640406
scl22882.20	Arnt	blue	28.618667
scl23875.1.1	9230110M14	blue	28.371196
scl52252.1.5	Snrpd1	blue	28.291312
GI_3808684	LOC269941	blue	28.108895

scl29361.1.6	6720403M1	blue	27.502797
scl35019.22	Ikbkb	blue	27.192052
ri|5830465M	Trb	blue	26.651196
scl067534.1	Ttll4	blue	26.584841
scl0056505.	Ruvbl1	blue	26.48193
ri|4932703M	Lrp8	blue	26.44998
scl072190.1	2510009E07	blue	26.205654
scl0029876.	Clic4	blue	26.110113
scl40826.8.1	Mettl2	blue	25.253519
scl093760.1	Arid1a	blue	25.247373
ri|C030034J	C030034J23	blue	25.227868
ri|6030413L	Msi2h	blue	24.872252
scl18038.1.1	9430080K19	blue	24.753293
scl23587.2.1	1700027M0	blue	24.626378
ri|E530020K	E530020K13	blue	24.552763
ri|B230331G	Aebp2	blue	24.513235
scl22971.1.1	2310033F14	blue	24.206273
scl35537.3_	BC023892	blue	24.031186
scl066164.1	1110017C15	blue	24.015124
GI_2089189	9030612K14	blue	23.831163
ri|C630004E	Slc11a2	blue	23.710035
scl19361.2.1	Psmb7	blue	23.41814
GI_2089842	LOC238836	blue	23.008316
scl067684.2	3300001P08	blue	22.702887
scl000586.1	Noc4	blue	22.389105
scl51156.10	Rshl2	blue	22.003395
scl49205.14	Itgb5	blue	21.877364
scl51099.14	Smoc2	blue	21.616525
ri|B130009B	Col6a2	blue	21.614256
scl0071175.	4933421G18	blue	21.422827
GI_3808965	1110031K21	blue	21.375865
scl52554.28	Prkg1	blue	21.218106
ri|4732462B	4732462B05	blue	21.168452
scl0001086.	Bcat1	blue	20.983428
scl070574.7	Cpm	blue	20.982358
scl0067771.	Arpc5	blue	20.966381
scl27921.1.1	Whsc1	blue	20.950766
scl34719.1.4	Cilp2	blue	20.732728
scl0002828.	Ctps	blue	20.585287
scl36214.3_	Gbif	blue	20.507805
scl18732.66	Fbn1	blue	20.472788
ri|C130033E	C130033B1	blue	20.450744
scl39310.22	Exoc7	blue	20.392243
scl069029.1	1500032L24	blue	20.155353
GI_3808293	Fbxo11	blue	20.087453
scl0002627.	Taf12	blue	20.069379
scl0023807.	Arih2	blue	19.998086
scl46641.9_	Rpp14	blue	19.914144
scl38368.11	Wif1	blue	19.801895
scl0012709.	Ckb	blue	19.676356
GI_3807570	LOC218814	blue	19.534872
scl0024568	Rbbp7	blue	19.472071
scl49344.11	Yeats2	blue	19.338298
scl1412.1.1_	2310061A22	blue	19.195212

scl26028.9.1	Eif2b1	blue	19.16444
scl000176.1	Psma1	blue	19.144419
scl0004039.	D5Ertd689e	blue	19.03572
scl37470.5.1	Yeats4	blue	18.873967
ri|5530400P	5530400P07	blue	18.583546
scl066184.1	1110033J19	blue	18.541474
scl27388.28	Ube3b	blue	18.495074
scl0109037.	6230415M2	blue	18.232026
scl013684.8	Eif4e	blue	18.226873
scl19222.27	Fap	blue	18.19896
ri|C530015M	C530015M0	blue	18.198894
scl0067896.	2610001E17	blue	18.119057
ri|4832404P	4832404P21	blue	18.048145
scl32561.2_	A330103N2	blue	17.982666
ri|D230050M	Pum1	blue	17.957641
scl21044.5.1	Angptl2	blue	17.952889
scl074440.1	4933407C03	blue	17.941293
scl51993.3_	Eif1a	blue	17.901552
scl0014228.	Fkbp4	blue	17.849879
scl0326618.	Tpm4	blue	17.746633
scl31984.10	Prss11	blue	17.735123
scl36644.3_	Prss35	blue	17.728921
ri|E030038D	E030038D2	blue	17.688173
scl022121.1	Rpl13a	blue	17.60591
scl48737.1.5	4921513D23	blue	17.590239
scl0002511.	Myh9	blue	17.438534
scl22839.1.3	4930564D15	blue	17.374833
scl35530.18	Mod1	blue	17.248673
scl25791.15	1300006M1	blue	17.186722
scl067270.3	D10Ertd322	blue	17.115397
scl0021348	BC036718	blue	17.080175
scl0017357.	Mlp	blue	17.005684
GI_8570169	AI427138	blue	16.955198
scl067223.1	2810430M0	blue	16.931607
ri|9130227N	9130227N12	blue	16.925148
scl51577.15	Brunol4	blue	16.897359
scl0022535	2610024E20	blue	16.75234
scl52790.5_	1810055G02	blue	16.725472
ri|A230090H	A230090H1	blue	16.705447
scl26190.16	Coro1c	blue	16.679457
ri|6530402L	Itgb4bp	blue	16.665969
scl27909.18	Add1	blue	16.631755
scl069902.2	2610012O22	blue	16.592568
scl0210356.	E030049G2	blue	16.500493
GI_3808980	Mll	blue	16.45279
scl37453.4_	1810038L18	blue	16.368384
scl28812.19	Hk2	blue	16.366731
scl26446.8.1	2610024G14	blue	16.311072
ri|2310010I1	2310010I15	blue	16.27707
scl0097064.	2310058J06	blue	16.223028
ri|4921535H	Ywhag	blue	16.215832
scl17336.3.1	2810025M1	blue	16.213217
scl17296.11	Vamp4	blue	16.18977
scl34795.4.1	Sap30	blue	15.823347

scl0246257.	Ovca2	blue	15.754097
scl016362.1	Irf1	blue	15.656757
scl021814.1	Tgfbr3	blue	15.634368
scl30296.3.2	2310016C08	blue	15.496305
scl21487.17	E130014J05	blue	15.397782
scl016149.9	Ii	blue	15.39014
GI_3134242	Pramel4	blue	15.276445
GI_3808933	LOC382020	blue	15.255415
scl34177.5_	2310022B05	blue	15.247548
scl51415.5.1	Ppic	blue	15.191663
ri|2810405J2	2810405J23	blue	15.186911
ri|6530401D	6530401D06	blue	15.169216
scl43527.1.7	9930031P18	blue	15.159799
scl0019216.	Ptger1	blue	15.095837
scl28224.2_	A730017D0	blue	15.035203
scl23010.9_	2610029K21	blue	15.021699
scl36337.4.1	1500010M1	blue	14.974586
scl0121022.	Mrps6	blue	14.955146
scl0003727.	Hnrpk	blue	14.938289
scl30561.16	Dhx32	blue	14.909523
scl0027999.	D6Wsu176e	blue	14.895136
scl28438.14	Slc2a3	blue	14.869474
scl015000.6	H2-DMb2	blue	14.84843
scl26719.6_	Spon2	blue	14.790668
scl0070769.	Nolc1	blue	14.681065
scl073162.1	3110030K17	blue	14.652141
scl0384009.	Glipr2	blue	14.494357
scl0319360.	C630022N0	blue	14.457981
scl31573.3_	Mrps12	blue	14.447356
scl39574.3.1	Krt1-5	blue	14.331133
scl39322.7.1	Galk1	blue	14.305718
scl26994.6.1	Nptx2	blue	14.277965
scl39850.23	Myo1d	blue	14.187499
scl33660.5_	Hmox1	blue	14.165074
scl26778.4_	Xrcc2	blue	14.137763
scl021858.1	Timp2	blue	14.105853
scl0021812.	Tgfbr1	blue	14.087346
scl0069640.	2310040C09	blue	14.048902
scl51825.4_	2310057H16	blue	14.041646
scl0019699.	Reln	blue	13.926643
scl42936.6.9	Ahsa1	blue	13.924998
scl36508.16	Rnu3ip2	blue	13.913597
scl28596.5_	Rybp	blue	13.873199
scl42219.1_	0610007P14	blue	13.731213
scl53380.11	Fads1	blue	13.724826
scl0016998.	Ltbp3	blue	13.712355
scl0010517	Arrdc3	blue	13.686631
scl18572.5_	Rbbp9	blue	13.548151
ri|2810406C	Csnk2a2	blue	13.516962
GI_3808970	LOC382061	blue	13.374244
scl071916.1	2310069P03	blue	13.35398
scl059053.6	Brp16	blue	13.214273
scl019383.1	Raly	blue	13.162464
scl45988.2_	5033413D16	blue	13.159138

scl30972.4_	P2ry2	blue	13.130166
scl0319679.	Tnfrsf22	blue	13.104832
scl48576.14	D16Bwg154	blue	13.092308
scl0016663.	Krt1-13	blue	13.075651
scl33292.1.8	1110019O1(	blue	13.008783
scl18265.5_	Tmepai	blue	12.996628
scl0001421.	Irf1	blue	12.953391
scl51558.7_	Stard4	blue	12.948256
scl019944.1	Rpl29	blue	12.881914
scl29713.3_	Arl6ip5	blue	12.867465
scl19937.12	Pigt	blue	12.840007
scl013179.1	Dcn	blue	12.814503
scl26199.2.1	2900026A02	blue	12.778225
scl0070930.	Nol8	blue	12.761063
scl36481.1.5	Amigo3	blue	12.700749
scl28403.9.1	Ltbr	blue	12.697484
scl41320.8.1	2400006N03	blue	12.676638
scl28676.3_	Trh	blue	12.668106
scl0056505.	Ruvbl1	blue	12.630453
scl076073.9	0610009F02	blue	12.544889
scl45299.10	Slc25a30	blue	12.497792
scl068552.1	1110003E01	blue	12.459014
scl42993.1.2	2410016O0(	blue	12.451413
scl0020442.	Siat4a	blue	12.359566
scl25647.11	Mmp16	blue	12.319389
scl0014074(	Sec63	blue	12.25735
scl36315.5_	Ccbp2	blue	12.153117
scl16321.6.1	Il24	blue	12.140442
scl0023222;	D6Ertd349e	blue	12.032
scl0021859.	Timp3	blue	12.028754
GI_3807995	LOC383153	blue	12.025602
GI_3808330	LOC195357	blue	12.02384
scl0022350.	Vil2	blue	11.981638
scl0331006.	C730026J1(	blue	11.842645
scl31909.4.1	1190003J15	blue	11.818807
scl33701.2.1	Mrpl34	blue	11.789614
GI_3808311	LOC386463	blue	11.75752
scl18503.5_	Trib3	blue	11.736808
scl36891.2_	Cyp11a1	blue	11.701633
scl16639.44	Fn1	blue	11.684255
scl28293.6.1	Hebp1	blue	11.652477
scl00072.1_	Rab6	blue	11.594106
scl52860.17	Syvn1	blue	11.590818
scl6267.1.1_	6330414G02	blue	11.561346
scl068092.4	Ncbp2	blue	11.470133
scl0002547.1_9-S		blue	11.426704
scl23476.5_	Vamp3	blue	11.372423
scl015510.1	Hspd1	blue	11.36522
ri|2310075E	1110064N1(	blue	11.311696
scl47043.17	Bop1	blue	11.252101
scl0223435.	Trio	blue	11.245979
scl069010.2	Anapc13	blue	11.203654
scl0001008.	Il24	blue	11.169106
scl16861.2.1	9530018I07	blue	11.132559

scl47766.12	Pscd4	blue	11.130019
scl21501.10	4933405A16	blue	11.094337
scl31632.4.1	Cnfn	blue	11.068101
scl31568.26	Actn4	blue	11.023633
scl34240.1.5	1110003O08	blue	11.007206
ri|1810037J0	1810037J08	blue	10.997578
scl0002776.	Eif3s2	blue	10.892779
scl073192.7	Xpot	blue	10.841574
scl35378.12	Mapkapk3	blue	10.830277
scl16594.5_	D1Bwg1363	blue	10.829612
scl35215.8_	Axud1	blue	10.797342
scl0017082.	Il1rl1	blue	10.703592
scl0011564.	Adsl	blue	10.683337
scl014651.8	Hagh	blue	10.531683
scl50377.4.1	3300005D01	blue	10.510376
scl47300.10	Rnf19	blue	10.480581
scl29705.1.6	Mitf	blue	10.479959
GI_3808974	LOC382063	blue	10.430338
scl098970.1	Fibcd1	blue	10.427085
scl0056365.	Clcnkb	blue	10.403315
ri|4831437C	4831437C03	blue	10.366271
scl16258.13	Rnpep	blue	10.358511
scl0014073.	Faah	blue	10.341589
scl068877.8	Maf1	blue	10.237814
GI_1339931	0610007P06	blue	10.225732
scl0001446.	Gosr2	blue	10.225425
scl41823.30	Egfr	blue	10.17708
scl40203.12	Sparc	blue	10.145674
scl0067878.	1600019D15	blue	10.122289
ri|6720463L	6720463L11	blue	10.11657
ri|A730086L	A730086L23	blue	10.08446
scl0022687.	Zfp259	blue	10.081009
scl21615.11	Slc30a7	blue	10.064653
scl0002839.	Srm	blue	10.031124
scl20918.1.1	5230400M03	blue	9.9689698
scl099683.1	Sec24b	blue	9.9580562
scl24818.5.9	Zfp46	blue	9.9539428
scl42798.1.1	1600002O04	blue	9.9407103
scl43560.31	4930420O1	blue	9.9401564
scl030932.2	Zfp330	blue	9.9387258
scl068178.1	4933421H10	blue	9.8658197
scl0001334.	Aoc2	blue	9.840357
scl39808.15	Aatf	blue	9.8379908
ri|6330576B	Faah	blue	9.8375226
ri|A430058L	Arhgef1	blue	9.8193526
ri|9530096I0	Il6ra	blue	9.8022214
scl48583.3_	Lrrc15	blue	9.7952005
scl093692.3	Glrx1	blue	9.7822612
GI_3807629	LOC213480	blue	9.7815549
scl29846.4_	Gcs1	blue	9.7809925
GI_3809346	Dhrsx	blue	9.7642916
GI_3808787	LOC381947	blue	9.7477068
ri|E430027N	E430027N0	blue	9.7466246
scl25095.5.1	Map17	blue	9.7185167

scl40760.4_	Sox9	blue	9.6879982
GI_3808118	LOC381681	blue	9.687427
scl52683.7_	Gcnt1	blue	9.5550052
scl44789.4.1	Omd	blue	9.5483418
scl28441.10	Apobec1	blue	9.5382879
scl0003917.	Nap1l1	blue	9.5328098
scl33402.1_	Thap11	blue	9.5202193
scl30887.1.1	Prkcdbp	blue	9.4912438
scl019822.7	Rnf4	blue	9.4745097
scl52942.12	Nolc1	blue	9.4474767
scl0032661	Tpm4	blue	9.4367288
scl0002855.1_1056-S		blue	9.3995057
scl0016195.	Il6st	blue	9.3876435
scl19851.8.1	Dok5	blue	9.3559471
scl070189.1	2010015P12	blue	9.3275118
scl45046.5.1	A530046M1	blue	9.311245
scl3687.1.1_	Bdkrb1	blue	9.2878107
scl46131.3_	Egr3	blue	9.2307569
scl38713.11	Bsg	blue	9.2051024
scl51556.7_	D0H4S114	blue	9.1479091
scl22486.4_	Bcl10	blue	9.1474793
scl40022.6.1	Cd68	blue	9.1377418
scl27412.8.1	Tpst2	blue	9.1329326
ri|9630009A	9630009A08	blue	9.1194232
scl014289.2	Fpr-rs2	blue	9.118143
ri|2700031B	2700031B12	blue	9.0948552
scl45769.2.2	1810004F21	blue	9.0946632
scl0079199.	Birc1cl	blue	9.0865877
scl0056392.	Shoc2	blue	9.0817077
ri|E430003D	E430003D0	blue	9.0668961
scl0001776.	Mapk1	blue	9.0425658
GI_3809360	LOC382157	blue	9.0295451
scl0075909.	4930579A11	blue	9.0289342
scl54485.6_	Piga	blue	8.9604891
scl0002293.	Sfrs5	blue	8.8724351
scl0004104.	Ttc11	blue	8.8539515
scl0026852	Greb1	blue	8.8422625
scl44251.7.1	Stard3nl	blue	8.8091119
ri|B930097L	B930097L24	blue	8.788409
scl0105239.	Rnf44	blue	8.6706143
scl43911.4.1	Cxcl14	blue	8.6470624
ri|4732450E	Smcx	blue	8.6459553
GI_3807633	LOC219145	blue	8.6426039
ri|D830019K	D830019K0	blue	8.6338403
scl28780.8_	Cyp26b1	blue	8.6258554
scl075608.7	2010012F05	blue	8.6242422
scl0073338.	1700041B20	blue	8.6231034
scl081630.1	Zfp297	blue	8.6123158
scl25834.12	Snx8	blue	8.5935631
scl27883.20	Evc2	blue	8.5820484
scl0071949.	Lass5	blue	8.5770472
scl33993.13	Plat	blue	8.536389
ri|5730490E	Hmg20a	blue	8.5321527
scl20038.8.1	Spag4	blue	8.4383645

GI_3809346	LOC385086	blue	8.3518366
scl19777.13	Arfgap1	blue	8.3468511
scl52642.29	Tjp2	blue	8.3055347
scl47072.5_	Ly6a	blue	8.2997594
GI_3807332	LOC381292	blue	8.2667561
GI_3809090	LOC234374	blue	8.2107034
scl49864.8_	Srf	blue	8.1584407
GI_2447592	Iap	blue	8.1477452
scl0014980.	H2-L	blue	8.1427057
scl0330631.	9530064J02	blue	8.0868695
scl37802.13	Hrmt1l1	blue	8.0457175
scl40078.22	AU040829	blue	8.0422365
ri|5330435L	5330435L01	blue	8.0375991
scl0022499	Dlgap1	blue	8.0065531
GI_3809025	LOC382128	blue	7.9604315
scl42784.1_	6430411K18	blue	7.9596256
scl000196.1	Actn4	blue	7.897244
ri|A930039J	Mapk6	blue	7.8907069
GI_3809342	LOC385068	blue	7.8496875
scl34205.11	3010027A04	blue	7.8300512
scl18658.11	2600009E05	blue	7.8159934
scl000403.1	Slc39a14	blue	7.7844096
scl30437.5_	Ccnd1	blue	7.7790851
scl33953.2_	End2	blue	7.7765845
scl34758.8_	Sc4mol	blue	7.728923
18S_rRNA_X00686_849		blue	7.7238503
scl0107375.	AW491445	blue	7.7116594
scl0013002.	Dnajc5	blue	7.6932005
scl0012443.	Ccnd1	blue	7.6916263
scl40886.4.1	Aoc2	blue	7.6912815
scl0054613.	Siat10	blue	7.6745487
scl0023354	2210018M1	blue	7.6607053
scl0020567.	Slp	blue	7.6102907
scl17460.3_	Fmod	blue	7.5634906
scl30457.1.2	Tssc8	blue	7.5517342
ri|B930008G	B930008G0	blue	7.5436102
ri|A130028H	A130028H1	blue	7.5158143
ri|1200006G	1200006G1	blue	7.4554867
scl0029875.	Iqgap1	blue	7.4455694
scl18448.2.1	Gdf5	blue	7.4122095
ri|E330016A	E330016A0	blue	7.393537
scl012814.4	Col11a1	blue	7.3894625
GI_3807993	LOC224137	blue	7.3868375
scl20002.16	Lbp	blue	7.3727011
scl24711.3.1	Nppb	blue	7.3285357
scl42375.5_	Sav1	blue	7.2169128
scl0014751.	Gpi1	blue	7.1818723
scl44884.17	Riok1	blue	7.1502214
scl46642.13	Abhd6	blue	7.1432765
scl38516.1.1	AI426953	blue	6.9243891
scl51913.28	Slc12a2	blue	6.9117926
scl23694.2.1	Cnksr1	blue	6.887596
scl0002791.1_134-S		blue	6.8668593
scl0105841.	E030003N1	blue	6.8361344

scl020716.5	Serpina3n	blue	6.731199
scl0107449.	Unc5b	blue	6.679602
scl0015598.	Iap	blue	6.6580507
scl41559.17	P4ha2	blue	6.6351439
scl0244668.	Sipa1l2	blue	6.6278024
GI_3154295	Hist1h2ac	blue	6.6254598
scl0072333.	2410003B16	blue	6.5519187
scl40154.14	B430214A04	blue	6.521779
scl23222.4_	Ccrn4l	blue	6.4931323
scl27911.17	Sh3bp2	blue	6.4158785
scl020463.2	Cox7a2l	blue	6.3981659
ri|A130051J	A130051J06	blue	6.3323576
scl29486.14	D6Wsu163e	blue	6.2741946
scl24261.12	Alad	blue	6.2704523
scl53701.1.3	Mageh1	blue	6.2356299
scl0076022.	5830417I10	blue	6.2313965
scl42796.25	Eml1	blue	6.1941224
scl15766.4.1	1110060M2	blue	6.1922007
18S_rRNA_X00686_301		blue	6.1777302
scl33012.5.1	Dmwd	blue	6.1689442
scl0011652.	Akt2	blue	6.1266876
scl32110.5.1	0610012D09	blue	6.1086698
scl0239528.	Eif2c2	blue	6.1062837
scl14261.1.1	Slc30a1	blue	6.09055
scl40009.3.1	Eif5a	blue	6.074674
GI_3808755	LOC330668	blue	6.053312
scl20407.30	Mapkbp1	blue	6.0531463
scl34717.16	BC031407	blue	6.0051952
scl020818.5	Srprb	blue	5.9964681
scl00010.1_	Emp3	blue	5.9468408
scl0010431(	Csnk1d	blue	5.8945608
scl21260.7_	Arl8	blue	5.8551522
scl28865.3.1	Vamp8	blue	5.847705
scl49651.7_	Tgif	blue	5.7585473
scl0021013!	Zfp180	blue	5.6656074
scl0020256.	Scgf	blue	5.6084695
scl056772.1	AI839562	blue	5.5096096
ri|C130064E	C130064E2	blue	5.4507061
scl0224807.	BC026370	blue	5.4154176
scl012293.4	Cacna2d1	blue	5.4125042
scl20807.21	Dncic2	blue	5.3147869
GI_3808993	LOC384943	blue	5.2480521
scl33922.11	Gtf2e2	blue	5.0500247
scl0226359.	BC040774	blue	4.9870058
18S_rRNA_	Pigt	blue	4.9077241
scl078134.3	Gpr23	blue	4.9021559
scl075765.1	4833424O1	blue	4.8665216
scl29514.8.2	Mlf2	blue	4.8204804
scl33560.21	Man2b1	blue	4.7713094
scl0032016!	B230378H1	blue	4.6954238
scl0031993!	Tens1	blue	4.490368
ri|A130009M	A130009M0	blue	4.4896208
scl0030791.	Slc39a1	blue	4.3188771
scl0020829	9030612M1	blue	4.2767431

scl0067102.	D16Ertd472	blue	4.2371533
scl0016423.	Cd47	blue	4.2191609
ri|6030458P	6030458P0(	blue	4.187337
scl47358.1_	Basp1	blue	4.1011556
scl0051788.	H2afz	blue	4.0465493
scl0211623.	MGC41689	blue	3.9939465
ri|D630041L	D630041L1:	blue	3.9526801
scl066383.5	2310020H2(	blue	3.9020601
scl21737.4_	Olfml3	blue	3.7191052
scl16334.7.:	Dars	blue	3.6913402
scl11702.1.:	BC002199	blue	3.6550228
scl016206.3	Lrig1	blue	3.6184685
scl47579.6.:	B930062P2	blue	3.3474832
GI_3809085	LOC235979	blue	3.2077372
scl018293.8	Ogdh	blue	3.0937925
GI_2087832	1100001F1(	blue	3.0362247
scl43124.28	Daam1	blue	2.9291557
scl37836.1.:	4930533K1{	brown	53.522953
scl31483.18	4931406P1(	brown	45.720463
scl0110323.	Cox6b	brown	45.120598
scl027176.1	Rpl7a	brown	45.007266
scl30996.1.:	2900057K0(	brown	44.228067
scl37395.7.:	Dtx3	brown	44.081734
GI_8394132	Rab33b	brown	43.235631
scl19005.12	Slc39a13	brown	42.478719
scl0003799.1_2-S		brown	42.324861
scl066212.2	Sec61b	brown	42.276135
scl40597.27	Smtn	brown	41.568336
scl37429.8_	D430026P1	brown	41.429402
scl066144.2	Atp6v1f	brown	40.933218
scl071435.1	Arhgap21	brown	39.940281
scl39417.12	Pitpnc1	brown	39.585167
GI_3808679	LOC382247	brown	39.539792
GI_3808999	LOC382092	brown	39.463736
ri|F630038C	F630038O1	brown	39.430889
scl41723.13	G431001E0	brown	38.194997
scl36897.12	1200015E1	brown	37.5715
scl40823.31	Mrc2	brown	37.363666
ri|C330003C	Tcerg1	brown	37.288105
scl0056428.	Mtch2	brown	37.232028
scl33603.4_	Ptger1	brown	36.92304
scl14296.1.:	Itpkb	brown	36.796089
scl012462.9	Cct3	brown	36.697576
scl38577.23	E030041M2	brown	36.375107
scl53734.13	Tro	brown	36.148731
GI_3808731	AI448196	brown	35.970206
scl54289.1.:	BC042423	brown	35.899099
scl068763.4	1110038B1:	brown	35.843625
scl0001763.	Slc35b1	brown	35.623978
scl25365.64	Col27a1	brown	35.116278
scl29434.9.:	Ddx47	brown	34.999562
scl27024.7_	Fscn1	brown	34.986333
scl0068202.	Ndufa5	brown	34.967942
scl21329.11	Frmd4a	brown	34.873926

scl072362.1	2210415K03	brown	34.827714
scl0003832.	2900091E11	brown	34.620323
scl24799.82	Hspg2	brown	34.556023
scl0002631.	Eif3s2	brown	34.542584
scl00225215	BC003885	brown	34.499152
scl41210.3.1	Sdf2	brown	34.230093
ri|0610009J0	0610009J05	brown	34.204859
scl41183.17	Rab11fip4	brown	34.174691
scl0227648.	AU024582	brown	33.95178
GI_3474033	Tuba2	brown	33.414153
scl068647.2	1110020P09	brown	33.374528
scl33268.1.9	5830471E12	brown	33.372826
scl015387.1	Hnrpk	brown	33.114242
scl069236.1	2610034E01	brown	32.832164
ri|A730089E	A730089E0	brown	32.716763
scl35368.9_	Gnai2	brown	32.554311
scl0224904.	2410015M20	brown	32.260615
scl0027008.	D15Mit260	brown	32.116532
scl0320066.	C230052J16	brown	32.100725
GI_3808964	LOC215678	brown	32.012466
ri|2310007G	2310007G05	brown	31.94869
scl075730.1	Supt6h	brown	31.917926
GI_3807499	LOC382790	brown	31.862631
scl076808.4	Rpl18a	brown	31.809039
ri|A130062D	A130062D1	brown	31.647927
GI_3807459	LOC269251	brown	31.475276
scl0016494.	Kcna6	brown	31.284077
scl0003171.	Psmb7	brown	31.108133
ri|D130027C	Msi2h	brown	31.080707
ri|6330578B	6330578B10	brown	31.033414
scl0003742.	Tmem14c	brown	30.989266
GI_2212248	Chd3	brown	30.325705
scl25844.10	AA407930	brown	30.094125
scl0107686.	Snrpd2	brown	30.086236
scl27493.5_	E430036I04	brown	30.022203
scl46895.1.1	C430045I18	brown	30.013738
scl37941.2.4	LOC215999	brown	29.97468
scl0002315.	1_12-S	brown	29.940893
scl23529.21	Plod1	brown	29.843716
ri|B930044G	B930044G1	brown	29.841277
scl35722.2_	Fem1b	brown	29.795749
scl37833.23	Bicc1	brown	29.622895
scl0217342.	B230113M0	brown	29.561238
scl0226154.	Lzts2	brown	29.519341
scl0027050.	Rps3	brown	29.44165
scl0001742.	Ppard	brown	29.426391
scl054217.2	Rpl36	brown	29.136785
ri|D030032G	D030032G0	brown	29.039358
scl000534.1	Ppp1ca	brown	28.852352
scl0014937.	Gys3	brown	28.697164
GI_4538391	Elk3	brown	28.688582
scl00320151	5330432J10	brown	28.651764
scl50451.21	Eml4	brown	28.556287
scl22489.6_	Ddah1	brown	28.321367

scl44632.16	C130052I12	brown	28.315462
GI_3804941	LOC380749	brown	28.291823
scl0020923.	Supt4h2	brown	28.173381
GI_3808949	E130305N2	brown	28.13824
scl21990.8.1	Prcc	brown	27.88187
scl41548.2.1	Hint1	brown	27.80025
scl075994.1	5033421J10	brown	27.598693
scl018951.1	5-ก.ย.	brown	27.544451
GI_3808260	LOC381735	brown	27.447052
scl0270118.	BC032967	brown	27.412181
scl000094.1	Mea1	brown	27.342097
scl39368.13	2610035D17	brown	27.207316
scl38883.14	Ascc1	brown	27.203382
ri|4832408C	4832408C21	brown	27.202388
ri|A530021P	A530021P13	brown	27.08075
scl36750.29	Myo1e	brown	26.886871
scl28879.24	Jmjd1a	brown	26.803819
scl40603.7.4	Drg1	brown	26.726306
GI_3808712	C130002K14	brown	26.631562
ri|4933407H	4933407H13	brown	26.503702
scl0021413.	Tcf4	brown	26.489847
scl066340.3	1700023M09	brown	26.446255
scl24607.15	Dvl1	brown	26.350555
ri|5730512J0	5730512J02	brown	26.326254
scl0013386.	Dlk1	brown	26.313839
scl37450.6_	D10Ertd516	brown	26.27597
scl000287.1	AI043088	brown	26.234336
scl31612.8.1	Egln2	brown	26.145932
scl49529.6.1	Pigf	brown	26.004713
scl20479.1.6	Nola3	brown	25.968598
GI_3808814	LOC384727	brown	25.729194
scl50835.68	Col11a2	brown	25.693155
scl011816.1	Apoe	brown	25.647765
scl0234854.	Cdk10	brown	25.612951
scl24771.1.2	C79267	brown	25.574891
scl53382.12	Fads3	brown	25.420923
GI_3807909	LOC230896	brown	25.414136
GI_4247634	Rplp2	brown	25.368675
scl38547.21	Hal	brown	25.009695
scl0328962.	9130229N11	brown	24.988965
scl17334.16	Rgpr	brown	24.805959
scl37369.2.6	Rbms2	brown	24.764823
scl0056330.	Pdcd5	brown	24.693825
scl0319880.	C630016B2	brown	24.618551
scl073661.1	2210419D22	brown	24.565578
scl0022134.	Tgoln1	brown	24.539751
scl45347.17	Rai16	brown	24.530609
scl35519.13	Syncrip	brown	24.29983
scl26541.1.1	C530043K16	brown	24.043071
scl19537.3.2	C330006A16	brown	23.962153
scl067118.7	Bfar	brown	23.925774
scl45724.3.1	2200001I15	brown	23.88132
scl20223.6.1	5430433G2	brown	23.805474
scl46200.4_	A030013D2	brown	23.762212

scl056334.4	1110032D12	brown	23.746493
scl15880.9_	4933426L22	brown	23.630585
scl052468.3	Ctdsp2	brown	23.609242
scl072722.1	2810405J04	brown	23.553746
scl25784.5.1	Pdap1	brown	23.530609
scl066943.7	2310009N05	brown	23.285452
scl28393.5.1	Kcna6	brown	23.276177
GI_3807566	LOC241621	brown	23.23898
ri|2700046G	2700046G09	brown	23.220416
scl51416.9_	Lox	brown	23.219769
scl30452.18	Nap1l4	brown	23.084814
ri|4732470M	4732470M2	brown	22.65875
scl49286.1_	Lpp	brown	22.564876
scl0107095.	1110004P15	brown	22.556391
scl0011307.	Abcg1	brown	22.520144
scl0227613.	4930542G03	brown	22.471225
scl0070351.	Ppp4r1	brown	22.422873
scl0014062S	C330018L13	brown	22.382777
scl40219.8.1	Pdlim4	brown	22.31677
scl23930.5.1	Atp6v0b	brown	22.267819
scl0218490.	Btf3	brown	22.258946
scl39869.11	Wsb1	brown	22.25028
scl50710.7_	Tnfrsf21	brown	22.171521
scl0004207.	Fastk	brown	22.108801
scl34998.4_	B230378H1	brown	22.106054
scl0083814.	Nedd4l	brown	22.100537
scl52526.2_	Ppp1r3c	brown	22.067067
ri|E430030L	E430030L01	brown	22.035839
scl0001591.	Hnrph1	brown	21.870554
scl50686.4.1	Mrpl14	brown	21.840764
scl021885.1	Tle1	brown	21.836248
ri|A130098G	A130098G0	brown	21.647527
scl066177.1	Ubl5	brown	21.600432
scl32699.8.1	Rras	brown	21.581585
scl20023.12	Dlgap4	brown	21.50831
scl093695.1	Gpnmb	brown	21.487247
scl0001217.	Calu	brown	21.483502
scl53508.17	LOC114601	brown	21.479599
scl17448.26	Jarid1b	brown	21.477859
ri|1700024P	Spnr	brown	21.452563
scl0057266.	Cxcl14	brown	21.335098
scl27103.18	Ephb4	brown	21.279049
scl31603.36	Ltbp4	brown	21.271774
scl0003628.	Ndufs4	brown	21.249538
scl36702.4_	Arpp19	brown	21.228863
scl0065970.	D15Ertd366	brown	21.207317
scl53122.4_	Ubtd1	brown	21.163432
ri|A230057M	A230057M0	brown	21.011376
scl0023825.	Banf1	brown	20.997201
scl017178.4	Fxyd3	brown	20.968235
scl32936.22	Cic	brown	20.850069
GI_2851046	Wdt3-pendir	brown	20.63122
scl49248.16	Mfi2	brown	20.6161
scl28067.9_	AI847670	brown	20.582221

scl0073733.	Sbsn	brown	20.548799
scl069202.1	2610009E16	brown	20.524001
scl17740.13	Hrb	brown	20.497816
scl31564.8.5	1200009C21	brown	20.487062
scl0269523.	Vcp	brown	20.439609
ri|2010009J1	2010009J12	brown	20.38971
scl0170658.	Ndufs5	brown	20.2539
ri|4732435K	4732435K05	brown	20.192464
ri|5830431I1	5830431I15	brown	20.115765
scl0245688.	Rbbp7	brown	20.106139
scl40204.6.1	Atox1	brown	20.060204
GI_3218931	Vim	brown	19.981864
scl068837.8	1110054H05	brown	19.903749
GI_3809048	LOC380634	brown	19.898009
scl38374.10	0610007H07	brown	19.653222
scl19721.5_	5430407P10	brown	19.648309
scl35459.1.3	2900078C09	brown	19.590591
scl0003424.	Tgfbr2	brown	19.557935
scl21916.1_	S100a1	brown	19.351104
scl27165.9_	Rabgef1	brown	19.350727
scl32752.7.1	Etfb	brown	19.332097
scl31480.8_	Kctd15	brown	19.250311
scl41622.18	Gfpt2	brown	19.092361
scl37319.3_	8430410K20	brown	18.990332
scl33725.12	Fkbp8	brown	18.878941
ri|A330027G	H2-T18	brown	18.866438
scl076846.5	3010033P07	brown	18.784237
GI_3808100	LOC385992	brown	18.629522
scl014227.3	Fkbp2	brown	18.590398
scl23441.8_	Ski	brown	18.51601
scl38867.11	Sara1	brown	18.446154
scl071679.3	Atp5h	brown	18.174605
ri|2810004A	Hsd17b12	brown	18.135194
scl37287.1.1	Phxr4	brown	18.123848
scl080795.5	Selk	brown	18.112356
scl29741.15	Slc6a6	brown	18.094394
scl44795.7_	Bicd2	brown	18.030784
ri|0610010I0	0610010I05	brown	17.974071
scl31250.6.4	Klf13	brown	17.883318
scl053607.7	Snrpa	brown	17.834427
scl52590.16	D19Wsu12e	brown	17.820853
scl0027368.	Tbl2	brown	17.795275
scl21477.25	Nfkb1	brown	17.67469
scl36111.9.1	Rp9h	brown	17.666518
ri|E430026C	E430026C0	brown	17.655625
scl22876.3_	Mcl1	brown	17.628827
GI_3807557	LOC382843	brown	17.587035
GI_3808256	LOC383249	brown	17.561331
scl020717.5	Serpina3m	brown	17.519988
scl34233.12	Slc7a5	brown	17.432618
ri|9030215D	Enah	brown	17.416066
scl29213.18	Impdh1	brown	17.409367
scl38227.11	Ust	brown	17.366636
scl068778.1	1110038D17	brown	17.148607

scl018824.1	Plp2	brown	17.124913
scl37679.21	Dip3b	brown	17.11934
scl071766.1	1300006N24	brown	17.012568
scl0083921.	Tmem2	brown	16.941454
scl0212528.	D8Ertd812e	brown	16.914544
scl29154.3_	BC064033	brown	16.8364
scl27764.24	B3bp	brown	16.672712
scl00233908	Fus	brown	16.612609
scl53028.7_	Trim8	brown	16.577917
scl32920.6.1	Tgfb1	brown	16.519024
scl020116.3	Rps8	brown	16.346169
scl011747.1	Anxa5	brown	16.279163
scl43391.2_	Rdh14	brown	16.236471
scl0233835.	Tnrc6	brown	16.224079
scl26208.17	Sez6l	brown	16.191471
GI_3807486	LOC218482	brown	16.149081
scl50754.6_	Gna-rs1	brown	16.113085
scl0003908.	Sgk	brown	16.097615
ri|8430403J1	8430403J19	brown	16.09652
scl12335.4.1	4933404K08	brown	16.068309
ri|4930579N	4930579N16	brown	15.926064
scl023872.1	Ets2	brown	15.92429
scl0027492.	D0Kist3	brown	15.872879
scl30070.11	Gpnmb	brown	15.843881
scl078772.9	4930507C10	brown	15.767744
scl41447.1.2	2410006H16	brown	15.677713
scl48225.31	Tiam1	brown	15.560989
scl012226.1	Btg1	brown	15.485797
scl29417.6.1	Pde6h	brown	15.478864
GI_3808116	LOC386117	brown	15.420956
scl22088.5.1	Lxn	brown	15.36294
scl47186.4_	Has2	brown	15.2975
scl50429.12	Ppm1b	brown	15.2868
scl39030.3_	Col10a1	brown	15.279897
scl066390.1	2310042G06	brown	15.277535
scl0103841.	Cuedc1	brown	15.254841
scl0066395.	2310047C17	brown	15.237681
scl012840.3	Col9a2	brown	15.192399
scl30988.16	2700017M0	brown	15.172515
scl000766.1	Pde6d	brown	15.089051
scl18118.39	Col9a1	brown	15.000481
scl18363.8_	Sdc4	brown	14.809612
scl0003450.	Cklfsf7	brown	14.773685
GI_3809341	LOC385062	brown	14.68398
scl40534.12	Tbrg4	brown	14.645597
scl0002405.	Kns2	brown	14.518015
GI_3808076	LOC385849	brown	14.466984
scl26662.1_	6820424L24	brown	14.437463
scl0003913.	Cdk4	brown	14.408272
scl32572.22	Pace4	brown	14.362654
ri|1110034G	D2Bwg1335	brown	14.272432
scl0053607.	Snrpa	brown	14.189764
scl17033.25	Lamb3	brown	14.093648
scl014376.1	Ganab	brown	14.064356

ri|9630041B	9630041B1:	brown	14.022639
scl0001950.	Tpm3	brown	13.961779
ri|1500002F	Cops5	brown	13.903659
scl51549.11	Dp1	brown	13.896221
GI_3808340	LOC381132	brown	13.799942
scl40084.1.:	AW536289	brown	13.699513
ri|B130008D	B130008D2	brown	13.572834
GI_3808096	LOC385959	brown	13.537776
scl0059013.	Hnrph1	brown	13.537419
scl020610.4	Sumo3	brown	13.40602
scl48393.15	Nfkbiz	brown	13.322756
scl37357.2_	2210411K19	brown	13.24978
GI_3808791	LOC382303	brown	13.248818
scl011821.1	Aprt	brown	13.152411
GI_3809027	LOC234987	brown	13.030762
scl47198.5_	Tnfrsf11b	brown	12.963044
scl021770.4	Ppp2r5d	brown	12.90216
ri|2810434H	Idb2	brown	12.883721
scl22801.5.:	Ngfb	brown	12.843888
scl000558.1	Fts	brown	12.83155
GI_3808135	LOC386268	brown	12.824154
scl32598.21	Atp10a	brown	12.632477
scl47805.7_	Grina	brown	12.553743
scl098952.1	C230093N1	brown	12.451537
GI_3808143	LOC386330	brown	12.442084
GI_3809403	LOC382177	brown	12.393034
scl33196.2.:	Rhou	brown	12.325799
GI_3808134	LOC386256	brown	12.173263
ri|B230114H	B230114H0	brown	12.131979
scl0002187.	Rab18	brown	12.047426
scl48626.2.:	2900064B18	brown	11.982527
ri|F730003H	F730003H07	brown	11.845487
GI_8801473	Lif	brown	11.809359
GI_3809059	B230104P2:	brown	11.628797
GI_3808120	LOC386124	brown	11.592194
scl026968.1	Islr	brown	11.53294
ri|B230386D	B230386D1	brown	11.453315
scl22018.3_	Tlr2	brown	11.174654
GI_3808113	LOC386085	brown	11.158661
scl0227624.	B230208H1	brown	11.151969
scl000813.1	Dst	brown	11.104281
ri|4932441P	4932441P04	brown	11.05775
scl056361.2	Pus1	brown	11.04792
scl020229.1	Sat1	brown	11.038479
scl38735.7_	Pttg1ip	brown	10.925136
scl070333.1	Ase1	brown	10.923351
scl0338522.	9230115A19	brown	10.718244
scl49287.19	Lpp	brown	10.576331
GI_3808519	LOC381230	brown	10.524996
GI_3808115	LOC386101	brown	10.521804
scl44467.12	Mrps27	brown	10.510719
scl42144.4_	Gpr68	brown	10.498896
ri|A430045K	A430045K0	brown	10.476811
scl32778.3_	Zfp537	brown	10.343311

scl46503.3.1	Mustn1	brown	10.232905
scl0195018.	Zzef1	brown	10.18103
scl21320.10	Nudt5	brown	9.9236831
scl0021216{	Zswim4	brown	9.891875
scl24621.11	Gnb1	brown	9.7917832
scl000094.1	Ppp2r5d	brown	9.7557496
scl29852.1_	Mrpl53	brown	9.5905604
scl52151.4.1	2610028L19	brown	9.5082575
GI_3808088	LOC385923	brown	9.4843652
scl46370.4_	Apex1	brown	9.4810581
GI_3808128	LOC386199	brown	9.4336414
scl000980.1	Mcm6	brown	9.3593478
scl0002238.	2610028L19	brown	9.3425315
ri|E330027P	E330027P0(	brown	9.3289093
scl096875.3	Prg4	brown	9.2943259
scl30036.21	Tax1bp1	brown	9.2735556
scl0004065.	Abhd1	brown	9.1613583
scl53274.2_	Bteb1	brown	8.982953
scl0003674.	Homer1	brown	8.9706747
scl33438.2.1	4833426J09	brown	8.7391979
scl50961.11	Axin	brown	8.6347594
ri|2010006A	3000003G13	brown	8.6144615
scl23145.17	Mbnl1	brown	8.5847231
scl22956.4.1	Jtb	brown	8.5416042
scl25032.12	Slc2a1	brown	8.4634143
scl0012729.	Clns1a	brown	8.4023712
scl075623.2	1700029F09	brown	8.3622905
scl0232334.	Vgll4	brown	8.2725448
scl023886.1	Gdf15	brown	8.114194
scl33669.19	Sin3b	brown	8.0994994
scl33166.4_	Kcnk1	brown	8.0474775
scl38335.7.1	Mettl1	brown	7.9920896
scl37236.8_	Icam1	brown	7.9264963
scl41544.8_	Gpx3	brown	7.9105863
scl0002272.	2610028L19	brown	7.9060935
scl32499.13	Abhd2	brown	7.9031163
GI_3808425	LOC384382	brown	7.7862743
scl17703.10	1700027L20	brown	7.6714704
scl0214359.	BC003277	brown	7.6424181
scl000973.1	AA407809	brown	7.6361061
scl014852.1	Gspt1	brown	7.5914002
ri|C330001N	C330001N2	brown	7.5829615
scl47694.7_	2310042L06	brown	7.5396673
scl052364.1	D5Ertd591e	brown	7.5197268
scl41743.2_	Rtn4	brown	7.5195318
scl15984.9.1	AA407809	brown	7.5151686
scl20814.13	4933404M0	brown	7.5144704
scl44485.3_	Enc1	brown	7.3894589
ri|1110068E	1110068E08	brown	7.3800439
scl0077945.	Rpgrip1	brown	7.3553727
scl11590.1.1	B230207M2	brown	7.3382763
scl020778.1	Scarb1	brown	7.2691484
scl38233.1.3	6430537I21	brown	7.2321764
scl0023550	Gsta2	brown	7.2300041

scl0071428.	5830407P18	brown	7.2133111
scl0064898.	Lpin2	brown	7.1998504
scl49488.8_	Zfp263	brown	7.179771
scl39078.31	Crsp3	brown	7.1476632
scl0021786	D12Wsu95e	brown	7.1082122
scl067263.1	Zswim6	brown	7.0879369
scl40166.10	Guk1	brown	6.9681809
GI_3807554	LOC218696	brown	6.9595576
scl39074.27	Epb4.1l2	brown	6.9059867
scl26674.8_	Wfs1	brown	6.8964997
ri|5730577G	5730577G1	brown	6.7832284
ri|6330404C	6330404C0	brown	6.7818276
scl50203.3_	Gfer	brown	6.7414017
scl45293.12	Gtf2f2	brown	6.7313247
scl52675.8.	Ostf1	brown	6.6806409
GI_3807485	LOC383712	brown	6.5858693
scl33502.12	Mmp2	brown	6.5727674
scl33833.8_	Dctd	brown	6.5726359
ri|7420404C	7420404O0	brown	6.5208422
scl32698.6.	Irf3	brown	6.4495837
ri|B230334I	Srr	brown	6.4350889
scl5203.1.1_	C030027H1	brown	6.382969
scl45530.16	Tgm1	brown	6.2355129
scl50763.13	Flot1	brown	6.2112735
scl059069.6	Tpm3	brown	6.0231919
scl34499.5.	Irx3	brown	5.9681408
scl0067398.	Srpr	brown	5.9639595
scl0016599.	Klf3	brown	5.9284345
scl0330474.	BC057627	brown	5.9261135
scl36159.7.	Angptl6	brown	5.9226559
GI_3808267	LOC381739	brown	5.8170567
scl073354.1	1700052O2	brown	5.8045836
ri|D130058C	Ddx26	brown	5.6771788
scl36546.14	Slco2a1	brown	5.6702462
scl18384.2_	D930001I22	brown	5.6179825
scl0002954.	Rbm3	brown	5.589195
scl22250.2.	1700017M0	brown	5.5736402
scl0269704.	AW060232	brown	5.5083298
ri|A830036H	Srr	brown	5.5017657
scl45598.20	Ndrg2	brown	5.4657587
scl36966.5.	Cryab	brown	5.4003624
scl069806.1	Slc39a11	brown	5.3837038
scl30454.4.	Phlda2	brown	5.3692337
scl34176.2.	2310031A18	brown	5.3644813
GI_3154370	Siat7b	brown	5.3216262
scl42571.13	Ttc15	brown	5.3031965
ri|D330004C	D330004C0	brown	5.2701017
scl38510.3.	Lum	brown	5.2633576
scl020019.2	Rpo1-4	brown	5.2125768
scl45529.12	Rabggta	brown	5.1954207
scl41556.19	A730024A0	brown	5.1334076
scl015980.8	Ifngr2	brown	5.1048124
scl0003335.	3632413B0	brown	5.0649414
scl41136.13	Taf15	brown	5.0253172

scl0014696.	Gnb4	brown	5.0039629
ri|A130070G	A130070G0	brown	4.9492322
scl0003433.	Islr	brown	4.9245991
ri|C230043G	C230043G0	brown	4.8186582
scl40216.4.1	Csf2	brown	4.7698771
scl072752.1	2810438F06	brown	4.7694127
ri|A830006C	Aldh1a3	brown	4.6915278
scl18491.1.2	Fkhl18	brown	4.5837912
scl000572.1	Cnot7	brown	4.4827938
scl000513.1	6030443O07	brown	4.38544
scl0072098.	2010300G19	brown	4.3484839
scl0003580.	Tm4sf8	brown	4.3388671
scl20917.35	Fmnl2	brown	4.1592114
GI_6679402	Ppp1r14b	brown	4.0781741
scl0319600.	Usp37	brown	4.0722558
ri|1810010H	4933439C20	brown	3.9568386
ri|B230334I0	B230334I05	brown	3.8708345
scl00210530	Leprel1	brown	3.7224571
scl0018130.	Ddx26	brown	3.6680169
scl012389.5	Cav1	brown	3.6521951
ri|B230354O	B230354O1	brown	3.6255466
scl26598.18	Gpr125	brown	3.2373183
scl26184.7_	Kctd10	brown	3.2357989
ri|2810049C	2810049C16	brown	3.1772146
scl0110796.	Sdccag33	brown	2.9981006
scl0074349.	4632419K20	yellow	63.592005
scl48080.5_	C1qtnf3	yellow	62.817585
scl0098878.	Ehd4	yellow	61.104755
scl50037.4.1	H2-Ke2	yellow	60.878365
ri|1600029K	Gng12	yellow	59.659977
scl39079.4_	Ctgf	yellow	59.318362
scl30730.6_	Cdr2	yellow	59.308794
scl30498.4_	Hras1	yellow	59.159817
ri|0710001I1	Atp5f1	yellow	58.89274
scl0229543.	C77668	yellow	57.960615
scl23588.20	Plekhm2	yellow	57.723244
scl45410.6_	Scara3	yellow	57.564794
scl012859.1	Cox5b	yellow	56.97623
scl066445.5	Cyc1	yellow	56.056922
scl071994.3	Cnn3	yellow	54.980827
scl16816.7.1	Fhl2	yellow	54.107766
scl020250.1	Scd2	yellow	53.814474
scl30605.20	Fgfr2	yellow	53.653007
scl40331.6_	Ccng1	yellow	53.435945
scl33924.6.9	Ppp2cb	yellow	53.19969
ri|1110055C	1110055O2	yellow	53.025599
GI_3808710	LOC385467	yellow	52.473935
scl20107.1_	Idb1	yellow	52.161517
scl0242083.	Ppm1l	yellow	51.952572
scl026893.9	Cops6	yellow	51.734844
scl056438.5	Rbx1	yellow	50.835878
scl27712.5_	Rasl11b	yellow	50.788162
scl051792.1	Ppp2r1a	yellow	50.44364
scl28417.13	Leprel2	yellow	50.377505

scl0066350.	Pla2g12a	yellow	50.078532
GI_3805055	LOC268569	yellow	50.075691
scl45684.12	Ctnnb1	yellow	49.823236
scl0001235.	Pex5	yellow	48.762829
scl018858.5	Pmp22	yellow	48.239732
GI_2153959	Ifitm3	yellow	48.230118
scl0003202.	Gpiap1	yellow	47.9944
GI_3807958	4933427G23	yellow	47.419499
scl0010147(	AA960558	yellow	47.315935
GI_3808907	LOC234081	yellow	47.314582
scl26505.24	Corin	yellow	47.165568
scl011544.1	Adprh	yellow	47.071888
scl16475.25	Per2	yellow	47.066481
scl53996.13	Slc7a3	yellow	47.060602
scl058894.2	4732460K03	yellow	46.954859
scl22884.7.1	Lass2	yellow	46.58255
scl0268741.	5730589K01	yellow	46.469305
scl0003636.	Tgfbi	yellow	46.29099
scl43488.6_	Snag1	yellow	46.254589
scl0012831.	Col5a1	yellow	45.719865
scl0021961.	Tns	yellow	45.612838
scl33050.3.1	1810008O2	yellow	45.524913
scl0068682.	1110028E10	yellow	45.341157
scl0002200.	Nfatc1	yellow	44.846133
scl0024208:	Ppm1l	yellow	44.031714
scl0066494.	2610524G07	yellow	43.944563
scl0016991.	Lt1	yellow	43.8782
GI_3808622	LOC243912	yellow	43.600881
scl31608.7.1	Itpkc	yellow	43.560809
scl37379.15	Shmt2	yellow	43.518355
scl0018018.	Nfatc1	yellow	43.268978
scl30707.21	Arhgap17	yellow	43.035436
scl0004022.	Arpc1b	yellow	42.857764
GI_3808653	LOC236932	yellow	42.293828
ri|E130112E	E130112E08	yellow	41.661677
scl020910.1	Stxbp1	yellow	41.476088
scl022021.6	Tpst1	yellow	41.39216
scl068713.2	Ifitm1	yellow	41.38242
scl0039443:	Ugt1a10	yellow	40.854803
scl27010.17	Pscd3	yellow	40.723632
ri|2610030F	Set	yellow	40.48403
scl0037386	Col27a1	yellow	40.290206
scl076650.2	Npn3	yellow	40.133364
scl36804.10	Spg21	yellow	40.002191
scl42057.12	Wars	yellow	39.971554
scl0002010.	Pla2g12a	yellow	39.915118
scl28431.17	Pex5	yellow	39.864641
scl0012808.	Cobl	yellow	39.558931
scl41368.8.9	Trp53	yellow	39.540209
scl16279.4_	Prelp	yellow	39.473335
GI_3807415	LOC380828	yellow	39.421141
scl057267.1	Apba3	yellow	39.348045
scl15964.10	Hsd17b7	yellow	39.320989
scl24615.5.1	1500011L16	yellow	39.298238

scl25288.8_	Mtap	yellow	39.24924
GI_3809073	LOC231046	yellow	39.15166
GI_3808503	LOC381808	yellow	38.917655
scl0020661.	Sort1	yellow	38.916157
scl23674.9_	A330049M0	yellow	38.583648
scl0056490.	Zfp288	yellow	38.537159
scl066375.2	Dhrs7	yellow	38.523966
scl076650.2	Npn3	yellow	38.474287
ri|2700079C	Ptpn2	yellow	38.468814
scl0216164.	Dos	yellow	38.423422
scl000041.1	Ppp1ca	yellow	38.217947
scl31408.4_	Atf5	yellow	37.928946
scl0003542.	Smfn	yellow	37.377286
scl29997.10	Fkbp9	yellow	37.172497
scl0002605.	Bach	yellow	37.163459
scl38595.2_	C630002B1	yellow	37.107634
scl53365.18	Prp19	yellow	36.817491
scl026441.8	Psma4	yellow	36.720685
scl29883.14	Ggcx	yellow	36.609267
scl25380.5_	Slc31a1	yellow	36.222688
scl0001373.	Sumo2	yellow	35.840931
GI_3809132	LOC380692	yellow	35.647202
scl40930.5.	Csf3	yellow	35.614624
scl014870.5	Gstp1	yellow	35.606547
scl0011973.	Atp6v1e1	yellow	35.523281
scl0015568.	Elavl1	yellow	35.399974
scl30749.5_	Gprc5b	yellow	34.979238
scl056405.2	Dusp14	yellow	34.918634
scl54036.9.	Maged1	yellow	34.070575
scl52314.6.	Prdx3	yellow	33.852995
scl0078558.	Htra3	yellow	33.558876
scl27882.4.	4930443F0	yellow	33.490061
scl0014560.	Gdf10	yellow	33.302219
scl30506.2_	Ifitm3	yellow	33.249202
scl18620.21	2310032D1	yellow	32.794808
scl0023972.	Papss2	yellow	32.759282
scl18232.3.	Psma7	yellow	32.642606
scl43090.8_	Rhoj	yellow	32.452538
scl067539.1	4931431C0	yellow	32.407439
scl42747.5_	6720458F0	yellow	32.270171
scl37617.5_	Slc25a3	yellow	32.217345
scl37309.10	Mmp13	yellow	32.116859
scl0017289.	Mertk	yellow	31.851917
scl0023553	C130099A2	yellow	31.848135
scl34524.3_	Siah1a	yellow	31.670903
scl068792.1	Srpx2	yellow	31.385697
scl43661.2_	F2r	yellow	31.257494
scl39616.9.	Nr1d1	yellow	30.899534
scl084652.1	Drctnnb1a	yellow	30.70976
scl26736.8.	Ppm1g	yellow	30.570147
scl31901.9_	Psmd13	yellow	30.523726
scl25152.5.	Magoh	yellow	30.517521
GI_3809100	LOC380682	yellow	30.426195
scl23697.3.	Sh3bgrl3	yellow	30.244354

scl0065960.	Twsg1	yellow	30.119871
scl49316.10	Dnajb11	yellow	29.925211
scl080876.2	Ifitm2	yellow	29.762063
scl22560.5_	Ddit4l	yellow	29.720848
scl34595.13	Gab1	yellow	29.66056
scl40126.18	Epn2	yellow	29.653123
scl31226.15	Mef2a	yellow	29.62193
scl0012334.	Capn2	yellow	29.546024
GI_3808354	LOC381140	yellow	29.478559
scl012334.3	Capn2	yellow	29.332991
GI_3808519	LOC240672	yellow	29.186192
scl37305.10	Mmp10	yellow	29.16333
ri|D630024C	D630024O0	yellow	29.148182
scl0002748.	1110060F11	yellow	29.148153
scl0011852.	Rhob	yellow	29.029145
scl0209011.	Sirt7	yellow	29.020445
scl31417.6_	Scgf	yellow	28.982026
scl0010098	Akap9	yellow	28.926328
scl0014870.	Gstp1	yellow	28.885069
scl067509.1	1810063B07	yellow	28.674054
GI_3807775	LOC383964	yellow	28.670683
scl0268373.	Ppia	yellow	28.607322
scl37892.17	Ddx50	yellow	28.214683
scl014113.7	Fbl	yellow	28.169056
scl16685.5_	Klf7	yellow	28.063377
ri|A230067E	A230067E15	yellow	28.044922
scl20830.5_	Dhrs9	yellow	27.966075
scl54331.2.4	Ndufa1	yellow	27.926199
scl50798.28	Vars2	yellow	27.822173
scl0003775.	Shmt2	yellow	27.717317
scl0319219.	5330401F18	yellow	27.661754
scl015903.3	Idb3	yellow	27.639297
scl34375.12	Smpd3	yellow	27.515962
scl52114.2_	Egr1	yellow	27.100895
scl018643.4	Pfn1	yellow	26.960756
scl44710.17	Tgfbi	yellow	26.4632
scl26049.1.1	Gpr109b	yellow	26.425448
scl0001709.1_980-S		yellow	26.381886
GI_3808990	LOC235497	yellow	26.292319
ri|E130318A	E130318A13	yellow	26.239581
scl40050.12	Ndel1	yellow	26.234497
scl18092.6_	D1Wsu40e	yellow	26.1208
scl29140.1.1	Creb3l2	yellow	26.113712
scl020377.1	Sfrp1	yellow	26.023443
scl46955.19	B230369L08	yellow	25.885784
scl18267.7.1	Bmp7	yellow	25.779337
scl51511.5_	Dtr	yellow	25.448137
scl019166.8	Psma2	yellow	25.44336
scl0057261.	Brd4	yellow	25.342231
scl30657.16	Mvp	yellow	25.307708
scl47561.6_	4930570C03	yellow	25.232891
scl27205.18	Aacs	yellow	25.229467
scl013885.9	Esd	yellow	25.162833
scl36991.12	Igsf4a	yellow	25.153797

scl019175.6	Psmb6	yellow	25.148222
scl23864.14	1700018O1	yellow	24.942136
scl28767.1.1	Rab11fip5	yellow	24.669424
scl27894.1.9	2600003E2	yellow	24.660086
scl42729.13	Adssl1	yellow	24.655337
scl47631.9.1	5730592L21	yellow	24.647348
scl39694.29	Itga3	yellow	24.506196
scl052530.1	Nola2	yellow	24.489846
scl017776.1	Mast2	yellow	24.391192
scl42787.13	Gtl2	yellow	24.38557
scl15816.17	Capn2	yellow	24.110424
scl15745.2.1	G0s2	yellow	24.076387
GI_3808620	LOC381865	yellow	23.970203
scl44283.11	Tm7sf1	yellow	23.706953
scl48729.15	Mcm4	yellow	23.642648
GI_6754695	Mif	yellow	23.637125
scl000082.1	Nr1d1	yellow	23.547771
scl0003203.	3632413B0	yellow	23.448
scl0073046.	2900070E1	yellow	23.217084
scl47854.5_	Wisp1	yellow	23.121295
scl0066546.	2010010M0	yellow	22.899502
scl017532.3	Mras	yellow	22.835963
scl33046.8.1	Slc1a5	yellow	22.590478
scl28384.6_	Ccnd2	yellow	22.498894
scl000612.1	Psmd7	yellow	22.488325
scl075007.4	4930504E0	yellow	22.445362
scl0010868	9530090G2	yellow	22.442478
scl42783.1_	1110006E1	yellow	22.427771
scl45268.2.9	A130038J17	yellow	22.37287
scl52762.14	Fads2	yellow	22.171103
scl46587.11	Plau	yellow	22.164033
scl0027407.	Abcf2	yellow	22.131497
scl0014869.	Gstp1	yellow	22.054147
scl44819.5_	C78339	yellow	21.708687
scl000226.1	Mrpl48	yellow	21.574197
scl28023.13	Galnt11	yellow	21.532276
scl51346.13	9430028L0	yellow	21.521986
scl36974.5_	1600029D2	yellow	21.034914
scl074522.7	Zcwcc1	yellow	20.899046
scl0083924.	Tm7sf1	yellow	20.872561
scl37659.18	Tra1	yellow	20.794362
scl0068797.	Pdgfrl	yellow	20.700741
ri|D930008C	D930008G0	yellow	20.592696
scl0012226.	Btg1	yellow	20.565686
scl52608.7.1	E330013K2	yellow	20.51711
scl0394435.	Ugt1a9	yellow	20.405834
GI_3808682	LOC381891	yellow	20.397247
scl52415.12	Ldb1	yellow	20.396367
scl080751.6	Rnf34	yellow	20.178468
scl33440.8_	Cklfsf3	yellow	20.162114
ri|A530032J	A530032J1	yellow	20.03707
scl22366.7.1	Cyp7b1	yellow	19.926089
scl00071.1_	Psmd13	yellow	19.918399
scl40888.19	Prkwnk4	yellow	19.823464

scl24866.28	Map3k6	yellow	19.740576
gi_3198188	B2m	yellow	19.61088
scl022192.1	Ube2m	yellow	19.467336
scl014319.1	Fth1	yellow	19.359767
scl27703.26	Pdgfra	yellow	19.222963
scl0001574.	G3bp	yellow	19.218687
scl36067.18	Aplp2	yellow	19.168153
scl36021.4.	Panx3	yellow	19.140315
GI_3808112	LOC386082	yellow	19.09454
scl053883.1	Celsr2	yellow	19.049347
scl44249.4_	AU040950	yellow	19.028814
GI_3808115	LOC386107	yellow	18.925622
scl0104318.	Csnk1d	yellow	18.828496
scl0012192.	Zfp36l1	yellow	18.790604
scl019173.2	Psmb5	yellow	18.749677
scl0017094	Erdr1	yellow	18.719963
ri|5430419F	5430419F0	yellow	18.706738
scl073274.2	1700034P1	yellow	18.464242
ri|3110001C	3110001O0	yellow	18.434665
scl46268.10	2610027L1	yellow	18.363221
GI_3808101	LOC386021	yellow	18.336231
scl0003645.	Slc30a5	yellow	18.140206
GI_3808114	LOC277837	yellow	18.111849
scl0066161.	Pop4	yellow	18.001403
scl35279.4.	Crtap	yellow	17.984484
scl20812.9.	Gorasp2	yellow	17.951505
scl0102462.	1190002L1	yellow	17.937763
scl0068186.	4632427E1	yellow	17.802799
scl0001641.	Slc29a1	yellow	17.789807
scl16583.17	Farslb	yellow	17.750358
scl28264.25	Eps8	yellow	17.705633
scl33753.14	Atp6v1b2	yellow	17.627206
ri|E030006M	4933437K1	yellow	17.621244
scl20251.8_	0610009I22	yellow	17.579704
scl0066789.	5430428G0	yellow	17.575748
ri|E430031D	E430031D1	yellow	17.543323
scl0022520	Zfp521	yellow	17.5383
scl36751.1.	4833444G1	yellow	17.483363
ri|6030461M	Csnk2a1-rs	yellow	17.461692
GI_3808133	LOC386246	yellow	17.461153
scl46882.12	5031439G0	yellow	17.343738
scl0067326.	1700037H0	yellow	17.251008
scl42522.11	Ifrd1	yellow	17.224057
scl15961.15	Uap1	yellow	16.923292
scl52308.5.	Csf2ra	yellow	16.567301
scl0001718.	Mylc2b	yellow	16.461301
scl015204.2	Herc2	yellow	16.455014
scl066151.5	1110020C1	yellow	16.40817
ri|1810032C	1810032O0	yellow	16.315118
scl0011983.	Atpif1	yellow	16.177048
scl43073.2_	Hspa2	yellow	16.060197
scl27067.26	Unc84a	yellow	16.05753
scl065115.4	Bean	yellow	15.983578
scl027050.3	Rps3	yellow	15.844097

scl43584.17	Slc30a5	yellow	15.843482
scl0074053.	Grip1	yellow	15.644222
GI_3808112	LOC386078	yellow	15.584512
GI_3808536	LOC381820	yellow	15.580827
scl41025.7_	AA959742	yellow	15.483912
scl39641.10	1300013D0!	yellow	15.480611
scl36916.10	Cspg4	yellow	15.461002
scl33485.7_	Herpud1	yellow	15.268119
GI_3808130	LOC386218	yellow	15.262424
scl0223920.	Soat2	yellow	15.191879
scl20804.11	Hat1	yellow	15.147264
scl0231997.	Fkbp14	yellow	15.145812
scl40135.12	Aldh3a2	yellow	15.112544
scl42730.18	2610204M0	yellow	14.816801
scl000628.1	Arl2bp	yellow	14.539462
scl22997.10	AI663987	yellow	14.536908
scl000377.1	Adk	yellow	14.467913
scl0110809.	Sfrs1	yellow	14.093392
scl020335.2	Sec61g	yellow	14.007113
scl35102.50	Col4a1	yellow	13.961607
scl18457.19	Trpc4ap	yellow	13.923415
scl27595.1.1	E330024J20	yellow	13.858798
scl26948.15	6330406I15	yellow	13.723614
scl0002818.	Rnf11	yellow	13.703573
scl0320357.	A330066M2	yellow	13.62979
scl075678.1	1810043M1	yellow	13.562609
scl46589.23	2310021P13	yellow	13.129808
ri|E430013O	E430013O1	yellow	13.116482
GI_3808725	LOC385505	yellow	12.960916
scl0004021.	Pi4k2b	yellow	12.941185
scl076014.3	5830416A07	yellow	12.751468
scl0110109.	Nol1	yellow	12.712965
gi_6671508_	Actb	yellow	12.507873
scl44517.9_	Lhfpl2	yellow	12.439639
scl000333.1	Tgfb1i4	yellow	12.36645
scl25944.17	Cyln2	yellow	12.277154
scl0067443.	Map1lc3b	yellow	12.230764
scl067604.1	1110007L15	yellow	12.205318
scl0330173.	2610524H06	yellow	12.203127
scl48193.7_	Dscr1	yellow	12.105578
scl0011491.	Adam17	yellow	12.041966
scl016452.2	Jak2	yellow	11.937859
scl0020877.	Sned1	yellow	11.800762
scl0218397.	Rasa1	yellow	11.784118
scl20384.11	Grp58	yellow	11.649148
scl19617.10	Pip5k2a	yellow	11.339391
scl24814.1.9	9130020K20	yellow	11.270777
scl0225876.	Fbxl11	yellow	11.190636
scl37434.1.1	9230105E05	yellow	11.100399
scl17359.5_	Rgs16	yellow	11.046404
scl34540.19	Vps35	yellow	10.780752
scl0094185.	Tnfrsf21	yellow	10.692483
scl058194.8	Sh3kbp1	yellow	10.62674
scl020535.2	Slc4a2	yellow	10.084665

scl20110.14	H13	yellow	9.9995525
ri|A630084D	A630084D0	yellow	9.6494981
GI_3808115	LOC386112	yellow	9.5924403
ri|6030455K	6030455K13	yellow	9.4522364
ri|9430068D	9430068D06	yellow	9.4193029
scl44750.12	2210404D11	yellow	9.3677619
scl0232431.	Rai3	yellow	9.2914831
scl0110196.	Fdps	yellow	9.1675924
ri|C920013G	C920013G1	yellow	9.0196192
scl29168.10	Slc35b4	yellow	8.9315214
ri|D030010H	D030010H0	yellow	8.8368463
scl35423.7.1	B130017P16	yellow	8.8318161
scl23210.3_	Foxo1	yellow	8.8159687
scl18934.1_	9930117H01	yellow	8.7305194
scl0023881.	E430034L04	yellow	8.4499075
scl0003519.1_175-S		yellow	8.3599778
scl020174.3	Ruvbl2	yellow	8.2802038
GI_3808127	LOC386192	yellow	8.0338256
scl0015365.1_6-S		yellow	7.9784256
scl0001243.1_522-S		yellow	7.9700822
scl026901.3	Deb1	yellow	7.9139573
scl31009.10	Dgat2	yellow	7.8895552
scl36497.2.1	C78915	yellow	7.8672806
scl011674.2	Aldoa	yellow	7.7937978
scl50948.6.8	Atp6v0e	yellow	7.684214
scl24876.1.4	2310005L22	yellow	7.6334998
scl36309.11	Abhd5	yellow	7.5074417
scl074479.1	Snx11	yellow	7.4674695
scl38511.3.1	Dcn	yellow	7.4109758
scl39403.17	Prkca	yellow	7.4018146
scl012505.1	Cd44	yellow	7.3932819
scl017313.2	Mglap	yellow	7.2707051
scl000857.1	Hsd11b1	yellow	7.2472208
scl075267.1	4930555L03	yellow	7.1079597
scl0016600.	Klf4	yellow	6.9276663
scl42955.2.1	Batf	yellow	6.847677
GI_3808498	LOC381801	yellow	6.5791932
scl015483.1	Hsd11b1	yellow	6.3776638
scl19891.17	Ddx27	yellow	6.0296252
ri|A730079J	Adss2	yellow	6.0047596
scl014104.1	Fasn	yellow	5.9947761
scl22702.29	Col11a1	yellow	5.871888
scl50988.5.3	0610007P22	yellow	5.8579266
scl38155.8_	Tnfaip3	yellow	5.8306736
scl0003598.1_52-S		yellow	5.7849861
scl26746.6.1	Cgref1	yellow	5.43425
scl00101502	Hsd3b7	yellow	5.3270576
scl39292.4_	Ptdsr	yellow	5.0278637
scl0076789.	2410129H14	yellow	5.0126457
scl25693.5.1	Chd7	yellow	4.7483127
scl23381.7.1	Car13	yellow	4.6942034
scl0002558.1_69-S		yellow	4.6604035
scl0078798.	Eml4	yellow	4.4888484
scl33916.7_	1810045K07	yellow	4.3679771

ri|E430033B	E430033B0	yellow	4.181152
scl054343.1	Atf7ip	yellow	4.0973227
scl19226.19	Rbms1	yellow	4.0560435
scl056876.1	Nelf	yellow	3.7851432
scl012608.3	Cebpb	yellow	3.6072462
scl0014284.	Fosl2	yellow	3.5594593
scl052120.1	D8Ertd354e	yellow	3.5466978
scl29857.13	D6Mm5e	yellow	2.7656735
scl21606.12	Slc35a3	green	71.908577
scl45275.6_	Akap11	green	70.316086
scl39900.2_	2810468K0	green	68.772743
scl24275.17	Rod1	green	68.620928
scl9889.1.1_	1110029L17	green	67.743412
scl5999.1.1_	2310014D1	green	67.401198
scl51275.14	Smad4	green	66.691075
scl31120.39	Iqgap1	green	65.806277
scl0002589.	Eef1d	green	65.127673
scl0319168.	Hist1h2ah	green	64.785745
scl0319173.	Hist1h2af	green	64.154092
scl0033852	A630082K2	green	63.083962
scl21081.7_	Tor1b	green	62.9218
scl0319170.	Hist1h2an	green	62.651359
scl0031917	Hist1h2ao	green	61.17806
scl48707.57	Pik4ca	green	60.605054
scl40651.35	4932417H0	green	60.408912
GI_3807828	LOC269529	green	59.528883
scl0227333.	Dgkd	green	58.634588
scl0319191.	Hist1h2ai	green	58.151391
scl072844.1	2900008M1	green	58.144397
scl19825.7_	Rab22a	green	57.848389
ri|2410042M	Dazap1	green	57.437925
scl26033.1.	9330180L1	green	57.103739
scl0031917	Hist2h2ac	green	56.931496
scl20027.27	Epb4.1l1	green	56.645694
scl0003566.	Tbrg1	green	56.513154
scl39740.21	Msi2h	green	56.256896
scl0077106.	5930418K1	green	56.156552
scl22501.2_	15-ก.ย.	green	56.007985
scl0226517.	9430023P1	green	55.854942
scl43443.14	Asxl2	green	55.07473
scl26247.31	Gak	green	54.974348
scl34984.1_	5830454D0	green	54.911126
scl53030.7_	D19Wsu162	green	54.600939
scl0010893	BC024659	green	54.567365
scl24926.6.	Ak2	green	54.419137
scl0140570.	Plxnb2	green	54.278442
ri|9430006C	9430006C2	green	53.858358
scl8585.1.1_	1110020K1	green	53.649324
scl0068045.	2700060E0	green	53.211519
scl35284.19	Pdcd6ip	green	53.155293
ri|D830018K	Mid2	green	52.83934
scl29566.11	Il17r	green	52.581153
scl6709.1.1_	Amotl1	green	52.403233
scl20455.7_	Spred1	green	51.962284

scl020462.9	Sfrs10	green	51.880773
scl32570.6.1	H47	green	51.577087
scl0211914.	Gm592	green	51.261905
scl000708.1	Atp6v0d1	green	50.555819
scl19300.3.1	2010311D03	green	49.425841
scl000993.1	Eef1b2	green	49.401225
scl069038.2	1810006K21	green	49.329611
scl0001867.	Sfrs10	green	49.173985
scl000981.1_40-S		green	49.063187
scl20797.28	Itga6	green	49.022782
scl32148.1.3	3830612M24	green	48.861646
scl0003150.	Golga2	green	48.733297
ri|1110018N	1110018N24	green	48.659432
scl059042.1	Cope	green	48.566296
scl0330286.	D630045J12	green	48.460887
ri|E430002N	E430002N0	green	48.407369
ri|E430005I(	E430005I09	green	48.077799
GI_3006139	Hist1h2ai	green	47.900863
scl28753.6_	Pcyox1	green	47.826168
scl066480.2	Rpl15	green	47.793961
GI_3006137	Hist1h2ak	green	47.769612
scl27274.14	Brap	green	47.528804
GI_3006132	Hist1h2ah	green	47.474627
scl32175.1_	2610024B07	green	47.411973
scl43285.27	Snx13	green	47.1554
scl16709.1.5	9430025M2	green	47.137887
scl012819.4	Col15a1	green	47.09341
scl15945.4.1	1110021H02	green	46.949555
scl37761.6_	Ppap2c	green	46.397469
ri|3110001P	3110001P07	green	46.161347
scl37797.29	Col6a2	green	46.103407
scl22810.11	Igsf3	green	45.370223
scl52517.51	Fer1l3	green	45.33724
scl37903.28	Hk1	green	45.295561
scl50680.31	Xpo5	green	44.927724
scl15963.1.2	LOC98434	green	44.89496
scl44772.3.1	Gadd45g	green	44.755499
scl052713.2	D10Ertd718	green	44.730587
scl37933.6_	D10Ertd641	green	44.641787
scl069985.1	Sox12	green	44.613241
scl5074.1.1_	Freq	green	44.411209
scl50935.5_	Hmga1	green	44.14908
GI_3808016	Usp7	green	43.705809
scl0001708.1_442-S		green	43.687586
GI_3006136	Hist1h2ag	green	43.480951
scl0014664.	Slc6a9	green	42.578583
scl0002708.	0610037L13	green	42.481279
scl070544.3	5730437N04	green	41.986789
scl0067495.	2010200O10	green	41.824788
scl25416.13	Rad23b	green	41.629041
GI_2082516	AJ237586	green	41.487127
scl25019.5.1	Edn2	green	41.354877
ri|5730552M	5730552M2	green	41.296779
scl37065.9_	9030425E11	green	41.027518

scl0002880.	Emd	green	40.90116
scl31690.17	Vasp	green	40.5471
scl0013629.	Eef2	green	40.181145
scl21748.12	Vangl1	green	40.135721
ri|C730016M	C730016M0	green	40.059766
scl0002617.1_582-S		green	39.911239
scl000872.1	2510010F15	green	39.904222
GI_3808999	1190002N15	green	39.812019
scl52981.3.1	Adra2a	green	39.454534
scl020104.1	Rps6	green	39.377622
scl0066853.	Pnpla2	green	38.926899
ri|C730026C	C730026O1	green	38.598574
scl0020856.	Stc2	green	38.357261
scl014312.1	Brd2	green	38.040145
scl32503.17	Agc1	green	37.968471
scl0001607.	2810410M20	green	37.84822
scl0002016.	D3Ertd194e	green	37.746602
ri|A630046C	A630046C1	green	37.591616
scl0020848.	Stat3	green	37.493345
scl25131.24	Eps15	green	37.277675
scl074780.1	Glce	green	37.263454
scl33862.17	Fath	green	37.22593
scl34321.28	Glg1	green	36.786087
scl27015.4.1	Kdelr2	green	36.641867
ri|2010007E	2010007E07	green	36.599577
scl068750.4	1110037N09	green	36.123001
scl0017938.	Naca	green	36.035236
scl068494.1	1110011C06	green	35.976872
scl49941.4.1	Znrd1	green	35.438224
ri|C330049H	C330049H0	green	35.22371
scl34330.23	Sf3b3	green	34.906601
scl34210.5.1	3010027A04	green	34.684318
scl29998.16	5830411G16	green	34.665718
scl26054.30	Rsn	green	34.372401
scl0003257.	mSMO	green	34.346834
scl0001372.	Clk4	green	34.120848
scl0022218.	Sumo1	green	34.113784
GI_7709985	Uble1b	green	33.966309
scl0327932.	G3bp	green	33.941162
scl022381.3	Wbp5	green	33.888133
scl38398.1.9	1110060I01	green	33.809807
scl54847.11	Slc6a8	green	33.588054
scl42759.15	D12Wsu95e	green	33.47909
scl0004020.1_31-S		green	33.342148
scl066840.3	0610008N23	green	33.326192
GI_3198099	Prss25	green	33.307968
scl41885.21	1700011I11	green	33.204754
scl37767.8_	1200008N06	green	33.042497
scl28755.11	Dusp11	green	33.025332
scl066272.3	1810020G14	green	32.91371
scl0004156.	1600019D15	green	32.661576
scl48035.1.1	B230362B09	green	32.429807
scl013629.6	Eef2	green	32.354236
scl0023234.1	Prkwnk1	green	31.833052

scl49877.10	Vegfa	green	31.442656
GI_3809134	A230103N1	green	31.420326
scl17048.2_	AW112037	green	31.311952
scl000989.1	sty	green	30.946174
scl34415.5.1	Rrad	green	30.84428
scl0072102.	Dusp11	green	29.97397
scl0383295.	Ypel5	green	29.973055
scl54837.5.1	Emd	green	29.87616
GI_3807865	LOC230628	green	29.672612
scl0019349.	Rab7	green	29.653536
scl0050771.	Atp9b	green	29.442575
scl39409.1_	AI875142	green	29.435938
scl0031948(	Itga11	green	29.377192
scl42223.4_	1110014C0	green	29.264167
ri|9130201F	9130201F2	green	29.257796
GI_3212922	Zfp533	green	29.170816
scl23509.31	Ube4b	green	28.952445
scl16508.16	Ngef	green	28.912579
scl00002.1_	2610024E2	green	28.820062
scl0079202.	Tnfrsf22	green	28.780243
scl16982.5.1	Tram1	green	28.749151
scl0108123.	Napg	green	28.728701
scl30408.3.3	Tac1	green	28.107433
scl15987.1.1	AI481316	green	28.090856
scl37040.9_	Dpagt1	green	28.005701
GI_3807623	LOC381439	green	27.98421
scl18325.32	3632413B0	green	27.914472
scl39642.11	Pip5k2b	green	27.855754
scl15959.1_	4732477C1	green	27.411775
scl19763.10	Dnajc5	green	27.087105
ri|6330545A	Anxa7	green	27.068634
scl0002699.	Nol6	green	27.004731
scl00026.1_	H47	green	26.941077
scl27228.32	Hip1r	green	26.908484
scl0213491.	D4Ertd22e	green	26.898406
scl41159.3.1	Ccl7	green	26.824911
scl45943.26	Kpnb3	green	26.652386
scl022333.1	Vdac1	green	26.266911
scl00018.1_	Ptpre	green	26.241319
GI_3006137	Hist1h2ao	green	26.179715
scl44416.3_	2810008M2	green	26.03207
scl24398.4.1	Hint2	green	25.83146
scl33186.17	Galnt2	green	25.821078
scl50767.4_	Nrm	green	25.797222
scl36472.2.2	Gpx1	green	25.747739
scl34420.4.1	Cklfsf4	green	25.662952
scl0002449.1_77-S		green	25.514528
scl25041.1.4	Elovl1	green	25.411013
scl23794.11	BC039093	green	25.395233
scl0066212.	Sec61b	green	25.151205
GI_3006139	Hist2h2aa2	green	25.096525
scl014683.1	Gnas	green	25.084475
scl0012861.	Cox6a1	green	24.78999
scl012861.2	Cox6a1	green	24.717024

scl25023.8_	Hivep3	green	24.612491
ri|9830143E	9830143E02	green	24.489744
scl020848.1	Stat3	green	24.485021
scl46964.12	4732495E13	green	24.392123
scl0020698.	Sphk1	green	24.340031
ri|2400007G	2400007G07	green	24.034322
scl29051.11	Cai	green	23.925527
scl020868.2	Stk10	green	23.831436
scl067785.1	Zfp262	green	23.747923
scl21676.20	6330569M2	green	23.717733
ri|D030074C	Dtnb	green	23.550439
scl44493.21	Col4a3bp	green	23.246267
GI_3807958	LOC384161	green	23.049402
ri|6430540M	6430540M20	green	22.763596
scl067876.4	1500041J02	green	22.758691
scl50143.3.1	0610039D01	green	22.708191
GI_3808180	4930432O2	green	22.360151
ri|9230102G	9230102G00	green	22.2155
scl33177.8_	Tsnax	green	22.195852
scl0067949.	Mki67ip	green	21.69168
ri|1200006J	Slc21a2	green	21.577506
scl0002494.	Sqle	green	21.347519
scl012417.4	Cbx3	green	21.298122
scl46260.8_	9130227C08	green	20.841116
scl20442.1.1	5430417L22	green	20.21091
scl31989.25	Tacc2	green	20.146328
scl014828.8	Hspa5	green	20.101507
scl022294.1	Uxt	green	19.910473
scl54000.2.1	2700099C19	green	19.879362
GI_3006135	Hist1h2ad	green	19.852545
ri|A230055C	A230055O0	green	19.835103
scl0001062.	Cnot4	green	19.551011
scl019653.6	Rbm4	green	19.193676
scl000911.1_939-S		green	19.063145
ri|5730441M	5730441M1	green	19.052379
scl33270.1.1	A130010C1	green	19.018579
GI_3807610	LOC382885	green	18.801547
scl0028042.	D5Wsu178e	green	18.756685
scl016477.1	Junb	green	18.737364
scl0058172.	Sertad2	green	18.731561
scl17154.1.4	A730054J21	green	18.490869
scl030056.1	Timm10	green	18.477227
scl18949.1_	Fjx1	green	18.470444
GI_3198194	Commd3	green	18.389046
scl0003638.	2310016C16	green	18.379925
scl0004190.1_3-S		green	18.24151
scl23964.7.1	2810405F18	green	18.213145
scl33327.2_	BC025546	green	18.113047
scl0021786	Eif5	green	18.075317
scl42112.9.1	Ddx24	green	17.886238
scl066270.6	1810015C04	green	17.870715
scl36396.16	Ubp1	green	17.814834
scl32713.7.1	1110007C05	green	17.665927
scl42756.16	Traf3	green	17.635139

scl0012848.	Cops2	green	17.362923
scl41462.6_	2310040C0!	green	17.233164
scl54641.8.!	Srpx2	green	17.22235
scl17695.4.:	Neu2	green	17.043981
scl017846.2	Commd1	green	16.888365
scl18201.5_	Btbd4	green	16.819498
scl066583.1	Exosc1	green	16.689936
scl0069116.	1810009A1(	green	16.648869
scl48775.5_	Emp2	green	16.496535
scl0069188.	Mll5	green	16.489867
scl0010867:	D19Ertd678	green	16.444622
scl068842.1	Tulp4	green	16.439161
scl41379.14	Aloxe3	green	16.300795
scl44463.11	Smn1	green	16.280729
scl0218885.	BC019806	green	16.241845
scl20689.4.:	Timm13a	green	16.214867
scl0066827.	Ttc1	green	15.832641
scl50235.5.:	Tnfrsf12a	green	15.790939
scl36121.3_	1110011K1(	green	15.493294
scl057295.5	Icmt	green	15.489657
scl0215015.	C530043G2	green	15.29938
scl54919.12	Fhl1	green	15.230552
scl33694.4.:	Pgls	green	15.225402
scl019933.5	Rpl21	green	15.131428
scl0001329.	0610009H0⁄	green	14.936738
scl41358.1_	1810027O1(	green	14.670375
scl0104112.	Acly	green	14.640815
scl44474.1.7	1700054E1:	green	14.520244
scl0015267.	Hist2h2aa1	green	14.499723
scl067337.2	Cstf1	green	14.485322
scl0192170.	Ddx48	green	14.423708
scl0230514.	Obrgrp	green	14.419298
scl17725.6_	Itm2c	green	14.164502
scl47630.6_	Pim3	green	13.937517
scl0067231.	2810442O1(	green	13.866876
scl027407.6	Abcf2	green	13.77476
ri|2500004H	2500004H2:	green	13.722472
scl0319475.	4930488P0(	green	13.692703
scl0003895.	Myl6	green	13.418375
GI_3809670	LOC331102	green	13.418359
scl071175.1	4933421G1!	green	13.053117
GI_3809033	LOC270157	green	13.047054
scl15852.36	Elys	green	12.914464
scl0014621.	Gjb4	green	12.896043
scl35671.6_	Lactb	green	12.496726
scl40975.9.:	Copz2	green	12.219602
scl18219.6_	Ythdf1	green	12.107315
scl0021360.	Targ1	green	12.060074
scl22902.7.:	Mrpl9	green	12.041855
scl0030046.	Zfp292	green	12.040168
scl23735.14	Chc1	green	12.036365
scl40311.8.:	5730409G0	green	11.921054
scl068512.1	1110019J04	green	11.804685
scl41866.19	Aebp1	green	11.797728

scl0016796.	Lasp1	green	11.695468
scl37011.7_	Fxyd6	green	11.612714
scl29414.11	Strap	green	11.590059
scl22692.12	Dbt	green	11.44049
scl0031987(	Cobll1	green	11.384025
scl26655.5_	Hs3st1	green	11.151155
scl0001438.	Copz2	green	11.062705
scl26144.4_	Hspb8	green	10.965308
scl066917.1	Chordc1	green	10.802833
ri|B230363H	B230363H0	green	10.610835
scl065972.3	Ifi30	green	10.561833
ri|9430065L	9430065L19	green	10.325019
scl0214290.	Zcchc6	green	10.253331
scl38195.4_	Stx11	green	10.210611
scl0002644.1_1164-S		green	10.132324
scl0003623.	Pfkp	green	10.096423
scl24643.7_	2900010D03	green	9.9001641
scl23946.8.1	Urod	green	9.8811324
scl26445.6.1	Igfbp7	green	9.8655209
ri|5830407L	Uqcrb	green	9.8153215
scl52843.9.1	Efemp2	green	9.6139234
scl0001472.	Copz2	green	9.4603388
scl067165.1	2610204B21	green	9.3702287
scl26399.6.8	BC038311	green	9.2800604
ri|A430053B	Cul1	green	9.1194507
scl54548.7.1	Hadh2	green	9.0662484
scl13778.1.1	Ptp4a1	green	8.5029953
scl41822.1.1	9030024J15	green	8.3643184
scl019720.8	Trim27	green	8.3318198
scl24984.5.1	BC023823	green	8.2964654
scl24750.20	Epha2	green	7.9684974
scl068944.7	4930403O06	green	7.7672234
scl0246277.	Csad	green	7.7542654
scl33969.1.9	D030041N0	green	7.5836847
scl0268490.	2600001B17	green	7.4486639
scl47621.3.1	Adm2	green	7.3771919
scl23453.4_	1200015A19	green	7.3383242
scl52445.7_	Scd1	green	7.3278423
scl40538.6.1	H2afv	green	7.0458751
scl011754.4	Aoc3	green	6.5895407
ri|0610040O	0610040O15	green	6.5769221
scl43551.17	Nln	green	6.5412788
scl24041.13	Pcsk9	green	6.2747966
scl0327759.	Unc5b	green	6.2693176
scl47757.5.8	Lgals1	green	5.9591791
ri|D130064I(	Hmg20a	green	5.8502546
scl0018708.	Pik3r1	green	5.8169437
scl39516.6_	2600001B17	green	5.7886663
scl49445.4_	1810013L24	green	5.6442807
scl021937.1	Tnfrsf1a	green	5.5173918
scl38666.13	Thop1	green	5.5034847
scl46602.21	Nid2	green	5.492146
scl0380921.	Dgkh	green	5.4441964
GI_3808103	A730011F23	green	5.4164762

scl074170.1	1810018P12	green	5.3746901
scl016432.2	Itm2b	green	5.2988912
scl22405.4_	Hey1	green	5.1617256
GI_2848867	LOC330844	green	5.120677
scl41049.3_	1810057C19	green	5.0191058
scl071704.9	Arhgef3	green	4.9092461
scl47731.8_	AI452372	green	4.8928098
scl17432.6_	Csrp1	green	4.7628748
scl012331.2	Cap1	green	4.6679
scl31677.12	Relb	green	4.5681208
scl30008.5_	Aqp1	green	4.5248538
scl44104.6.1	1300014I06	green	4.5141377
scl051795.1	Srpx	green	4.4950484
scl24858.5.1	AI838661	green	4.3016376
scl000936.1_18-S		green	4.2236068
scl0230678.	6330530A05	green	4.0763043
scl35175.1.1	2310037P21	green	3.9171272
scl074761.1	1200013A08	green	3.537969
scl026557.1	Homer2	green	3.3818148
scl517.1.1_2	2610001E17	green	3.3260654
scl0012394.	Runx1	green	3.3171026
scl020567.3	Slp	green	3.2274947
scl25176.1.2	Dhcr24	green	3.1529912
GI_3432817	Fv1	green	2.4635517
scl38890.17	P4ha1	green	1.5383412
scl32896.3_	Sertad1	green	1.4283572
ri|6030499A	6030499A19	red	32.686558
scl0002975.1_346-S		red	31.172715
GI_3807643	LOC382895	red	28.935357
scl0056304.	LOC56304	red	28.906719
scl44975.2_	Aldh5a1	red	27.825738
GI_2847699	LOC329092	red	27.682673
scl0014584.	Gfpt2	red	27.603636
ri|C130099E	C130099E04	red	27.206984
scl0001849.1_2273-S		red	27.031822
scl39016.40	Lama4	red	26.869836
scl0077302.	9430078K10	red	26.775221
scl21989.6_	BC023814	red	26.694198
scl37744.8.1	ORF61	red	25.807929
scl31730.8.4	Sepw1	red	25.673876
scl18990.34	Dgkz	red	25.075617
scl012847.1	Copa	red	25.050111
ri|4732468I0	Pla2g1br	red	24.242849
scl47884.11	Sqle	red	24.123734
scl067959.1	2410104I19	red	23.942244
ri|4832436M	4832436M0	red	23.876624
scl4354.1.1_	Olfr1006	red	23.795726
scl48642.15	Etv5	red	23.532205
scl52703.1_	D930033H1	red	23.445962
scl0003999.	Plod3	red	23.343194
ri|9030404K	Zfp207	red	23.152998
scl50509.4_	Lbh	red	23.101835
scl0217039.	Ggnbp2	red	23.089256
scl46547.15	Anxa11	red	22.94169

ri|D130074M	D130074M1	red	22.816634
scl0320292.	Rasgef1b	red	22.700739
scl30685.21	Atxnl2	red	22.173701
scl40837.7_	Arf2	red	22.153728
ri|5330440C	Ccnd2	red	22.046653
ri|9630027A	9630027A13	red	21.749528
scl022130.1	Ttf1	red	21.597592
scl017356.2	Mllt4	red	21.581895
scl00239318	Plcxd3	red	21.159946
GI_6200068	Shf	red	20.997972
scl076799.5	2510006D16	red	20.889525
scl49148.7.8	Cd80	red	20.848869
scl0013135.	Dad1	red	20.846275
scl0002999.	Tgm2	red	20.585826
ri|5730406C	5730406O18	red	20.475215
ri|0610042I0	Rmrp1	red	20.441297
scl013717.1	Eln	red	20.310094
GI_1094663	Rangnrf	red	20.228847
scl0076650.	Npn3	red	20.12233
scl33015.27	Sympk	red	19.958365
ri|9030619K	9030619K07	red	19.894946
scl00223658	D330001F17	red	19.712803
scl43064.12	Fntb	red	19.695954
GI_3808921	Fath	red	19.18455
scl0076695.	2010321I05	red	18.908766
ri|A230024N	A230024N0	red	18.839469
scl076900.1	Ssbp4	red	18.673551
scl0208715.	Hmgcs1	red	18.482455
scl0002810.	Mknk1	red	18.389009
scl0078908.	Igsf3	red	18.190406
scl016800.2	Arhgef2	red	17.967351
ri|9930033H	9930033H14	red	17.751024
scl0019027.	Sypl	red	17.621935
GI_3808499	Plxnd1	red	17.383306
scl49993.30	Bat2	red	17.363316
GI_3808125	LOC386169	red	17.018968
scl32363.39	Odz4	red	16.907071
scl39495.10	Gfap	red	16.455435
scl18005.4.1	1500015O10	red	16.397825
scl012877.1	Cpeb1	red	16.308436
scl20106.5.1	Cox4i2	red	16.210018
ri|B930092H	Nfib	red	16.01608
scl000639.1	1110017C15	red	15.980726
scl019989.6	Rpl7	red	15.616084
scl24885.8.1	Matn1	red	15.525675
scl00319622	.1_241-S	red	15.424147
scl47395.9.8	1110064N10	red	14.819978
scl37752.7.6	Rnf126	red	14.768964
scl30826.9_	D7Ertd743e	red	14.487834
ri|9430039I1	Nfib	red	14.401504
GI_3323941	A230072I16	red	14.378504
scl46658.31	Itga5	red	14.208591
scl48911.16	Usp16	red	14.124803
scl34640.17	Large	red	14.072404

scl0001118.1_0-S		red	13.78218
scl0066511.	2500003M1(	red	13.479935
ri|C330007M	Chd1	red	13.393636
ri|D030044M	D030044M2	red	13.304785
scl00108989	Tpr	red	13.220396
scl31635.5.1	Pafah1b3	red	13.195997
ri|A530065H	A530065H2	red	12.991872
scl18703.6_	2810405K07	red	12.985455
scl23744.24	Epb4.1	red	12.974924
scl0072141.	Adpgk	red	12.922804
scl16375.7_	Dbi	red	12.916696
scl28535.9.1	Sec13l1	red	12.916409
scl018194.8	Nsdhl	red	12.888156
scl0067921.	2510010F15	red	12.861364
scl0019822.	Rnf4	red	12.621672
scl066364.3	2310009A05	red	12.620726
scl069435.3	1700023M0	red	12.554094
ri|0710007C	Mrpl15	red	12.446905
scl0019291.	Purb	red	12.443633
scl067148.6	2610204K14	red	12.064732
GI_3807436	LOC329750	red	11.876218
scl47761.2_	Pdxp	red	11.864737
ri|9530086N	Lbcl1	red	11.690767
ri|F630118K	Traf1	red	11.485895
scl021969.2	Top1	red	11.25986
ri|D130083G	D130083G0	red	11.233021
scl17458.10	Chi3l1	red	11.115951
ri|9530058N	Ril-pending	red	11.002624
scl056264.2	Cpxm1	red	10.834697
scl26985.7_	2700038I16	red	10.593723
ri|6030458C	6030458C11	red	10.468768
ri|2510002P	2510002P07	red	10.452286
scl0002413.	Hbp1	red	10.347977
GI_3807962	LOC231081	red	10.225974
scl018412.1	Sqstm1	red	10.162717
GI_3808022	LOC385699	red	10.011366
scl33423.6_	Cbfb	red	9.8879826
GI_2089255	Atp6a1	red	9.8599196
scl0003277.	H13	red	9.7820363
scl23887.17	Ctps	red	9.7388511
scl31800.2.1	2210411K11	red	9.6189846
scl0021983.	Tpbg	red	9.5975214
GI_3808808	LOC381951	red	9.5371179
scl0380601.	C78212	red	9.446125
scl34760.11	Cpe	red	9.3107583
scl020810.7	Srm	red	9.2584538
scl017169.9	Mark3	red	9.2336327
scl0023802.	Amfr	red	9.1532681
scl0014325.	Ftl1	red	9.0743564
scl0330636.	C130081G2	red	8.921008
scl43022.7_	4933426M1	red	8.9203206
scl24244.27	Tnc	red	8.9191281
scl0107094.	AA408556	red	8.9187754
scl42425.21	Sec23a	red	8.8502728

scl27580.3_	D5Ertd593e	red	8.8175371
scl46905.12	Dia1	red	8.699326
scl0018186.	Nrp	red	7.984953
scl075956.6	Srrm2	red	7.9580877
scl26181.16	Trpv4	red	7.9028255
scl069570.2	2310024N1	red	7.8765887
scl067704.2	1810037I17	red	7.7888571
scl42553.13	Hbp1	red	7.682748
scl29552.17	Slc6a12	red	7.6534238
scl25354.23	Pappa	red	7.6383589
GI_3807964	Whsc1l	red	7.6106607
scl068947.1	Chst8	red	7.5554134
GI_3808646	LOC382230	red	7.4760847
scl53173.13	E430027O2	red	7.4611255
scl0002080.	15-ก.ย.	red	7.3986597
scl41352.8_	Dullard	red	7.1617975
scl30453.23	Cars	red	7.158232
scl19480.4_	Zdhhc12	red	7.1417169
scl014470.4	Rabac1	red	7.1230585
scl015381.1	Hnrpc	red	7.0918584
scl0108888.	Atad3a	red	6.9515113
scl46766.10	Ccnt1	red	6.8660461
scl0002716.	Scp2	red	6.8240842
scl0320368.	A730063M1	red	6.8203233
scl29667.6_	Bhlhb2	red	6.753889
ri|D930042A	D930042A2	red	6.7014002
scl0016527.	Kcnk3	red	6.6314484
scl37715.13	Lmnb2	red	6.6141325
scl21831.10	Ecm1	red	6.5180464
scl19917.13	Mmp9	red	6.5132912
scl29233.13	Wasl	red	6.4700861
scl071514.8	Sfpq	red	6.4612426
scl51713.6.3	Cyb5	red	6.4536113
scl0021623	Socs2	red	6.4437943
mtDNA_ND5-S		red	6.3693667
scl0017045	Stard4	red	6.2534222
scl22859.6.1	D130027M0	red	6.2363311
scl24151.12	Adfp	red	6.225537
scl43441.25	Dtnb	red	6.1930753
ri|2610511L	Ddx21	red	6.1748561
scl015191.7	Hdgf	red	6.1344609
scl36459.1.1	D630038D1	red	6.0486216
GI_3808382	LOC225456	red	6.0189278
scl50138.8_	Lemd2	red	5.9694132
ri|F830002E	F830002E1	red	5.8495951
ri|2700079K	2700079K0	red	5.7689931
GI_3808073	LOC385825	red	5.7237385
GI_3808918	Mfhas1	red	5.7217887
scl40036.2_	2600017H0	red	5.6797695
scl39318.8_	Wbp2	red	5.623517
scl50087.9.2	Glo1	red	5.6141815
GI_3807642	LOC380927	red	5.5730504
scl52451.21	Chuk	red	5.5275064
scl47036.7.1	Vps28	red	5.346232

scl20303.17	Rpo1-2	red	5.275002
ri|2310045K	2310045K21	red	5.207964
scl38332.1_	A730063M1	red	5.16183
scl20013.3_	Manbal	red	5.0687703
ri|6430598P	Mad5	red	5.0291472
scl0017721.	mt-Nd5	red	4.8710558
scl0014455.	Gas5	red	4.8003903
scl0053379.	Hnrpa2b1	red	4.6501753
scl18785.23	A930025J12	red	4.5784969
scl47441.30	Hemp1	red	4.4781608
scl22993.15	2810403A07	red	4.4688375
scl019042.1	Ppm1a	red	4.4401694
scl0010436	Rnu65	red	4.3161638
scl33659.17	Mcm5	red	4.2966269
scl0015081.	H3f3b	red	4.0795824
scl068180.6	6430559E15	red	4.0369594
scl51530.6.1	5133400G04	red	4.006857
scl0023556	Dnajc13	red	3.9806459
scl0010031	AU040320	red	3.9709176
scl0003181.	Fkbp1a	red	3.9697241
GI_3809158	LOC268393	red	3.9631807
scl000898.1	Ivns1abp	red	3.8984556
scl44577.6.1	Cryba4	red	3.8551055
scl0031936	C920004C0	red	3.7744275
scl34209.1_	3010027A04	red	3.7552478
scl0001044.	Arl6ip5	red	3.6400444
scl23678.5.1	3200001F09	red	3.6089202
scl0002504.	Atf4	red	3.5544973
scl30741.3_	Thumpd1	red	3.5338823
scl46654.8.1	Ppp1r1a	red	3.5110616
scl0330481.	1190028F09	red	3.4857895
scl30062.4.1	Npy	red	3.397821
scl31678.22	Sfrs16	red	3.3643297
ri|2010004G	Fibp	red	3.2189305
scl49375.21	Lztr1	red	3.1512666
scl030057.2	Timm8b	red	3.0922416
scl38444.3.1	Phlda1	red	2.9419662
GI_3808145	LOC280487	red	2.8975995
ri|2900060F	2900060F21	red	2.8954562
scl27722.12	Asrij	red	2.8104767
scl015469.1	Hrmt1l2	red	2.711429
scl094064.4	Mrpl27	red	2.7018246
scl38626.3.1	Chst11	red	2.6633517
scl36650.13	Bckdhb	red	2.6135567
scl0010900	Ciapin1	red	2.4033036
scl000591.1	Ifi30	red	2.2127563
scl16395.1.2	2900060B14	red	1.9742448
GI_3808320	LOC381747	red	1.9080047
GI_3808135	LOC381683	red	1.6870425
scl017992.3	Ndufa4	greenyellow	60.217497
scl0001934.	Lmna	greenyellow	57.664043
scl37396.13	D10Ertd610	greenyellow	55.642756
scl0245877.	BC019977	greenyellow	55.560437
scl016905.3	Lmna	greenyellow	55.268094

scl0022379.	Fmnl3	greenyellow	55.178124
scl49943.4_	Ppp1r11	greenyellow	54.806137
scl023825.3	Banf1	greenyellow	51.975079
scl33261.5_	Hsbp1	greenyellow	51.249625
GI_5803720	Bag5	greenyellow	51.053701
scl34310.8_	Bcar1	greenyellow	50.833429
scl0016905.	Lmna	greenyellow	50.661211
scl21973.8_	Lmna	greenyellow	49.666859
scl066660.3	5730555F13	greenyellow	49.627634
scl073736.7	1110008B24	greenyellow	49.615673
scl056347.1	Eif3s8	greenyellow	49.38129
scl42528.12	Bzw2	greenyellow	49.073715
scl0069994.	1700027M0	greenyellow	48.963619
scl000575.1	Sh3md2	greenyellow	48.772516
GI_3808955	LOC234582	greenyellow	48.629365
scl073737.1	1110008P14	greenyellow	47.649974
scl46899.17	Arfgap3	greenyellow	47.256173
scl0026959	Luzp1	greenyellow	46.424186
scl30470.4_	H19	greenyellow	46.407618
scl34083.48	Col4a2	greenyellow	45.963416
scl35708.10	Smad3	greenyellow	45.525729
ri|A530030G	A530030G1	greenyellow	45.51825
scl0027373.	Csnk1e	greenyellow	45.50575
scl0223649.	BC011468	greenyellow	44.906338
scl37311.9.1	4833420K19	greenyellow	44.578037
scl33522.11	Chd9	greenyellow	44.564868
scl40658.10	D11Ertd759	greenyellow	44.055265
scl31343.5.1	Saa3	greenyellow	44.004544
GI_3807344	LOC381297	greenyellow	43.806201
scl0050907.	Preb	greenyellow	43.45516
ri|B130038H	1110001N0	greenyellow	43.237726
scl0021575	BC013529	greenyellow	43.210362
scl24957.6.1	Psmb2	greenyellow	43.166299
scl000538.1	Banf1	greenyellow	43.0351
scl0216874.	Camta2	greenyellow	42.930729
scl068544.3	2310036O2	greenyellow	42.621854
scl18413.5.1	9430008C0	greenyellow	42.534655
scl013807.1	Eno2	greenyellow	41.917515
scl011951.2	Atp5g1	greenyellow	41.479027
scl0002680.	Nsep1	greenyellow	41.195714
scl0320706.	9830001H0	greenyellow	41.023888
scl36290.7_	Limd1	greenyellow	41.014255
scl050918.1	Myadm	greenyellow	40.697703
scl0022325	Farp1	greenyellow	39.740733
scl43963.10	Ror2	greenyellow	39.463501
scl42739.19	Kns2	greenyellow	39.461953
scl013204.1	Dhx15	greenyellow	39.389343
scl47449.2_	Hoxc8	greenyellow	39.188119
scl0098732.	1110059F07	greenyellow	38.925715
scl018477.6	Prdx1	greenyellow	38.580449
scl0002929.	2610029G2	greenyellow	38.499336
scl0018789.	Papola	greenyellow	38.423799
scl44054.10	Nedd9	greenyellow	38.281708
scl0021453.	Tcof1	greenyellow	38.034558

ri|1110001N	1110001N0(	greenyellow	37.888487
scl40209.28	Anxa6	greenyellow	37.817706
scl0001433.	Psmc5	greenyellow	37.811706
scl39282.4_	Socs3	greenyellow	37.806378
scl39517.28	Hdac5	greenyellow	37.696098
scl026446.4	Psmb3	greenyellow	37.694337
scl22853.10	Itga10	greenyellow	37.560876
scl0116701.	Fgfrl1	greenyellow	37.441479
scl0004181.	Vps29	greenyellow	37.01933
scl49960.7_	Trim39	greenyellow	36.912389
scl53012.17	AI450540	greenyellow	36.787166
scl33707.42	Myo9b	greenyellow	36.42855
scl23190.10	Spg20	greenyellow	36.094875
scl45531.6.1	Nedd8	greenyellow	36.088276
scl0011988.	Slc7a2	greenyellow	35.853084
scl40910.10	Fkbp10	greenyellow	35.718789
ri|2310008I2	2310008I22	greenyellow	35.645479
scl34704.4_	C630013N1	greenyellow	35.378154
scl35482.3.1	Ssb4	greenyellow	34.643251
scl22947.3.8	S100a13	greenyellow	34.447943
scl24865.12	Slc9a1	greenyellow	34.232068
scl0002044.	Alg5	greenyellow	33.69289
scl067605.5	Akt1s1	greenyellow	33.11684
scl0003111.	Pfkfb3	greenyellow	32.960694
scl0056438.	Rbx1	greenyellow	32.802536
ri|E030040G	E030040G2	greenyellow	32.541781
scl0066989.	2410004N1	greenyellow	32.100064
scl30359.11	Tes	greenyellow	31.944296
scl0320365.	9330186A19	greenyellow	31.824761
scl099982.1	Aof2	greenyellow	31.824168
scl071228.1	Dlg5	greenyellow	31.711667
scl022038.3	Plscr1	greenyellow	31.500702
scl29294.14	Asns	greenyellow	31.413282
scl49001.3_	Cggbp1	greenyellow	30.923144
scl0017925.	Myo9b	greenyellow	30.702248
ri|4632409D	Col12a1	greenyellow	30.230605
mtDNA_ND2-S		greenyellow	30.203309
scl42789.1.1	Gtl2	greenyellow	30.134285
scl0056458.	Foxo1	greenyellow	29.987064
scl0016949.	Loxl1	greenyellow	29.160301
scl25158.13	Glis1	greenyellow	29.108381
scl072043.2	Sulf2	greenyellow	28.412856
scl075710.1	Rbm12	greenyellow	28.077951
scl0003661.	Nedd9	greenyellow	27.953405
scl0021808.	Tgfb2	greenyellow	27.884014
scl029876.1	Clic4	greenyellow	27.834512
scl0018573.	Pde1a	greenyellow	27.549237
scl43921.12	Pdlim7	greenyellow	27.520168
scl34345.7_	Hp	greenyellow	27.323988
scl0067684.	3300001P08	greenyellow	27.10669
scl20687.14	Slc43a3	greenyellow	26.576769
scl0215436.	Slc35e3	greenyellow	25.58486
scl0021681.	Thoc4	greenyellow	24.313214
scl0003537.	Pcolce2	greenyellow	24.16601

scl52550.23	Tmem23	greenyellow	23.969401
scl021371.3	Tbca	greenyellow	23.379514
scl31557.2_	Kcnk6	greenyellow	21.995736
scl0003990.	Hip2	greenyellow	21.908743
scl33493.3.1	Mt2	greenyellow	20.529208
scl570.1.1_2	1600021P15	greenyellow	18.925143
scl075977.2	5031425E22	greenyellow	18.878643
scl29649.6.1	Lmcd1	magenta	34.257993
scl0320770.	A630072M1	magenta	33.779989
scl013132.1	Dab2	magenta	32.303463
scl24190.1_	E030026I10	magenta	31.922791
scl0016987.	Lss	magenta	31.162319
scl054196.5	Pabpn1	magenta	30.877756
scl47431.10	AW549877	magenta	30.358037
scl42938.12	Gstz1	magenta	30.299257
scl23489.7_	4930422J18	magenta	30.286217
GI_3804944	LOC380756	magenta	29.216083
GI_3807826	LOC230075	magenta	29.167647
GI_3808532	LOC213411	magenta	28.497548
scl49167.8_	Rabl3	magenta	28.159676
ri|1700030F	Facl5	magenta	27.767058
scl45267.5.1	1190002H23	magenta	27.454102
scl21336.8_	3110001A13	magenta	26.55687
scl41364.20	Fxr2h	magenta	26.324679
scl074155.4	1300002F13	magenta	26.278075
scl35852.1.8	C030026E1	magenta	26.143512
scl22972.7.8	Pmvk	magenta	25.843827
scl19661.4.1	Ptpla	magenta	25.308754
GI_3478741	Zfp36	magenta	25.103593
scl26886.1_	5830411I20	magenta	24.895412
scl26177.3_	1500011B03	magenta	24.710338
scl0002476.	Kdelr3	magenta	24.113957
scl24653.4_	Rpl22	magenta	24.064087
scl29286.18	Ica1	magenta	24.014718
scl54141.46	Flna	magenta	23.860127
scl26741.21	Gtf3c2	magenta	23.848521
scl23717.7_	D4Ertd196e	magenta	23.4062
scl47027.4_	Zfp251	magenta	23.227409
scl0001766.	Dscr1	magenta	22.525565
GI_1952719	1190002L16	magenta	22.459277
scl36717.10	Rab27a	magenta	22.328173
GI_3809040	LOC380631	magenta	22.306132
scl000897.1	Vamp4	magenta	22.200089
GI_5189022	Nos3as	magenta	21.865467
scl50147.4_	Dusp1	magenta	21.79852
scl24051.10	Slc35d1	magenta	21.738933
scl23093.1.1	Ppm1l	magenta	21.711291
GI_3807755	LOC381490	magenta	21.670648
scl29206.21	Tnpo3	magenta	21.620666
scl52870.7_	Ehd1	magenta	21.429154
scl38001.5.1	1700021F05	magenta	21.303107
scl26467.3.1	Chic2	magenta	20.93412
scl16114.13	Qscn6	magenta	20.658346
scl0002655.	Tpm2	magenta	20.627475

scl068794.1	1110055E19	magenta	20.545476
scl0225341.	Lims2	magenta	20.422404
scl0016997.	Ltbp2	magenta	20.342499
scl071918.2	2310047A01	magenta	19.740194
ri|0610007J.	0610007J10	magenta	19.710154
scl40396.5_	Stc2	magenta	19.625581
scl0074781.	2510001I10	magenta	19.061064
scl19795.10	BC040823	magenta	19.040716
scl0227290.	Aamp	magenta	18.697142
scl31600.13	Pld3	magenta	18.46282
scl0017698.	Msn	magenta	18.265007
scl32504.5.3	Isg20	magenta	18.254939
scl0002171.	Sra1	magenta	18.111218
scl018771.1	Pknox1	magenta	18.066016
scl37690.5.5	Gna11	magenta	18.055951
scl066976.9	2410002F23	magenta	17.738285
scl0071566.	9030425E11	magenta	17.729917
scl54634.28	Drp2	magenta	17.605463
scl31867.15	Lsp1	magenta	17.550112
scl0066878.	Riok3	magenta	17.082383
scl48517.26	Pdir	magenta	16.351701
scl014252.1	Flot2	magenta	15.985006
scl000151.1	AW538196	magenta	15.824393
scl0072278.	D9Ertd392e	magenta	15.625289
scl55020.28	Ube1x	magenta	15.570404
scl39564.8.2	1110036O03	magenta	15.544697
scl17747.8_	4930418P06	magenta	15.44882
scl44027.5.1	5930418K15	magenta	15.089308
scl0056722.	Litaf	magenta	14.829106
scl072795.9	Ttc19	magenta	14.815208
scl29854.5.1	Aup1	magenta	14.724669
scl0110460.	Acat2	magenta	14.641994
scl29611.11	Pparg	magenta	14.275488
scl50602.12	Hdgfrp2	magenta	14.272372
scl014979.1	H2-Ke6	magenta	14.183242
scl40746.1_	Ttyh2	magenta	13.80399
GI_3807983	4930453N24	magenta	13.802939
scl0056325.	Abcb9	magenta	13.202475
scl25143.20	A830039B04	magenta	13.074325
scl14953.2.1	C030004G1	magenta	12.948001
scl25921.12	Tmpit	magenta	12.887651
scl0019042.	Ppm1a	magenta	12.855222
scl49886.18	Slc29a1	magenta	12.853335
GI_3807555	Flnb	magenta	12.804201
scl48649.5.1	5730578N08	magenta	12.66739
scl30406.1.1	2210410E06	magenta	12.641515
scl44364.2.1	C86987	magenta	12.573244
mtDNA_ATP8-S		magenta	12.51562
scl41258.12	Pitpn	magenta	12.453857
scl53221.10	9230117N10	magenta	12.313316
scl22482.15	Mcoln2	magenta	12.245665
scl44552.6_	Hapln1	magenta	11.913241
scl0064138.	Ctsz	magenta	11.566791
scl054608.1	Abhd2	magenta	11.527393

scl38324.4.1	Ddit3	magenta	11.408286
scl070302.4	3110050K21	magenta	11.352969
scl44029.9_	Sca1	magenta	11.346817
scl22769.5.2	Rhoc	magenta	11.280866
ri|3110043M	Chk	magenta	11.092309
scl31661.8_	D7Ertd458e	magenta	11.051845
scl0003767.	Gna11	magenta	11.018688
scl029864.1	Rnf11	magenta	10.999392
scl0276770.	Eif5a	magenta	10.911907
scl31371.4.1	Myd116	magenta	10.806343
scl0017709.	mt-Co2	magenta	10.443799
mtDNA_COXII-S		magenta	10.398357
scl30620.3.1	Cox6a2	magenta	9.712217
scl066158.1	1110012O05	magenta	9.6647494
scl33727.4.1	Crlf1	magenta	9.5710624
scl075445.1	1700008B15	magenta	8.9218468
scl18078.2_	Hs6st1	magenta	8.7832968
scl015013.2	H2-Q2	magenta	8.7300659
scl0002488.	C1qtnf3	magenta	8.5989979
scl44726.4.1	Caml	magenta	8.4607754
scl23088.16	C87860	magenta	8.4514228
scl077569.2	3732412D22	magenta	8.3476364
ri|D630036E	Nfkb1	magenta	8.2536346
scl51506.2_	Cd14	magenta	7.9103741
scl20326.4.1	Dusp2	magenta	7.6404938
scl38757.5.1	Ndg2	magenta	7.6091883
scl18579.23	Rrbp1	magenta	7.60458
scl0218121.	Oact1	magenta	7.4010594
scl0052357.	D8Ertd594e	magenta	7.335869
scl17782.7.1	1110060O18	magenta	7.04218
scl35573.3.1	2410146L05	magenta	6.8808271
scl50221.17	Pdpk1	magenta	6.7734991
scl0001905.1_3-S		magenta	6.5409399
ri|9630055N	9630055N22	magenta	6.0126764
scl20473.1.1	5430416B10	magenta	5.716793
scl49405.9.1	Ntan1	magenta	5.4345201
scl27561.16	Anxa3	magenta	5.3607778
scl46053.11	Elf1	magenta	3.7926391
scl51338.13	Nars	pink	60.178315
scl40250.1.1	5430406J06	pink	58.754259
scl000260.1_64-S		pink	57.270076
scl37695.8.1	Nfic	pink	56.750679
scl0320319.	E330018D0	pink	56.0463
ri|2310014B	2310014B11	pink	54.521574
scl0003358.	Odf2	pink	54.075981
ri|5730439I2	Ephb2	pink	53.732421
scl17934.1.1	Bzw1	pink	53.351506
ri|6430703F	Ptprs	pink	52.796785
ri|9430047F	9430047F21	pink	52.454463
scl020679.1	Sox6	pink	52.436596
GI_3808620	LOC243902	pink	52.354568
ri|D130043G	Ccnl	pink	52.12754
ri|A130019A	A130019A10	pink	51.829203
scl052357.2	D8Ertd594e	pink	51.126275

scl48855.3.1	Cbr1	pink	51.020901
scl39982.2.1	C1qbp	pink	50.319165
scl33370.7_	1810044O22	pink	49.788036
scl24570.12	Tox	pink	49.732612
scl00217995	B130016L12	pink	49.06108
ri|2610021E	Csnk2a1-rs4	pink	48.879709
scl31450.2_	Plekhf1	pink	48.214173
scl0002022.1_2978-S		pink	48.147743
scl0078818.	5830407P18	pink	48.124908
ri|B130016G	Mgea5	pink	47.926898
scl071358.8	Gnas	pink	47.806774
scl40072.13	Map2k4	pink	46.603855
scl00104479	BC018601	pink	46.511356
scl075700.1	1500001E21	pink	46.059449
scl43927.8_	Lman2	pink	45.398785
ri|5730478E	Kpnb3	pink	45.33504
scl012366.1	Casp2	pink	44.801788
ri|C730030N	Stxbp3	pink	43.971781
ri|A330083D	D7Ertd70e	pink	43.897229
ri|G630024G	G630024G0	pink	43.73782
scl0003098.	Snrpb	pink	43.307514
scl32517.1.1	4833416J08	pink	43.195815
scl0021372.	Tbl1x	pink	43.074775
ri|B430201C	Srb1	pink	42.880985
scl0002489.	4921518A06	pink	42.487625
ri|D630035N	D630035N0	pink	41.66658
scl40498.6_	1810003N24	pink	41.411358
scl052857.1	D7Bwg0611	pink	41.395598
GI_3804950	3222401M2	pink	41.300262
scl36525.10	Mrpl3	pink	41.151217
scl27178.12	Cct6a	pink	41.056969
ri|A930006A	A930006A19	pink	40.782416
scl47673.9_	Adpn	pink	40.729016
ri|9530082I1	9530082I15	pink	40.510384
scl027993.9	D1Wsu40e	pink	40.405427
scl41432.3.1	2810001G20	pink	40.400957
scl0003984.	Atp2a2	pink	40.235023
scl0080281.	BC003236	pink	40.170066
scl26663.16	Wdr1	pink	40.165399
scl39143.6.1	Deadc1	pink	40.157476
scl0075745.	Rian	pink	40.126398
scl49386.9_	Ppm1f	pink	39.992962
scl0233912.	Armc5	pink	39.636047
scl50113.16	Srpk1	pink	39.610496
ri|9030016H	9030016H15	pink	39.587126
GI_3809160	LOC382512	pink	39.541404
scl000526.1	Tnfrsf6	pink	39.258441
scl066412.2	Arrdc4	pink	39.05308
ri|9630007E	9630007E23	pink	38.679998
scl0320689.	D930014N2	pink	38.601639
scl0064934.	Pes1	pink	38.388667
scl38668.4.1	Gadd45b	pink	38.1712
scl053881.1	Slc5a3	pink	36.446163
scl0050926.	Hnrpdl	pink	36.244889

scl47392.22	Rai14	pink	36.211017
scl0067379.	Dedd2	pink	36.198597
scl020382.2	Sfrs2	pink	36.143249
scl24620.16	BC004012	pink	36.098336
scl069072.8	Ebna1bp2	pink	36.095998
scl0027407.	Abcf2	pink	35.916061
scl22856.7_	Txnip	pink	35.790242
scl35232.1.:	1700024F2(	pink	35.75323
scl012615.5	Cenpa	pink	35.735067
scl47090.1_	Sf3b4	pink	35.72421
scl067134.7	Nol5a	pink	35.658037
scl0002332.	Dld	pink	35.610695
GI_3323938	E330018D0	pink	35.333626
scl27424.11	Pitpnb	pink	35.265224
ri|A530025E	A530025E0!	pink	34.776522
scl33521.22	Rbl2	pink	34.334376
scl41864.7_	0610042I15	pink	34.308215
scl53392.18	Mta2	pink	33.763732
scl46459.12	Anxa8	pink	33.73417
ri|D230005E	D230005E0	pink	33.61754
scl0023215	Mobk1b	pink	33.552003
scl47422.17	Osmr	pink	33.365007
scl0001674.	Tgif	pink	33.310916
scl0075452.	1700011I11	pink	32.946188
ri|1810026C	1810026C2:	pink	32.868893
scl0003447.	Rbm5	pink	32.687693
scl0069170.	1810026B0!	pink	32.430846
scl0020510.	Slc1a1	pink	32.398786
scl38232.7_	Map3k7ip2	pink	31.940924
scl25081.14	D630045E0	pink	31.764547
scl0002368.1	_75-S	pink	31.706937
GI_3807538	LOC218343	pink	31.669531
ri|D830029A	Cacna2d1	pink	31.283678
scl23105.14	Schip1	pink	31.257303
scl0003800.	Tfam	pink	31.156405
scl067109.1	2210018M0	pink	30.677974
scl33072.15	Trim28	pink	30.596409
scl0022680.	Zfp207	pink	30.411835
GI_8381691	Dusp22	pink	30.236473
scl26951.9_	E430026A0	pink	29.880101
scl056149.9	Grasp	pink	29.602416
scl0019769.	Rit1	pink	29.583296
scl27993.23	Ube3c	pink	29.173127
scl0003647.	Sptlc1	pink	29.052458
scl40772.15	Prkar1a	pink	29.027621
scl16950.11	Tram2	pink	28.870446
scl000274.1	Tnrc6	pink	28.676788
ri|B130049I(	Cd44	pink	28.512041
scl23724.7_	Ppp1r8	pink	28.453261
scl37939.1.:	2510042O1!	pink	28.432464
scl27066.10	1110007L15	pink	28.207664
ri|B130009C	Rbm14	pink	28.167792
scl50873.6.:	1500032D1(	pink	27.968086
scl0320078.	Olfml2b	pink	27.654373

scl40885.12	Psme3	pink	27.509344
ri|B230112G	Itga6	pink	27.290555
scl00214133	E130014J05	pink	26.831918
scl077616.1	C330006D1	pink	26.760973
scl49266.7.1	Hes1	pink	26.10326
ri|C230064E	C230064E0	pink	25.839357
scl54639.14	Cstf2	pink	25.825023
ri|D130072G	Hdac2	pink	25.62488
ri|A430023D	A430023D2	pink	25.263081
scl0320188.	E030045D1	pink	24.901188
scl3244.1.1_	Pnrc1	pink	24.801437
scl32962.7.1	Plaur	pink	24.567274
scl33996.15	Slc20a2	pink	24.42293
scl20721.5_	Ube2e3	pink	24.333023
ri|C130095G	Lpin2	pink	24.150913
scl0035331(	End2	pink	23.992064
scl0012934.	Dpysl2	pink	23.923924
scl00100017	AA691260	pink	23.891705
scl0209318.	Gps1	pink	23.684347
scl49394.3_	Snai2	pink	23.518336
scl014633.1	Gli2	pink	23.304703
scl43914.11	H2afy	pink	23.166103
scl0050884.	Nckap1	pink	22.463336
scl0073827.	1110012D08	pink	22.318657
GI_6754137	H2-Q5	pink	22.252988
scl29632.2_	5830427H10	pink	22.238658
scl080290.2	Gpr146	pink	22.212377
scl32952.5_	2310061G07	pink	21.958986
ri|A530090C	GIG2	pink	21.46718
scl29699.7_	Ppp4r2	pink	21.328736
ri|4930563C	4930563C06	pink	20.672708
ri|6430407L	6430407L02	pink	20.327381
scl000603.1	1810047C23	pink	19.625182
scl00231717	A230106M1	pink	19.434698
scl015122.2	Hba-a1	pink	19.344598
scl023849.4	Copeb	pink	19.225221
ri|0910001P	Hbb-b1	pink	19.204947
scl0014964.	H2-D1	pink	18.121167
ri|2510023M	Hbb-b1	pink	18.052926
ri|0610006C	Hbb-b1	pink	18.00977
scl0019242.	Ptn	pink	17.7138
scl33230.10	Zfpm1	pink	17.484485
ri|2500004H	Hbb-b1	pink	17.295688
scl0240514.	AI842788	pink	17.238938
scl54642.6.1	Tnmd	pink	17.182407
scl077574.1	3321401G04	pink	17.056427
ri|2510028J	Hbb-b1	pink	16.903411
ri|B930001E	KIF17	pink	16.481895
GI_3198260	D17H6S56E	pink	16.31842
ri|A630085A	Usp14	pink	16.223772
scl42956.7_	Jundm2	pink	16.021848
scl25518.12	Rusc2	pink	15.744593
ri|2510040I	Hbb-b1	pink	15.577938
scl19459.18	Fnbp1	pink	15.286269

scl000234.1	Fxyd5	pink	15.110532
scl23301.3_	Cldn11	pink	14.568646
ri|0610037B	0610037B23	pink	14.370768
scl0003099.	Smox	pink	13.955489
ri|2610200H	Gna13	pink	13.806227
scl0001687.	Mylc2b	pink	13.018182
scl020393.1	Sgk	pink	12.885449
scl018637.3	Pfdn2	pink	12.274278
scl0023024S	AI314180	pink	11.481995
ri|C230067C	C230067O0	pink	11.249181
scl42127.12	Lgmn	pink	11.100831
scl072465.7	Zfp131	pink	10.931196
ri|5730494J	5730494J16	pink	9.8454685
GI_3807514	LOC228730	pink	9.5884646
GI_380887C	LOC385019	pink	9.3875119
scl32481.15	Sema4b	pink	9.2089529
ri|A130029H	A130029H0	pink	8.7778388
scl30761.7_	Arl6ip1	pink	8.421688
scl52091.9.	0610010O1	pink	7.3447355
scl22672.6.	F3	pink	7.0293293
scl17283.6_	Slc19a2	pink	6.0200899
scl0022329.	Vcam1	pink	5.7056096
scl016667.1	Krt1-17	pink	5.5749008
GI_3809043	LOC212399	pink	4.0019155
scl42524.16	Scin	pink	3.8520105
scl056628.1	LOC56628	pink	3.5897076
scl0319182.	Hist1h2bh	purple	55.314169
scl0319184.	Hist1h2bk	purple	55.12036
scl0319159.	Hist1h4j	purple	55.005518
scl0319187.	Hist1h2bn	purple	53.544313
scl0326619.	Hist1h4a	purple	53.33616
scl0319183.	Hist1h2bj	purple	53.302242
scl0319185.	Hist1h2bl	purple	52.26714
scl0031914S	Hist1h3d	purple	51.747702
scl0319186.	Hist1h2bm	purple	51.713004
scl0319180.	Hist1h2bf	purple	50.713958
scl0319150.	Hist1h3b	purple	50.517814
scl0319188.	Hist1h2bp	purple	50.257561
scl018655.1	Pgk1	purple	49.154175
scl0319151.	Hist1h3e	purple	48.50486
scl0031919C	Hist2h2be	purple	47.85695
scl20894.20	Pkp4	purple	47.131267
GI_3008971	Hist1h4d	purple	46.773578
scl0319152.	Hist1h3h	purple	46.2914
GI_300897C	Hist1h4m	purple	45.658866
scl44991.2.	Hist1h2bc	purple	45.650669
scl31905.5_	Nalp6	purple	45.61194
scl0031915t	Hist1h4i	purple	45.31126
scl0319153.	Hist1h3i	purple	44.707012
scl27971.8.	Emilin1	purple	44.587301
scl0319179.	Hist1h2be	purple	44.17727
scl018746.1	Pkm2	purple	44.122998
scl054683.1	Prdx5	purple	44.052682
GI_6679936	Gapd	purple	43.536063

GI_6679936	Gapd	purple	43.536063
GI_6679936	Gapd	purple	43.536063
GI_6679936	Gapd	purple	43.536063
scl46303.7_	Lrp10	purple	43.250311
scl000801.1	Lamc2	purple	42.862021
scl0031915	Hist2h3b	purple	42.544708
scl24094.1_	Jun	purple	42.532303
gi_6679936_ref_NM_008		purple	42.280555
scl016828.6	Ldh1	purple	42.18858
ri|C130008L	C130008L1	purple	41.687121
scl31772.7.1	Zim1	purple	41.592
scl0319160.	Hist1h4k	purple	41.455628
scl071434.1	5530400B0	purple	41.244587
scl38942.8.1	B630009I04	purple	40.457844
scl0054683.	Prdx5	purple	40.214694
scl021341.1	Taf1c	purple	39.923582
scl25240.12	Pgm2	purple	39.842644
scl022041.3	Trf	purple	39.835472
scl017319.2	Mif	purple	39.771991
scl0018845.	Plxna2	purple	39.359208
scl19751.3.1	Rpp38	purple	39.289787
scl0010279	Tcta	purple	39.144582
scl26206.11	Myo18b	purple	39.029929
GI_3006135	Hist1h4k	purple	38.823684
scl0031916	Hist1h4m	purple	38.681974
scl0002932.	Dsip1	purple	38.40985
scl47531.14	Tegt	purple	38.313989
GI_3198220	Hist1h2ae	purple	38.299462
scl0001379.1_70-S		purple	38.232256
scl0381493.	S100a15	purple	38.202967
scl0014268	Zcchc14	purple	37.852022
scl21900.2.1	Sprr1a	purple	37.737367
scl35395.12	Dorz1	purple	37.540531
scl46136.14	Loxl2	purple	37.316384
scl0010810	Baiap2	purple	37.302555
scl0031915	Hist1h4f	purple	37.08079
ri|E330037I1	E330037I15	purple	36.471901
ri|A130047M	Arnt	purple	36.08787
scl42728.4.1	Siva	purple	35.887554
scl28683.3_	Chchd4	purple	35.78476
scl016598.3	Klf2	purple	35.483499
scl39569.8.1	Krt1-16	purple	35.227719
scl44212.1.1	Hist1h2ae	purple	34.764454
GI_3807987	LOC239727	purple	34.660818
scl47871.3_	Myc	purple	34.622163
GI_2394392	Hist1h4h	purple	34.617686
scl021991.1	Tpi1	purple	33.990763
ri|C130020C	C130020C0	purple	33.628887
ri|1110008F	2310016E0	purple	33.514848
scl42322.9_	Rab15	purple	32.707774
scl22188.5_	4930583H1	purple	31.615958
scl35393.12	Adprtl3	purple	31.559836
scl014433.2	Gapd	purple	31.15664
scl34226.11	Mvd	purple	30.807977

scl24606.5_	1200013A08	purple	30.763604
scl0023715!	BC024537	purple	30.579633
scl068020.5	2810002N01	purple	30.538319
GI_3807990	LOC383125	purple	30.380491
scl0074182.	2310032D16	purple	30.169588
scl018641.1	Pfkl	purple	29.921262
scl21907.2_	Lor	purple	29.56323
scl011639.5	Ak4	purple	29.053894
scl013806.1	Eno1	purple	29.003427
scl55000.11	Il13ra1	purple	28.859287
scl0010875!	2610208E05	purple	28.772941
scl36353.20	Villp	purple	28.677549
scl36445.9_	Scotin	purple	27.829638
ri|5830469K	5830469K17	purple	26.896279
scl16868.12	Actr1b	purple	26.812303
scl45393.10	Bnip3l	purple	26.646052
scl35434.19	AW107703	purple	26.485794
scl17151.10	C330023F11	purple	26.08234
scl16101.1.1	8430426K15	purple	25.705318
scl47086.3_	Arc	purple	25.514062
scl0018173.	Slc11a1	purple	24.819465
scl48494.5_	Gtf2e1	purple	24.308088
scl43906.11	Trpc7	purple	24.027301
scl0001779.	Eif4a2	purple	23.98292
scl0024229	1700012H17	purple	23.83006
scl0027366.	Txnl4	purple	23.526823
scl50129.4_	D17Wsu92e	purple	23.147349
scl47204.11	Ext1	purple	23.093264
scl19775.24	1700051I12	purple	22.901322
scl46305.13	Mmp14	purple	22.435423
scl0031919	Hist2h2aa2	purple	22.430177
scl37402.3_	Sas	purple	22.418917
scl50694.13	Supt3h	purple	22.241139
scl0016976.	Lrpap1	purple	21.875715
scl0011240!	Egln1	purple	20.836336
scl22134.5_	Pfn2	purple	20.28102
ri|E430003J	E430003J01	purple	19.031111
ri|D130026C	D130026O1	purple	18.007244
scl21672.12	Csf1	purple	17.963879
scl42792.7_	AI132487	purple	17.213228
scl36783.2.1	3300001A09	purple	16.347492
ri|C820018D	N4wbp5-per	purple	16.265169
GI_2079990	Hist2h2aa1	purple	15.931298
GI_3808325	LOC383295	purple	15.555045
scl0014751.	Gpi1	purple	14.300072
scl40921.6_	Igfbp4	purple	13.882199
GI_3705977	Hist2h2ab	purple	13.60776
scl34188.7.1	Acta1	purple	13.078671
scl46340.18	A630038E17	purple	12.836654
scl36619.20	Plod2	purple	12.371149
scl25652.4_	Tmem64	salmon	17.036934
scl40222.12	Slc22a4	salmon	16.400123
scl012575.1	Cdkn1a	salmon	16.395285
scl0228608.	Smox	salmon	15.856785

scl0020963(	Frmd4a	salmon	14.896094
scl0010016(	Phactr4	salmon	14.851813
scl015528.3	Hspe1	salmon	14.69252
scl25869.12	Hrbl	salmon	14.470269
scl0015516.	Hspcb	salmon	14.316075
scl0017936.	Nab1	salmon	14.161544
scl013046.9	Cugbp1	salmon	13.915857
scl000928.1	Hspd1	salmon	13.686272
scl36963.5_	Pou2af1	salmon	13.571389
scl54620.5_	Bhlhb9	salmon	13.181179
scl015519.1	Hspca	salmon	13.048638
scl54754.6.:	Igbp1	salmon	12.743805
gi_7305154_	Hprt	salmon	12.620014
scl52490.15	Smbp	salmon	12.513358
scl066989.6	2410004N1:	salmon	12.495814
scl015516.6	Hspcb	salmon	12.093076
scl22141.1.:	Wwtr1	salmon	11.897275
scl0003360.	Smox	salmon	11.75918
scl0001816.	Eif4a2	salmon	11.500127
scl44868.8_	Gcnt2	salmon	11.425657
scl54358.13	Syn1	salmon	10.973429
scl52902.14	Stip1	salmon	10.582412
scl0015516.	Hspcb	salmon	10.358423
scl44387.13	Plk2	salmon	10.199108
scl19176.21	Stk39	salmon	10.101217
scl37865.9_	D10Ucla1	salmon	10.057752
scl37195.16	Bmper	salmon	9.9587165
scl0170750.	Xpnpep1	salmon	9.2644986
scl24120.2_	Cdkn2b	salmon	8.9740985
scl39328.7_	Grb2	salmon	8.8456547
ri|D130063H	D130063H0	salmon	8.5914904
scl0020964.	Syn1	salmon	8.3431667
scl0003512.	Hspa8	salmon	8.1501901
scl019935.5	Mrpl23	salmon	7.9890494
ri|A830081L	A830081L1!	salmon	6.076359
ri|D030019N	D030019N2	salmon	5.8813089
GI_3808048	LOC270017	salmon	4.0947868
GI_3807557	LOC238943	cyan	12.972684
scl36640.8_	Nt5e	cyan	11.629272
scl0013121.	Cyp51	cyan	11.468866
scl36787.11	Car12	cyan	10.851193
GI_3809024	LOC382127	cyan	10.399759
scl074178.1	2310004N1:	cyan	9.9304332
ri|1110002E	1110002E2:	cyan	9.9221881
scl0003928.	Cdk2	cyan	9.8200061
scl37352.6_	Cdk2	cyan	9.6539513
scl0004183.	Pkm2	cyan	9.5045549
GI_3807707	LOC229810	cyan	8.905672
scl49760.15	Prss15	cyan	8.8185752
GI_2089292	M32486	cyan	8.7030323
scl42308.7_	Rdh11	cyan	8.6426718
scl45525.10	Ripk3	cyan	8.5981429
scl33705.5.:	2010315L1(	cyan	8.437238
ri|A730045A	A730045A1!	cyan	7.4834141

scl073373.8	1700048E23	cyan	6.4933191
scl30961.29	Inppl1	cyan	6.1638376
scl23942.14	Plk3	cyan	6.1023685
scl22623.8.1	Casp6	cyan	5.9161818
scl0071721.	1200015N20	cyan	5.8594437
scl0026430.	Parg	cyan	5.2959256
ri|A730075A	4930563E22	cyan	4.9140201
scl30182.1.1	Luc7l2	cyan	4.50169
scl31352.29	Ush1c	cyan	4.3283141
scl054170.2	Rragc	cyan	4.0897837
scl0022461:	E030034P13	cyan	4.0174498
scl0012725.	Clcn3	cyan	3.9134984
scl073003.5	2900056N03	cyan	3.7224603
scl27250.2_	D730049H0	cyan	3.5555445
scl43672.16	Scamp1	cyan	3.4064398
scl066310.2	2810410M20	cyan	2.8151346
scl23487.8.3	H6pd	cyan	2.4951881
scl24887.7_	Sdc3	cyan	2.3527091
scl51647.25	Npc1	cyan	2.1390087
scl0003240.1_4737-S		cyan	1.6823009
GI_3807612	LOC269355	cyan	1.6510117
scl31868.19	Tnnt3	turquoise	231.48098
scl000091.1	Phf10	turquoise	220.79872
scl078325.1	2700092H06	turquoise	220.15996
GI_3808107	LOC386067	turquoise	218.22287
scl44824.2.2	5033430I15	turquoise	216.74192
scl0110208.	Pgd	turquoise	215.18332
scl0010149	AI194308	turquoise	214.68242
scl000061.1	Nme7	turquoise	214.58543
scl068385.4	0610007A15	turquoise	213.19568
scl50289.12	Phf10	turquoise	211.67287
scl42093.16	Clmn	turquoise	209.91153
scl25663.5_	C130086A10	turquoise	209.65444
scl21413.4_	Cyr61	turquoise	207.55704
scl38235.13	Pcmt1	turquoise	207.2258
scl0002729.	Rgs3	turquoise	206.63376
scl51584.10	Slc39a6	turquoise	206.04517
scl16016.7_	Atp1b1	turquoise	205.95137
scl53247.20	Vldlr	turquoise	204.51366
GI_6755205	Psmb7	turquoise	203.08409
GI_3807995	LOC224163	turquoise	203.03994
scl49870.17	0610041D19	turquoise	201.41738
ri|2210407P	2210407P13	turquoise	200.825
scl38453.7.1	Csrp2	turquoise	200.39214
scl45556.6_	Efs	turquoise	200.29449
scl47746.6_	Maff	turquoise	198.13871
scl49357.1.3	Cldn5	turquoise	198.12311
scl39820.3.1	Ccl5	turquoise	197.41368
scl45762.22	B230373P09	turquoise	195.36101
scl070247.2	Psmd1	turquoise	194.58805
scl37776.6_	D10Jhu81e	turquoise	194.42757
scl0270685.	Fthfsdc1	turquoise	194.30416
scl54770.14	Msn	turquoise	193.15535
scl0020666.	Sox11	turquoise	191.91723

scl0019228.	Pthr1	turquoise	191.61033
scl30803.1.2	E130105L11	turquoise	191.19995
scl019223.1	Ptgis	turquoise	190.09292
GI_3807971	LOC381038	turquoise	189.66043
scl29571.4.1	Ninj2	turquoise	188.85193
scl056292.1	Ard1	turquoise	188.73276
scl29940.6_	Gng12	turquoise	188.5955
scl018616.1	Peg3	turquoise	188.40748
scl32742.5.1	Klk10	turquoise	188.11303
scl0026968	Golga3	turquoise	187.92986
scl000819.1_	3-S	turquoise	187.80661
scl52691.13	Psat1	turquoise	187.65792
GI_7060813	Immt	turquoise	186.83988
scl0017979.	Ncoa3	turquoise	186.56742
scl29349.27	Ppfibp1	turquoise	186.40605
scl076284.1	Xpot	turquoise	186.24397
scl0011931.	Atp1b1	turquoise	186.19439
scl51893.2_	BC020108	turquoise	186.09255
scl0002503.	2610103J23	turquoise	186.06248
scl069860.1	2010003J03	turquoise	185.34546
scl47060.10	9130210N20	turquoise	184.90536
GI_3134060	Tmepai	turquoise	184.7777
GI_7189242	Prr7	turquoise	184.70073
scl50977.11	2400010G15	turquoise	184.38797
scl43025.1.1	Slc39a9	turquoise	184.00956
scl0058996.	4933428G20	turquoise	183.48842
scl067414.1	Mfn1	turquoise	183.45259
ri|2510015F	2510015F01	turquoise	182.36474
scl0018642.	Pfkm	turquoise	181.81299
scl0013640.	Efna5	turquoise	180.77166
scl0018045.	Nfyb	turquoise	180.51989
scl0018227.	Nr4a2	turquoise	180.46281
scl0001058.	Dok1	turquoise	180.25903
GI_2089242	Stfa3	turquoise	180.20579
scl22201.1.6	9930009M0	turquoise	180.08412
scl0093691.	Klf7	turquoise	179.92947
scl0099151.	Ceecam1	turquoise	179.30645
scl45522.6_	9130227C08	turquoise	179.24428
scl0018616.	Peg3	turquoise	178.35528
scl0214895.	Lman2l	turquoise	177.88044
scl0027223.	Trp53bp1	turquoise	177.50373
scl0001280.	Pcyt2	turquoise	175.70851
scl32830.4_	B230312I18	turquoise	175.70767
scl38672.29	Dot1l	turquoise	175.59027
scl056436.9	Adrm1	turquoise	174.55034
scl42959.4_	Fos	turquoise	174.50257
scl0014423.	Galnt1	turquoise	174.42879
scl0023070	Zmpste24	turquoise	174.09456
scl0022214.	Ube2h	turquoise	173.63941
GI_8647607	Pkig	turquoise	173.6192
scl52839.2_	AI837181	turquoise	172.97193
scl39229.10	Pcyt2	turquoise	172.04451
scl40859.13	Grn	turquoise	172.02314
scl0021826.	Thbs2	turquoise	172.00034

scl23824.14	1700029G0	turquoise	171.90557
scl27972.10	Khk	turquoise	171.42631
scl52471.15	Loxl4	turquoise	170.84456
scl25853.12	Pdgfa	turquoise	170.82872
scl0001637.	Luc7l	turquoise	170.63834
scl016779.3	Lamb2	turquoise	170.54601
scl0014481.	Gcap27	turquoise	169.86511
scl31392.8.1	Fcgrt	turquoise	169.42457
scl41429.2.1	Hs3st3a1	turquoise	169.24434
scl012521.1	Kai1	turquoise	169.09837
scl017113.8	M6pr	turquoise	168.63253
GI_3808308	LOC386457	turquoise	168.42219
GI_3807980	LOC224147	turquoise	168.16983
scl068520.7	Zfyve21	turquoise	167.98241
GI_3807952	LOC384146	turquoise	167.95499
scl0014702.	Gng2	turquoise	167.82178
scl0002217.	Nfatc1	turquoise	166.95115
scl32740.6.1	Prss19	turquoise	166.49267
scl43180.27	Mgea6	turquoise	166.42243
ri|C130023K	C130023K0	turquoise	166.23739
scl46497.11	2510015F01	turquoise	166.05156
scl0015507.	Hspb1	turquoise	165.30588
GI_3808952	LOC234882	turquoise	165.23182
scl018227.1	Nr4a2	turquoise	165.03114
scl16161.8_	1700025G04	turquoise	164.7046
scl31502.9_	Fxyd5	turquoise	164.44568
GI_3808218	LOC381092	turquoise	164.3782
GI_3807736	LOC380980	turquoise	164.33922
scl0003713.	Ssbp2	turquoise	164.30153
scl073683.2	2410118P20	turquoise	164.16397
scl0068377.	0610041G09	turquoise	164.03883
scl10895.1.1	6330403L08	turquoise	163.9705
scl34807.5_	1810011E08	turquoise	163.60015
scl50670.2.1	2310039H08	turquoise	163.31792
scl0002412.	Mnat1	turquoise	163.22679
scl22139.3.1	2310058J06	turquoise	162.97965
scl0001889.	Bace2	turquoise	162.83527
scl0022359.	Vldlr	turquoise	162.35012
scl36904.3.1	Cox5a	turquoise	162.25307
scl16099.14	Soat1	turquoise	162.1791
scl011740.4	Slc25a5	turquoise	162.02156
scl30455.5_	Cdkn1c	turquoise	161.44238
scl0068783.	Hnrpa0	turquoise	161.21445
scl15767.4_	Atf3	turquoise	161.19723
scl39361.6_	Cdc42ep4	turquoise	160.97314
scl076478.1	2410004L22	turquoise	160.33888
scl54613.4.1	Ngfrap1	turquoise	160.14974
scl43398.10	Laptm4a	turquoise	159.92403
scl069477.2	2300009N04	turquoise	159.83482
scl0010895	Ppp1r15b	turquoise	159.51935
scl54760.5_	Efnb1	turquoise	159.26139
scl21996.1.1	6720469N11	turquoise	158.89488
GI_3807615	LOC219049	turquoise	158.83526
scl33805.3.1	Scrg1	turquoise	158.75487

scl40552.11	Pold2	turquoise	158.52611
scl19951.7_	Ywhab	turquoise	158.47753
scl014863.1	Gstm2	turquoise	158.45575
scl35670.14	Tpm1	turquoise	158.08247
scl39796.13	Appbp2	turquoise	157.02419
scl50004.2.6	Hspa1b	turquoise	157.00875
scl015384.1	Hnrpab	turquoise	156.84276
scl49975.4.2	Tubb5	turquoise	156.64688
scl39241.9.1	2310003H01	turquoise	156.06899
scl26735.47	Ift172	turquoise	155.04875
scl0071911.	Bdh	turquoise	154.88525
scl0020750.	Spp1	turquoise	154.8145
scl38049.5.1	1700025K23	turquoise	154.68677
scl37356.11	Pa2g4	turquoise	154.21455
ri|6720460L0	6720460L05	turquoise	154.14524
scl46294.4.1	Cklfsf5	turquoise	153.98349
GI_2849155	LOC235427	turquoise	153.8304
scl21323.5.1	1110017I16	turquoise	153.43867
scl013800.1	Enah	turquoise	153.23706
scl39207.10	1110031I02	turquoise	153.11416
scl33605.4_	Dnajb1	turquoise	153.0627
scl069126.1	1810022K09	turquoise	152.84481
scl24582.2_	Penk1	turquoise	152.19248
scl012606.3	Cebpa	turquoise	152.07491
scl22567.17	Ppp3ca	turquoise	152.01158
scl47047.39	Plec1	turquoise	151.67958
scl054324.3	Arhgef5	turquoise	151.49111
scl0017294.	Mest	turquoise	151.38955
scl0056357.	Ivd	turquoise	151.33057
scl39662.12	Cdk5rap3	turquoise	151.06667
scl0002692.	Galt	turquoise	150.81102
scl53763.13	Capn6	turquoise	150.41663
scl36630.14	Ctsh	turquoise	149.79258
scl23109.16	1200003O06	turquoise	149.37957
scl32844.9.1	9430029K10	turquoise	148.8964
scl36669.38	Myo6	turquoise	148.31016
scl29881.14	Capg	turquoise	147.99315
scl00224640	Lemd2	turquoise	147.87576
scl20017.4.1	1110008F13	turquoise	147.79376
scl48137.14	Oxct1	turquoise	147.45082
scl000771.1	Col9a1	turquoise	147.40448
scl0003998.1_329-S		turquoise	147.00364
GI_3804969	LOC277856	turquoise	146.74164
scl28933.2.1	Gadd45a	turquoise	146.16734
scl021833.8	Thra	turquoise	145.92324
scl17368.14	Glt25d2	turquoise	145.65695
scl30187.2.1	1110001J03	turquoise	145.59733
scl24656.12	Bach	turquoise	145.55654
scl51469.24	Lars	turquoise	145.31575
scl30629.5.1	Vkorc1	turquoise	145.24714
GI_3804951	LOC381259	turquoise	145.03024
scl36939.8_	Dnaja4	turquoise	144.81432
scl30680.4.1	Nupr1	turquoise	144.55717
scl0074451.	4933424M2	turquoise	144.03686

scl0001167.	Lmcd1	turquoise	143.64126
scl22221.2_	E430012K2(	turquoise	143.59115
GI_3198209	Prkg2	turquoise	143.20546
scl42588.12	Rnf144	turquoise	143.17721
GI_3808342	LOC271505	turquoise	142.98702
scl0067154.	2610103J23	turquoise	142.81579
scl023897.2	Hs1bp1	turquoise	142.4932
GI_2089209	LOC224048	turquoise	142.28657
scl21156.17	Gpsm1	turquoise	142.28309
scl016832.2	Ldh2	turquoise	142.16134
scl0001617.1_31-S		turquoise	141.97711
scl0002961.	Srpx	turquoise	141.7624
scl0001394.	Pmp22	turquoise	141.37584
scl27779.8_	Klf3	turquoise	141.23214
scl016418.1	Itgb4bp	turquoise	140.86073
scl027373.3	Csnk1e	turquoise	140.47575
scl067941.3	Rps27l	turquoise	139.30434
scl27213.3_	3110032G18	turquoise	138.91035
scl29516.6.1	Cdca3	turquoise	138.71596
scl014114.1	Fbln1	turquoise	138.22674
scl34222.7_	Rnf166	turquoise	138.12588
scl0066875.	1200016B10	turquoise	137.99273
ri|1810037G	0610007N19	turquoise	137.8644
scl0246154.	Slitl2	turquoise	137.84164
scl22980.22	Thbs3	turquoise	137.3271
scl33129.9_	Suv420h2	turquoise	137.30629
scl22881.8.1	Ctsk	turquoise	136.8724
scl0003466.	Ilf3	turquoise	136.28477
GI_3808569	2210018M0	turquoise	136.26757
scl0066882.	Bzw1	turquoise	136.12512
scl53077.5.8	Gsto1	turquoise	135.43141
scl0230234.	BC026590	turquoise	135.42864
scl069601.7	Dab2ip	turquoise	135.22274
scl41755.17	AW011752	turquoise	134.86988
scl0018569.	Pdcd4	turquoise	134.85744
scl0231070.	Insig1	turquoise	134.59905
scl017347.2	Mknk2	turquoise	133.48859
scl39222.8.1	Cbr2	turquoise	133.43375
scl45595.9_	Hnrpc	turquoise	133.14625
scl44050.2_	AI850995	turquoise	132.62438
scl0067163.	2610204L23	turquoise	132.5033
scl0070387.	2210019E14	turquoise	131.98125
scl35489.7_	Atp1b3	turquoise	131.50936
scl022154.1	Tubb5	turquoise	131.46145
scl51588.6_	D030070L09	turquoise	131.35599
scl022253.1	Unc5c	turquoise	131.34851
scl071436.1	Flrt3	turquoise	130.76247
GI_3808357	3930401E15	turquoise	130.62167
scl052064.7	D5Ertd33e	turquoise	130.04375
scl19446.4_	Lcn2	turquoise	130.04318
scl53290.5_	5730446C15	turquoise	129.72304
scl24819.10	Tcea3	turquoise	129.10994
GI_8479459	Ppp3r1	turquoise	128.85423
scl0001911.	Umps	turquoise	128.57847

scl0012793.	Cnih	turquoise	128.48954
scl0107995.	Cdc20	turquoise	128.43532
scl35308.7.1	Tmie	turquoise	128.41893
scl19923.3_	Zswim1	turquoise	128.37677
scl0018810.	Plec1	turquoise	128.12268
scl000533.1	Yif1	turquoise	128.04542
scl0320467.	9330147J08	turquoise	127.99618
scl18972.12	1810020C19	turquoise	127.94579
scl22424.8_	Zfp265	turquoise	127.87255
scl0002876.	Ard1	turquoise	127.51188
scl22977.3.9	Dpm3	turquoise	127.50997
scl23625.5_	Nbl1	turquoise	127.35264
scl0002549.	Rbm9	turquoise	127.33418
scl24418.2_	2310040A07	turquoise	127.21894
scl0226334.	Arfgef1	turquoise	127.14363
scl26345.11	Antxr2	turquoise	127.03914
ri|B130053I1	B130053I10	turquoise	126.82918
scl000963.1_	12-S	turquoise	126.78922
scl17978.10	5330401P04	turquoise	126.78419
scl000209.1_	5-S	turquoise	126.48183
scl29744.12	1200015A22	turquoise	126.35922
scl0022769З	C230075L19	turquoise	126.18563
scl48021.6_	Dap	turquoise	125.83619
scl51921.9.1	1190002C06	turquoise	125.26696
scl0320398.	Lrig3	turquoise	125.10096
scl073102.1	3110004L20	turquoise	125.03016
scl36684.10	Elovl5	turquoise	125.00044
scl37887.24	Ccar1	turquoise	124.75457
scl0021339.	Taf1a	turquoise	124.68519
ri|9830169E	9830169E20	turquoise	124.41457
GI_3808480	2010003J03	turquoise	124.41131
scl21063.24	Golga2	turquoise	124.07729
scl0100986.	Akap9	turquoise	124.02192
scl018574.1	Pde1b	turquoise	123.96551
scl25735.19	Hsp105	turquoise	123.83508
scl17376.15	Ivns1abp	turquoise	123.24849
scl17803.10	Arpc2	turquoise	123.10974
scl0003597.	Mmp3	turquoise	122.81747
scl0058194.	Sh3kbp1	turquoise	122.32195
scl52979.12	Pdcd4	turquoise	121.85229
scl50604.11	E130307M0	turquoise	121.81334
scl018458.3	Pabpc1	turquoise	121.71168
scl52692.1_	Eif4a1	turquoise	121.69893
scl51108.6.3	Sod2	turquoise	121.6217
scl0066102.	Cxcl16	turquoise	121.6066
scl00214572	BC006705	turquoise	121.38763
scl067581.1	4930451A13	turquoise	120.45666
scl51547.21	2610024E20	turquoise	120.45419
scl0001957.	Thbs3	turquoise	120.44699
GI_3807655	LOC242051	turquoise	120.33495
scl0022193.	Ube2e3	turquoise	120.22355
scl014528.1	Gch1	turquoise	119.74859
scl0023107(	Insig1	turquoise	119.61394
scl0001450.	1700113I22	turquoise	119.34569

scl38781.10	Zwint	turquoise	118.98896
GI_3805038	Hectd1	turquoise	118.94483
scl47703.8_	Tef	turquoise	118.62081
scl0017716.	mt-Nd1	turquoise	118.49315
scl0017967.	Ncam1	turquoise	117.89227
ri|B130015M	B130015M1	turquoise	117.79724
GI_3808040	LOC384206	turquoise	117.70239
scl018218.1	Dusp8	turquoise	117.6987
scl000237.1	Vkorc1	turquoise	117.6474
scl38691.9.1	Midn	turquoise	117.60797
scl20039.1_	Sdbcag84	turquoise	117.41024
scl28385.5_	Ccnd2	turquoise	117.40047
scl000679.1	1810045K07	turquoise	117.27903
scl022793.9	Zyx	turquoise	116.82199
scl31535.10	Capns1	turquoise	116.7055
scl0021416.	Tcf7l2	turquoise	116.59023
scl0002676.	Lepre1	turquoise	116.55682
scl0230863.	BC036961	turquoise	116.34366
scl0003607.	Il1rl1l	turquoise	116.03937
scl0320267.	Fubp3	turquoise	115.66227
scl51764.24	4933427L07	turquoise	115.12348
scl32000.4_	Bag3	turquoise	114.90351
scl53913.2_	Zcchc5	turquoise	114.73775
scl20092.24	Dnmt3b	turquoise	114.31018
scl0264895.	BC018371	turquoise	114.28568
scl53103.12	Entpd7	turquoise	113.91141
scl36223.4.1	2310067E08	turquoise	113.85203
scl0067296.	Socs4	turquoise	113.59335
scl50160.5.1	BC008155	turquoise	113.53194
ri|D730045B	D730045B0	turquoise	113.48591
scl35939.21	Ube4a	turquoise	113.38292
scl42611.4_	Trib2	turquoise	113.09717
scl0014567.	Gdi1	turquoise	112.83444
scl056401.1	Lepre1	turquoise	112.83
scl084113.2	Ptov1	turquoise	112.65679
scl057783.1	Tnip1	turquoise	112.62149
GI_3808916	LOC384790	turquoise	112.59964
GI_2212251	Mgea6	turquoise	112.4548
scl32192.19	Ampd3	turquoise	112.41824
scl0003933.1_1635-S		turquoise	112.03422
scl0018590.	Pdgfa	turquoise	111.87363
scl47744.5.4	Kdelr3	turquoise	111.372
scl059092.1	Pcbp4	turquoise	111.13671
scl24011.4.1	Gpx7	turquoise	110.93675
scl0021489	Lman2l	turquoise	110.70384
scl15769.9_	B130052G0	turquoise	110.32075
scl46160.15	Clu	turquoise	110.25321
scl28399.6.2	Cd9	turquoise	110.21416
ri|1110025O	Auh	turquoise	110.01322
scl14832.1.1	Slc14a1	turquoise	110.00094
scl54622.12	Gprasp1	turquoise	109.87641
scl016201.1	Ilf3	turquoise	109.81373
scl021933.8	Tnfrsf10b	turquoise	109.74262
scl0270058.	Bpy2ip1	turquoise	109.30578

scl38706.14	Ptbp1	turquoise	109.29508
scl29120.2_	1110014O2(	turquoise	109.294
scl40177.4_	2410016F01	turquoise	108.32874
scl0117109.	Pop5	turquoise	108.2975
scl0226980.	Eif5b	turquoise	107.50898
scl0014571.	Gpd2	turquoise	107.13505
scl0014733.	Gpc1	turquoise	106.95831
scl34564.9_	4930527D1	turquoise	106.91835
scl0106042.	Prickle1	turquoise	106.64145
scl056348.1	Hsd17b12	turquoise	106.39938
scl34239.10	Fbxo31	turquoise	105.96791
scl0068796.	1110039B1	turquoise	105.9501
scl070083.1	Metrn	turquoise	105.45721
scl19731.15	Optn	turquoise	105.03384
GI_3808632	LOC382215	turquoise	104.66262
scl50005.1_	Hspa1a	turquoise	104.5802
scl076437.1	D11Bwg041	turquoise	104.54193
scl52093.1_	6330403M2	turquoise	104.33706
GI_3808012	LOC383189	turquoise	103.74499
GI_6677808	Rps6	turquoise	103.45202
scl052398.2	D5Ertd606e	turquoise	103.07423
scl018744.1	Pja1	turquoise	102.97923
scl41842.10	Upp1	turquoise	102.38078
scl078578.3	D530030D0	turquoise	102.23678
scl33714.1_	Jund1	turquoise	102.21742
scl056812.9	Dnajb10	turquoise	102.19722
scl32668.4.1	Dbp	turquoise	101.94591
scl013723.9	Emb	turquoise	101.59771
scl44989.1_	Hist1h1c	turquoise	101.35031
scl054194.1	Akap8l	turquoise	101.16496
scl067529.7	Fgfr1op2	turquoise	100.92152
scl069763.1	1810013H0	turquoise	100.80282
scl0002845.	Capzb	turquoise	100.65624
scl36607.9.1	Pcolce2	turquoise	99.982008
scl29462.4_	Gabarapl1	turquoise	99.760592
scl026425.1	Nubp1	turquoise	99.596779
scl068337.8	Crip2	turquoise	99.140481
scl068776.1	Taf11	turquoise	99.051829
ri|2610028H	2610028H0	turquoise	98.931213
scl53842.9_	Tm4sf6	turquoise	98.328619
scl22869.17	BC051083	turquoise	98.2771
scl18950.6_	Trim44	turquoise	97.930397
scl35515.3_	1200002G1	turquoise	97.756163
scl40797.10	Psmc5	turquoise	97.545493
ri|4833426I2	Loxl4	turquoise	97.424969
scl33912.4_	Dusp4	turquoise	97.359911
scl31391.7.1	Rcn3	turquoise	97.305712
scl38623.34	A230046K0	turquoise	97.266803
scl30702.10	2510027N1	turquoise	96.941654
scl0072147.	Btbd4	turquoise	96.710525
scl019068.1	Erh	turquoise	96.542629
scl39533.9.1	1700113I22	turquoise	96.391675
scl20104.7.1	Mylk2	turquoise	96.243914
scl47811.2_	2610029D0	turquoise	95.630709

scl0019055.	Ppp3ca	turquoise	95.076721
scl31698.2_	Foxa3	turquoise	94.969665
scl23434.8.:	Mmp23	turquoise	94.861283
scl018032.3	Nfix	turquoise	94.455539
GI_3198132	Psmb3	turquoise	94.120462
scl0212111.	Inpp5a	turquoise	94.113883
scl018542.3	Pcolce	turquoise	93.528596
scl51111.10	Tcp1	turquoise	93.198476
scl18646.8.:	4931426K1(	turquoise	93.000301
scl0003930.	Bloc1s1	turquoise	92.901209
scl0002866.	Bach	turquoise	92.723065
scl0022722.	Zfp64	turquoise	92.509377
scl36342.8.:	BC010801	turquoise	92.246959
scl0002734.	Ssbp3	turquoise	92.228491
scl0014700.	Gng10	turquoise	91.909343
scl012013.5	Bach1	turquoise	91.895012
ri|6330409F	6330409F21	turquoise	91.737179
scl42750.13	Tnfaip2	turquoise	91.521758
scl52609.1.:	4833409N0:	turquoise	91.497058
ri|1500006G	Fnbp2	turquoise	91.455342
scl39080.13	Moxd1	turquoise	91.341838
ri|2610306C	Pde4b	turquoise	91.201135
scl0019219	Bcas3	turquoise	90.913687
GI_3809162	LOC277016	turquoise	90.701863
scl0069472.	2310006E1:	turquoise	90.58498
scl0001364.	Ppia	turquoise	89.9249
scl54136.4_	Slc10a3	turquoise	89.719353
scl25760.31	Flt1	turquoise	89.515371
scl099451.1	Mylk2	turquoise	89.203447
scl0020788.	Srebp2	turquoise	89.172932
scl25485.5.:	Zcchc7	turquoise	88.998833
GI_2174616	2410129E1	turquoise	88.814972
GI_2174616	2410129E1	turquoise	88.814972
scl6307.1.1_	B230112P1:	turquoise	88.231939
scl018521.1	Pcbp2	turquoise	88.111245
scl32303.22	Fchsd2	turquoise	87.859093
scl015526.3	Hspa9a	turquoise	87.2255
GI_3807589	E430019N2	turquoise	86.955333
ri|3930401E	3930401E1!	turquoise	86.104349
scl46777.9.:	Col2a1	turquoise	85.836053
scl37307.8.:	Mmp3	turquoise	85.692495
GI_3808289	Crim1	turquoise	85.503414
scl32577.4.:	Mcee	turquoise	85.430901
scl0012575.	Cdkn1a	turquoise	84.672984
scl012864.3	Cox6c	turquoise	83.759091
GI_3807754	LOC223594	turquoise	83.374947
scl50998.1_	Mrps34	turquoise	82.910251
scl020312.5	Cx3cl1	turquoise	82.634748
scl000293.1	Cldn10	turquoise	82.514821
scl0075710.	Rbm12	turquoise	82.400318
scl021933.8	Tnfrsf10b	turquoise	82.22853
scl067049.4	Pus3	turquoise	81.97448
GI_3807716	D330037H0	turquoise	81.516979
scl32856.10	Ech1	turquoise	79.969184

scl47123.18	Ndrg1	turquoise	79.340402
GI_3808310	LOC333744	turquoise	79.267603
scl0108991.	1700001A24	turquoise	79.004318
scl0002222.	Lims2	turquoise	78.988726
ri|9630032J0	9630032J03	turquoise	78.904629
scl26469.13	Scfd2	turquoise	78.763146
scl0064292.	Ptges	turquoise	78.485383
ri|9130422H	9130422H11	turquoise	78.215226
scl35011.1.3	1810011O10	turquoise	77.737525
scl45706.11	Ghitm	turquoise	77.581402
scl46617.10	Il3ra	turquoise	77.012683
scl23689.13	Extl1	turquoise	76.778864
ri|A730060B	Epb4.1l2	turquoise	76.390757
scl33398.3_	AW539964	turquoise	76.334238
scl0321022.	Cdv3	turquoise	76.133439
scl0067464.	Entpd4	turquoise	75.882346
ri|A730020F	Sema6a	turquoise	75.601721
scl027366.4	Txnl4	turquoise	75.02643
scl0226971.	Plekhb2	turquoise	74.383694
scl070312.7	2510012J08	turquoise	73.221387
scl39478.12	Plekhm1	turquoise	73.217052
scl25185.2.3	Ppap2b	turquoise	73.180663
scl46585.22	Vcl	turquoise	72.609181
GI_3134092	9130415E20	turquoise	72.510534
scl0003187.1	_40-S	turquoise	72.385077
scl31959.7.1	Bccip	turquoise	72.187856
scl0056473.	Fads2	turquoise	72.163699
scl00234839	2310061F22	turquoise	72.030553
scl39472.24	Nsf	turquoise	71.840139
scl31589.11	Psmc4	turquoise	71.787288
scl068728.7	Trp53inp2	turquoise	71.778148
ri|1110002D	1110002D22	turquoise	71.360249
scl075909.1	4930579A11	turquoise	71.222606
scl48036.13	Ank	turquoise	70.396613
scl24417.1.3	Dctn3	turquoise	70.033625
GI_3808695	LOC384646	turquoise	69.989219
scl0002945.	Itm2a	turquoise	69.580095
scl0233552.	BC024955	turquoise	69.53378
scl46959.14	2610007K22	turquoise	69.513226
scl000645.1	2010315L10	turquoise	68.935728
scl0230088.	B230312A22	turquoise	68.409865
scl29624.16	Irak2	turquoise	68.245834
scl19931.7.1	1600023A02	turquoise	67.75759
scl21113.26	Rapgef1	turquoise	67.67014
scl31688.5.1	Fosb	turquoise	67.474808
scl017990.3	Ndrl	turquoise	67.354738
scl32669.8.1	Car11	turquoise	67.319094
scl23564.6_	Gp38	turquoise	67.236364
scl18361.12	Matn4	turquoise	67.169897
scl0011975.	Atp6v0a1	turquoise	65.762623
GI_2142684	Pea15	turquoise	65.619528
scl44527.2.2	C330006P0	turquoise	63.824111
ri|C230060D	Kcnma1	turquoise	63.820846
GI_3807997	LOC328703	turquoise	63.087317

GI_3807731	LOC383010	turquoise	63.035582
scl26834.2.1	2900022P04	turquoise	62.391321
scl43495.3.1	2310016C16	turquoise	61.969212
ri|C430002D	C430002D1	turquoise	61.55639
GI_3108885	H2-D1	turquoise	61.527955
scl31127.23	AW538196	turquoise	61.188358
scl074569.1	Ttc17	turquoise	61.125311
scl41618.6.1	Rnf130	turquoise	60.929179
scl26166.1_	Cd8b	turquoise	60.898353
scl0212427.	A730008H2	turquoise	59.92596
GI_2145078	Ctsd	turquoise	59.606375
GI_3809341	LOC385065	turquoise	59.285269
scl41467.7_	Mfap4	turquoise	59.22336
scl32642.3_	1810054O13	turquoise	59.19108
ri|4921514E	4921514E18	turquoise	58.595497
scl54745.40	Tnrc11	turquoise	58.480993
scl0002782.	Elovl1	turquoise	58.271108
GI_2853704	LOC235973	turquoise	58.016613
scl19890.4.1	1500012F01	turquoise	57.537606
scl0067916.	Ppap2b	turquoise	57.444681
scl0244059.	Chd2	turquoise	57.14115
scl31074.15	Fah	turquoise	57.114047
scl0066819.	9130422G05	turquoise	55.506822
scl0243846.	Ccdc9	turquoise	55.308804
scl25133.32	Nrd1	turquoise	55.139035
scl070835.2	Prss22	turquoise	53.452094
scl054325.6	Elovl1	turquoise	53.006624
scl1566.1.1_	2810423A18	turquoise	52.719197
scl49300.12	St6gal1	turquoise	51.352952
scl011799.4	Birc5	turquoise	50.709866
scl53313.7_	Gna14	turquoise	49.698465
scl53733.14	Maged2	turquoise	49.411565
scl28749.2.2	Pcbp1	turquoise	48.750649
scl026987.6	Eif4el3	turquoise	46.780322
scl000160.1	1500016L11	turquoise	45.95141
scl27358.15	Pxn	turquoise	42.396564
scl38726.3.1	Cstb	turquoise	39.921419
GI_3807788	LOC229599	turquoise	39.42167
scl29006.2.1	Hoxa5	turquoise	32.164258
scl0083925.	Trps1	turquoise	30.795115
scl26782.45	LOC381621	turquoise	30.458691
scl0001186.	Csda	turquoise	27.317999
scl0002344.	Serpina1b	turquoise	24.997591
scl066894.8	1300010O06	turquoise	24.103196
scl016974.1	Lrp6	turquoise	18.79073
scl068559.1	Pdrg1	turquoise	13.889748
scl17665.8_	Cops8	turquoise	11.538002
scl45491.13	Cryl1	turquoise	11.18442
ri|D130017D	D130017D1	turquoise	9.2161371
scl0234388.	1810023B24	turquoise	8.5177384
GI_3808628	Tpt1	tan	18.873265
GI_3808627	LOC384596	tan	17.917794
scl075084.2	LOC384593	tan	16.798429
scl0022070.	4930511M0	tan	16.683246

GI_3809026	LOC244710	tan	16.4104
scl45264.8.1	Lect1	tan	16.086938
scl0015982.	Ifrd1	tan	15.863101
GI_3807534	LOC383775	tan	15.565049
scl44903.11	Fars1	tan	15.395028
scl019946.1	Rpl30	tan	15.159986
scl44126.11	Gmds	tan	15.015597
GI_3807670	LOC234640	tan	14.882947
scl25177.9.1	Dhcr24	tan	14.83931
scl51127.13	Agpat4	tan	13.971159
GI_3808788	LOC384710	tan	13.424158
scl067302.8	3110050K21	tan	13.195759
scl077134.2	Hnrpa0	tan	12.939445
GI_3006135	Hist1h4f	tan	12.801884
scl0003108.	Trib3	tan	12.109416
GI_3006140	Hist1h4a	tan	11.959082
scl52838.4_	Fosl1	tan	11.593824
scl0019244.	Ptp4a2	tan	10.772291
scl074754.9	Dhcr24	tan	10.531905
scl40035.13	Jmjd3	tan	9.8928238
scl25655.8_	2610319K07	tan	9.485987
scl0170707.	Usp48	tan	9.316007
scl50269.3.1	Has1	tan	9.2405026
scl20449.19	Thbs1	tan	8.5803576
GI_1350762	Ifitm2	tan	8.161102
scl0021825.	Thbs1	tan	8.1306904
scl072462.6	2600005C20	tan	7.7958632
scl54248.9.1	Gpc3	tan	7.7019771
scl0001602.1_506-S		tan	7.6605375
scl16486.1.1	Col6a3	tan	7.3603903
scl0011386	Acaa1	tan	7.166207
ri|2010007K	2010007K12	tan	6.8656757
scl44225.1.1	Hist1h4i	tan	6.7838678
scl37947.10	Tde2	tan	6.7229216
scl0003577.	Lrrc1	tan	6.7219594
scl027425.3	Atp5l	tan	6.717891
scl42320.8_	Max	tan	6.617675
scl16146.28	Lamc1	tan	6.171293
scl014980.6	H2-L	tan	6.1070723
scl00022.1_	Actn4	tan	6.0744139
scl069668.1	2310061I09	tan	5.9316799
scl37440.14	Irak3	tan	5.6734674
GI_6680990	Cox7c	tan	5.5777209
scl0020218.	Khdrbs1	tan	5.4637985
scl0226829.	C130080N2	tan	5.2477397
scl45975.11	Gpc6	tan	5.0782553
scl066953.9	Cdca7	tan	5.0453748
scl0003527.	Ddx6	tan	4.9839599
scl0010815	Ogt	tan	4.9738201
scl35598.8_	Mapk6	tan	4.9165981
scl0002832.	Ptp4a2	tan	4.8883056
scl016010.7	Igfbp4	tan	4.7356485
scl17374.15	1190005F20	tan	4.7198321
scl28975.15	Card4	tan	4.6673702

scl27967.7.1	Abhd1	tan	4.6359067
scl0032836!	BC065120	tan	4.6058983
scl26046.6_	BC026744	tan	4.5279718
scl0017705.	mt-Atp6	tan	4.4754255
scl068938.1	Aspscr1	tan	4.3687666
scl013030.1	Ctsb	tan	4.2998199
scl50230.5.1	2810417J12	tan	4.1376978
scl48728.3.1	2410018G2(	tan	4.0998804
scl067078.2	1700012G1!	tan	4.0816432
scl44526.12	Homer1	tan	4.0476644
scl022784.1	Slc30a3	tan	3.8887644
ri|1110070C	1110070O1!	tan	3.808287
ri|6430524C	6430524C0!	tan	3.7862053
scl000502.1	Prdx5	tan	3.7017255
scl49164.12	Fstl1	tan	3.6518607
scl0208606.	1500011J06	tan	3.63939
scl19566.6.1	Dpp7	tan	3.4695649
scl41403.1.1	B230343A1(	tan	3.4561129
ri|2010305C	2010305C0:	tan	3.3417134
ri|2310079P	2310079P0:	tan	3.2887544
scl0023550!	Cd109	tan	3.1859645
scl49257.8_	Bdh	tan	3.1811696
scl49743.39	C3	tan	3.0892191
scl0010885	Ankhd1	tan	3.0338201
scl43611.1.1	5330431K0:	tan	3.0284361
scl20421.3_	1810008K0:	tan	2.7545121
scl23602.11	1110005F07	tan	2.6083305
scl0002013.	Pip5k1b	tan	2.5697174
scl0067665.	Dctn4	tan	2.4714043
ri|B930085B	B930085B1	tan	2.420158
scl32477.13	Prc1	tan	2.4158227
scl30510.5_	Bet1l	tan	2.2613168
scl00001.1_0_REVCOM		tan	2.1948252
scl49691.9_	1700093E07	tan	2.1784131
scl0237436.	Gas2l3	tan	2.1470718
scl0069102.	1810015C1:	tan	1.9831432
scl50794.7.1	Clic1	tan	1.9217486
scl000673.1	Prdx2	tan	1.7744402
scl43966.3_	Nfil3	tan	1.6359154
ri|4833436C	Ybx3	tan	1.6094789

ı the cartilage gene co-expression network
