# Supplementary material for: Systems Genetics Analysis of Mouse Chondrocyte Differentiation
Source: J Bone Miner Res. 2010 Oct 14;26(4):747–60. doi: 10.1002/jbmr.271 (PMC3179327; doi:10.1002/jbmr.271)
Supplement: Supplementary file 7 [file jbmr0026-0747-SD7.pdf]

					log2(fold-change from day 3)				Mean expression values (RMA)					
probe_id	gene symbol	chr	start	end	day 6	day 9	day 12	day 15	day 3	day 6	day 9	day 12	day 15	variance
1417484_at	Ibsp	5	104728190	104740488	3.0875	6.1832	7.1605	6.9289	4.3807	7.4682	10.5640	11.5412	11.3096	9.4770
1417485_at	Ibsp	5	104728190	104740488	1.3525	4.7175	5.6956	5.3673	5.1463	6.4987	9.8638	10.8419	10.5136	6.6562
1418726_a_at	Tnnt2	1	137737452	137748837	-1.6133	-5.2229	-5.5952	-5.3288	9.8015	8.1881	4.5786	4.2063	4.4727	6.6246
1417256_at	Mmp13	9	7272545	7283331	3.2963	5.0602	6.0448	6.0346	4.4149	7.7112	9.4750	10.4597	10.4495	6.4755
1448595_a_at	Bex1	X	132748511	132750052	-1.7453	-4.0470	-4.9333	-5.6235	11.8259	10.0806	7.7790	6.8926	6.2025	5.4816
1423547_at	Lyz2	10	116714389	116719320	1.3176	3.6963	4.8310	5.3881	8.5194	9.8370	12.2157	13.3504	13.9075	5.3401
1439426_x_at	Lyz1	10	116724853	116729924	0.9650	3.4918	4.6792	5.2171	7.5094	8.4743	11.0012	12.1886	12.7265	5.2587
1436996_x_at	Lyz1	10	116724853	116729924	1.2399	3.6543	4.7716	5.1459	8.6815	9.9214	12.3358	13.4531	13.8273	5.0656
1435477_s_at	Fcgr2b	1	172890689	172906678	1.3106	3.2644	4.8929	5.1638	6.3033	7.6140	9.5678	11.1963	11.4672	5.0405
1415927_at	Actc1	2	113873018	113879284	-2.1644	-4.5043	-5.0447	-5.0438	12.9052	10.7409	8.4010	7.8605	7.8615	4.9254
1427115_at	Myh3	11	66891802	66915788	-2.3589	-4.6791	-5.0260	-4.9943	9.8356	7.4767	5.1565	4.8096	4.8413	4.8663
1427013_at	Car9	4	43519838	43526601	4.2509	5.1077	4.9645	4.8184	6.8166	11.0675	11.9243	11.7811	11.6350	4.6856
1423506_a_at	Nnat	2	157385846	157388258	-2.7853	-4.0714	-4.9131	-5.0134	11.8309	9.0455	7.7595	6.9178	6.8175	4.3179
1448371_at	Mylpf	7	134355122	134357812	-1.7814	-4.0096	-4.6979	-4.6442	11.2937	9.5123	7.2841	6.5958	6.6495	4.2717
1420394_s_at	Lilrb4	10	51200446	51216419	0.6080	2.5168	4.0525	4.6400	6.7196	7.3275	9.2364	10.7721	11.3595	4.1816
1424967_x_at	Tnnt2	1	137737452	137748837	-1.3796	-4.1035	-4.3367	-4.3394	9.1000	7.7204	4.9966	4.7634	4.7606	4.0706
1417464_at	Tnnc2	2	164602661	164605467	-1.7161	-3.9914	-4.4987	-4.5239	10.7374	9.0212	6.7460	6.2387	6.2135	4.0462
1427053_at	Abi3bp	16	56478013	56689430	2.9761	4.1047	4.8988	4.5344	5.7673	8.7434	9.8719	10.6660	10.3017	3.9306
1450430_at	Mrc1	2	14151041	14253684	1.0620	2.6124	3.9640	4.6726	7.0555	8.1175	9.6679	11.0195	11.7281	3.7967
1436717_x_at	Hbb-y	7	111000259	111001754	-4.2661	-4.4007	-4.3692	-4.3412	9.0326	4.7665	4.6319	4.6634	4.6914	3.7771
1418370_at	Tnnc1	14	32021498	32024909	-1.6058	-3.6074	-4.3154	-4.3442	11.7824	10.1766	8.1750	7.4671	7.4382	3.6489
1419728_at	Cxcl5	5	91188325	91190651	2.1103	3.0448	4.7244	4.3608	5.7145	7.8247	8.7593	10.4389	10.0753	3.6259
1436823_x_at	Hbb-y	7	111000259	111001754	-4.2136	-4.2917	-4.2540	-4.2597	9.1159	4.9023	4.8242	4.8619	4.8562	3.6214
1427054_s_at	Abi3bp	16	56478013	56689430	2.6954	4.0028	4.5389	4.4136	7.2964	9.9918	11.2993	11.8353	11.7100	3.5951
1419527_at	Comp	8	72897457	72905964	2.8216	4.2151	4.3873	4.3562	8.2426	11.0642	12.4576	12.6299	12.5988	3.5376
1425951_a_at	Clec4n	6	123179861	123197039	0.6770	1.3681	3.3746	4.4428	5.4887	6.1657	6.8568	8.8633	9.9315	3.5006
1417023_a_at	Fabp4	3	10204347	10208554	2.9987	3.9345	4.0636	4.6498	5.3050	8.3037	9.2395	9.3686	9.9548	3.4107
1419606_a_at	Tnnt1	7	4456265	4467577	-2.4081	-4.1099	-4.1154	-4.1149	11.5578	9.1497	7.4479	7.4424	7.4429	3.2641
1452651_a_at	Myl1	1	66970869	66991978	-1.3224	-3.3107	-3.8317	-4.1346	10.1862	8.8638	6.8755	6.3546	6.0517	3.1842
1448617_at	Cd53	3	106562851	106592969	0.6529	1.8630	3.5141	4.1134	6.8677	7.5207	8.7308	10.3819	10.9811	3.1471
1426278_at	Ifi27l2a	12	104680377	104681890	0.4809	1.3889	3.2256	4.0734	6.6772	7.1581	8.0661	9.9028	10.7506	3.0861
1448591_at	Ctss	3	95330708	95360322	0.4309	1.9994	3.2421	3.9931	8.4830	8.9140	10.4824	11.7252	12.4762	2.9888

					log2(fold-change from day 3)				Mean expression values (RMA)					
probe_id	gene symbol	chr	start	end	day 6	day 9	day 12	day 15	day 3	day 6	day 9	day 12	day 15	variance
1419391_at	Myog	1	136186590	136189127	-1.9464	-3.6449	-3.9306	-3.9524	10.3533	8.4069	6.7084	6.4227	6.4009	2.9584
1438855_x_at	Tnfaip2	12	112680680	112693229	1.5297	2.9810	4.0019	3.9372	6.2997	7.8294	9.2807	10.3016	10.2369	2.9359
1427735_a_at	Acta1	8	126415667	126418636	-1.4899	-3.2470	-3.8373	-3.8885	12.3280	10.8381	9.0810	8.4907	8.4395	2.8861
1422580_at	Myl4	11	104411977	104457067	-1.2078	-3.3364	-3.6169	-3.7973	10.9245	9.7167	7.5881	7.3076	7.1273	2.8728
1451941_a_at	Fcgr2b	1	172890689	172906678	0.7243	2.1133	3.4619	3.9098	7.3098	8.0341	9.4231	10.7717	11.2196	2.8539
1449388_at	Thbs4	13	93521546	93564713	1.3036	2.7991	3.6486	3.9751	8.8137	10.1173	11.6128	12.4623	12.7888	2.7866
1450792_at	Tyrobp	7	31198809	31202596	1.1380	2.0053	3.3228	4.1472	7.7977	8.9356	9.8029	11.1204	11.9448	2.7570
1449164_at	Cd68	11	69477715	69479655	1.4224	2.4527	3.3931	4.2322	6.9833	8.4057	9.4361	10.3764	11.2155	2.7529
1420804_s_at	Clec4d	6	123212135	123225283	0.7201	1.8628	3.1440	3.9967	6.6499	7.3700	8.5127	9.7939	10.6466	2.7344
1422124_a_at	Ptprc	1	139959992	140071843	0.7211	1.7985	3.2187	3.9480	6.4277	7.1488	8.2262	9.6464	10.3757	2.7341
1451263_a_at	Fabp4	3	10204347	10208554	2.6967	3.5650	3.6566	4.1134	5.2918	7.9885	8.8568	8.9485	9.4052	2.7236
1448620_at	Fcgr3	1	172981301	172989493	0.2427	1.7134	2.8597	3.8196	7.1245	7.3671	8.8379	9.9842	10.9441	2.7121
1455893_at	Rspo2	15	42852357	43002364	2.1161	3.3972	3.9376	3.7429	8.2240	10.3401	11.6212	12.1616	11.9670	2.6794
1425039_at	Itgbl1	14	124059193	124374840	2.7820	3.7182	3.7379	3.7443	7.1399	9.9219	10.8581	10.8778	10.8842	2.6137
1449519_at	Gadd45a	6	66985090	66987451	-0.1253	1.2329	3.4583	2.7300	6.7685	6.6432	8.0015	10.2269	9.4985	2.5757
1421114_a_at	Epyc	10	97106702	97145088	3.3304	3.8301	3.6631	3.4034	7.9513	11.2817	11.7813	11.6144	11.3546	2.5703
1449824_at	Prg4	1	152296946	152313295	3.2054	3.9270	3.7802	2.9306	6.8810	10.0864	10.8080	10.6613	9.8117	2.5619
1419905_s_at	Hpgd	8	58773382	58799002	0.5774	2.5062	3.0724	3.6762	6.7598	7.3372	9.2660	9.8322	10.4360	2.5585
1415983_at	Lcp1	14	75530908	75630649	1.2038	2.1564	3.3374	3.9085	6.9076	8.1114	9.0641	10.2450	10.8161	2.5039
1426260_a_at	ambiguous	1	ambiguous	ambiguous	0.6613	1.9106	2.9918	3.7608	7.8497	8.5110	9.7604	10.8416	11.6105	2.4482
1421551_s_at	Ifi202b	1	175892706	175912872	1.0727	2.2498	3.3714	3.6972	6.3528	7.4255	8.6026	9.7243	10.0500	2.4133
1435191_at	Cdsn	17	35693152	35694131	0.1683	0.4562	3.0250	3.0301	8.6863	8.8546	9.1424	11.7112	11.7163	2.4112
1436790_a_at	Sox11	12	28019129	28027439	-1.5444	-2.7222	-3.3887	-3.8063	9.5920	8.0476	6.8698	6.2033	5.7856	2.3733
1426005_at	Dmp1	5	104631636	104643121	2.0530	3.3230	3.8341	3.2646	5.2567	7.3096	8.5797	9.0908	8.5213	2.3729
1450813_a_at	Tnni1	1	137679642	137707566	-1.6209	-3.2994	-3.5227	-3.4269	11.3721	9.7512	8.0728	7.8494	7.9453	2.3718
1431056_a_at	Lpl	8	71404454	71430831	0.7191	2.0978	3.1896	3.5346	6.5439	7.2630	8.6417	9.7335	10.0785	2.3446
1451932_a_at	Adamtsl4	3	95480124	95491840	2.9637	3.5144	3.4966	3.5298	6.4237	9.3874	9.9381	9.9203	9.9535	2.3365
1448061_at	Msr1	8	40688806	40728032	0.7190	1.9921	3.1039	3.5882	7.5247	8.2437	9.5168	10.6287	11.1130	2.3277
1436905_x_at	Laptm5	4	130469040	130492056	0.6813	1.8650	2.9319	3.6681	7.1215	7.8028	8.9865	10.0534	10.7896	2.3156
1419589_at	Cd93	2	148262376	148269299	-0.1192	1.2927	2.3677	3.3860	6.0595	5.9403	7.3522	8.4272	9.4455	2.2897
1421001_a_at	Car6	4	149561124	149575441	0.1188	0.5227	3.2726	2.5197	7.1411	7.2599	7.6638	10.4137	9.6608	2.2669
1415904_at	Lpl	8	71404454	71430831	1.5461	2.5607	3.4967	3.5775	8.9276	10.4737	11.4883	12.4243	12.5051	2.2425

					log2(fold-change from day 3)				Mean expression values (RMA)					
probe_id	gene symbol	chr	start	end	day 6	day 9	day 12	day 15	day 3	day 6	day 9	day 12	day 15	variance
1437726_x_at	C1qb	4	136436044	136442102	0.2930	0.9970	2.2446	3.6042	5.4493	5.7423	6.4462	7.6939	9.0534	2.2289
1417063_at	C1qb	4	136436044	136442102	0.1879	1.2208	2.1648	3.6043	7.7556	7.9435	8.9765	9.9204	11.3599	2.2247
1452016_at	Alox5ap	5	150076634	150076758	0.3136	1.5070	2.6866	3.4077	8.3194	8.6331	9.8265	11.0060	11.7272	2.1676
1427076_at	Mpeg1	19	12535546	12538291	0.2456	1.4497	2.5704	3.4225	7.8259	8.0715	9.2756	10.3962	11.2484	2.1651
1416077_at	Adm	7	117771183	117773333	0.7699	1.7761	3.2005	3.3200	8.2530	9.0229	10.0292	11.4535	11.5730	2.1432
1456014_s_at	Fermt3	19	7073468	7093921	1.0194	2.0163	2.9989	3.6119	5.6912	6.7106	7.7075	8.6901	9.3031	2.1332
1421855_at	Fgl2	5	20878460	20884192	0.5598	2.0227	3.3851	2.8550	5.4142	5.9740	7.4370	8.7994	8.2692	2.1118
1450883_a_at	Cd36	5	17287834	17341514	0.1486	1.6936	2.5465	3.2644	6.1375	6.2861	7.8311	8.6840	9.4019	2.0793
1417388_at	Bex2	X	132601103	132602775	-2.6938	-3.1540	-3.3599	-3.4296	9.1125	6.4187	5.9586	5.7527	5.6830	2.0787
1433963_a_at	Fermt3	19	7073468	7093921	0.7957	1.6941	2.7388	3.5789	4.8214	5.6171	6.5155	7.5603	8.4003	2.0745
1448749_at	Plek	11	16871209	16952384	0.4842	1.6762	2.9349	3.2386	6.1360	6.6202	7.8122	9.0709	9.3745	2.0639
1417051_at	Pcdh8	14	80166592	80171107	-0.3308	-2.5044	-2.8517	-2.9307	8.2380	7.9072	5.7336	5.3863	5.3072	2.0626
1417426_at	Srgn	10	61956581	61990199	1.6662	2.3603	3.3849	3.4997	6.7137	8.3800	9.0740	10.0987	10.2134	2.0606
1448303_at	Gpnmb	6	48986612	49006778	0.7461	1.9210	2.7407	3.5182	8.5410	9.2871	10.4620	11.2817	12.0592	2.0503
1448748_at	Plek	11	16871209	16952384	0.3902	1.8223	2.9243	3.1169	6.6263	7.0165	8.4486	9.5506	9.7432	2.0287
1417009_at	C1ra	6	124462423	124473142	0.9981	2.2997	3.1697	3.2280	6.5607	7.5588	8.8604	9.7304	9.7887	1.9878
1419700_a_at	Prom1	5	44384861	44492920	-1.5091	-2.9148	-3.1093	-3.2666	10.0704	8.5613	7.1555	6.9610	6.8038	1.9462
1460248_at	Cpxm2	7	139234499	139346422	2.8309	3.2431	3.1708	3.1488	8.2353	11.0662	11.4784	11.4061	11.3841	1.9451
1451683_x_at	H2-D1	17	35400058	35404127	1.0842	1.8543	2.9744	3.4155	7.2538	8.3380	9.1081	10.2283	10.6693	1.9307
1416053_at	Lrrn1	6	107479777	107520204	-2.1237	-2.8567	-3.3799	-3.2611	8.2670	6.1433	5.4102	4.8870	5.0058	1.9295
1423413_at	Ndrg1	15	66760880	66801203	2.3541	2.8124	3.2824	3.4023	6.1999	8.5540	9.0123	9.4822	9.6021	1.9277
1420464_s_at	ambiguous	7	ambiguous	ambiguous	0.2161	1.3037	2.2981	3.2494	5.5484	5.7645	6.8521	7.8465	8.7978	1.8992
1418340_at	Fcer1g	1	173159703	173164496	0.7414	1.6564	2.5960	3.4269	7.8209	8.5623	9.4773	10.4169	11.2478	1.8986
1417633_at	Sod3	5	52755228	52760744	1.9557	2.6242	3.2345	3.3965	7.1496	9.1053	9.7738	10.3841	10.5461	1.8931
1417381_at	C1qa	4	136451832	136454718	0.2712	1.3148	2.0527	3.3710	8.4389	8.7102	9.7537	10.4916	11.8099	1.8880
1419873_s_at	Csf1r	18	61265226	61290788	0.7604	1.4203	2.4233	3.4770	8.5471	9.3074	9.9674	10.9704	12.0241	1.8743
1453321_at	Fndc1	17	7931433	7997839	1.9046	2.7655	3.2703	3.2202	8.2312	10.1358	10.9967	11.5015	11.4514	1.8571
1449368_at	Dcn	10	96945001	96980785	1.9271	3.0601	3.1989	3.0150	9.3634	11.2905	12.4235	12.5623	12.3783	1.8270
1450241_a_at	Evi2a	11	79340062	79344111	1.1165	2.1770	2.7721	3.3758	6.3626	7.4791	8.5396	9.1347	9.7384	1.8096
1452141_a_at	Sepp1	15	3218547	3230507	0.2735	1.7686	2.5633	3.0106	10.2166	10.4900	11.9852	12.7799	13.2272	1.8091
1426225_at	Rbp4	19	38191119	38199738	1.5084	2.4958	3.0031	3.3284	8.8056	10.3139	11.3014	11.8086	12.1340	1.8090
1449556_at	H2-T23	17	36166921	36169646	1.2198	2.0611	2.9503	3.3143	6.4704	7.6903	8.5315	9.4207	9.7847	1.8004

					log2(fold-change from day 3)				Mean expression values (RMA)					
probe_id	gene symbol	chr	start	end	day 6	day 9	day 12	day 15	day 3	day 6	day 9	day 12	day 15	variance
1419599_s_at	Ms4a6d	19	11661094	11679339	0.4200	1.3379	2.2966	3.2664	6.0064	6.4264	7.3442	8.3030	9.2727	1.7977
1426906_at	Ifi205	1	175942129	175961886	0.2315	0.6064	2.5691	2.8100	3.9063	4.1378	4.5128	6.4755	6.7163	1.7969
1419872_at	Csf1r	18	61265226	61290788	0.7311	1.4349	2.5526	3.3112	9.4700	10.2011	10.9049	12.0226	12.7811	1.7944
1452231_x_at	Ifi205	1	175942129	175961886	0.0658	0.6571	2.3455	2.8703	4.7073	4.7732	5.3644	7.0528	7.5776	1.7806
1424443_at	Tm6sf1	7	89003887	89029320	-0.6403	0.1762	1.8409	2.4955	6.4094	5.7691	6.5855	8.2503	8.9048	1.7646
1456174_x_at	Ndrg1	15	66760880	66801203	2.2135	2.8682	3.1770	3.1028	7.3212	9.5348	10.1895	10.4982	10.4240	1.7575
1448260_at	Uchl1	5	67067529	67078473	-1.0622	-2.0290	-2.9802	-3.1410	10.5428	9.4806	8.5138	7.5626	7.4018	1.7547
1425894_at	Mrgprf	7	152486733	152495462	2.6822	3.2952	2.9179	2.7579	7.7906	10.4728	11.0859	10.7085	10.5485	1.7534
1416034_at	Cd24a	10	43298977	43304066	-1.2381	-2.1995	-3.1389	-3.0582	12.1817	10.9436	9.9822	9.0427	9.1235	1.7527
1451567_a_at	Ifi203	1	175850538	175872803	0.2202	0.8758	2.2713	3.0264	4.5612	4.7813	5.4370	6.8324	7.5876	1.7394
1449401_at	C1qc	4	136445719	136448980	0.2940	1.1722	1.9055	3.2669	7.6560	7.9501	8.8283	9.5615	10.9230	1.7375
1455332_x_at	Fcgr2b	1	172890689	172906678	0.5712	1.5106	2.6024	3.0948	7.5344	8.1056	9.0450	10.1368	10.6293	1.7140
1419627_s_at	Clec4n	6	123179861	123197039	0.2104	0.4588	1.9783	3.0142	8.2621	8.4725	8.7209	10.2405	11.2763	1.7107
1426261_s_at	ambiguous	1	ambiguous	ambiguous	0.3142	1.2072	2.2517	3.1055	6.4926	6.8068	7.6998	8.7443	9.5981	1.7018
1451931_x_at	H2-D1	17	35400039	35404440	1.1851	1.7699	2.8442	3.2447	9.1515	10.3366	10.9215	11.9957	12.3962	1.6990
1437502_x_at	Cd24a	10	43298977	43304066	-1.1893	-1.9436	-2.7968	-3.2683	10.7510	9.5617	8.8075	7.9542	7.4827	1.6938
1454086_a_at	Lmo2	2	103798143	103822031	0.3039	1.1080	2.2399	3.0795	7.8236	8.1275	8.9316	10.0635	10.9031	1.6896
1421404_at	Cxcl15	5	91223560	91232093	0.6789	2.2083	2.8861	2.7648	6.8513	7.5303	9.0596	9.7374	9.6161	1.6828
1417936_at	Ccl9	11	83386421	83392138	1.3693	1.9531	2.6872	3.3873	7.4057	8.7750	9.3589	10.0929	10.7930	1.6810
1448995_at	Pf4	5	91201539	91202409	0.6910	1.7284	2.2979	3.2807	8.0648	8.7559	9.7933	10.3628	11.3456	1.6787
1448182_a_at	Cd24a	10	43298977	43304066	-1.2147	-2.1041	-2.9258	-3.1242	11.9082	10.6934	9.8040	8.9824	8.7840	1.6672
1419598_at	Ms4a6d	19	11661094	11679339	0.3448	1.1361	2.1660	3.1139	6.4363	6.7812	7.5724	8.6023	9.5503	1.6640
1422637_at	Rassf5	1	133072987	133141835	0.5757	1.4159	2.8267	2.8333	5.8654	6.4411	7.2813	8.6921	8.6986	1.6612
1424754_at	Ms4a7	19	11395529	11410636	0.3831	1.1193	1.7703	3.2634	7.9453	8.3284	9.0646	9.7156	11.2087	1.6598
1418809_at	Pira1	7	3683235	3691472	-0.0403	1.4053	1.9723	2.9006	5.8073	5.7670	7.2125	7.7795	8.7079	1.6244
1451784_x_at	H2-D1	17	35400039	35404440	1.0022	1.7477	2.7500	3.0996	9.8503	10.8525	11.5980	12.6003	12.9499	1.6096
1424542_at	S100a4	3	90407693	90409967	2.7830	3.0937	2.4996	2.8052	9.4679	12.2509	12.5616	11.9675	12.2731	1.6070
1450988_at	Lgr5	10	114887367	115024836	0.9484	2.0000	2.7440	3.0270	8.1935	9.1419	10.1935	10.9375	11.2205	1.5965
1448327_at	Actn2	13	12361693	12433027	-1.5772	-2.8138	-2.8390	-2.9197	9.9838	8.4067	7.1701	7.1448	7.0642	1.5965
1417933_at	Igfbp6	15	101974793	101979942	1.0509	2.1198	2.7423	3.0498	7.8875	8.9384	10.0073	10.6298	10.9372	1.5883
1425545_x_at	H2-Q2	17	35400039	35404442	1.0596	1.7126	2.7427	3.0828	10.0160	11.0755	11.7285	12.7587	13.0988	1.5745
1426808_at	Lgals3	14	47987426	48005835	1.7108	2.4203	2.9339	3.0636	9.8009	11.5117	12.2212	12.7348	12.8645	1.5651

					log2(fold-change from day 3)				Mean expression values (RMA)					
probe_id	gene symbol	chr	start	end	day 6	day 9	day 12	day 15	day 3	day 6	day 9	day 12	day 15	variance
1423135_at	Thy1	9	43851467	43856662	1.7023	2.6136	2.8952	2.9731	8.7319	10.4342	11.3454	11.6271	11.7050	1.5516
1424041_s_at	C1s	6	124480363	124492299	0.4406	1.2980	2.6242	2.7287	6.6426	7.0833	7.9407	9.2668	9.3713	1.5383
1456225_x_at	Trib3	2	152163158	152169768	0.0434	1.2174	2.8346	2.0403	8.0195	8.0629	9.2368	10.8540	10.0597	1.5381
1450678_at	Itgb2	10	76992997	77028453	0.5246	1.0894	2.1945	3.0198	7.1205	7.6452	8.2099	9.3151	10.1403	1.5180
1448160_at	Lcp1	14	75530908	75630649	0.7629	1.3177	2.5238	2.9828	7.7458	8.5087	9.0635	10.2696	10.7286	1.5180
1415931_at	Igf2	7	149836673	149852721	-0.0467	-0.4588	-1.5083	-2.8967	10.7196	10.6729	10.2608	9.2113	7.8229	1.5140
1417634_at	Sod3	5	52755228	52760744	1.5135	2.2729	2.7788	3.0739	6.7168	8.2303	8.9897	9.4956	9.7907	1.5112
1417356_at	Peg3	7	6656603	6683132	-0.3044	-0.8064	-2.2120	-2.8176	11.1018	10.7973	10.2954	8.8897	8.2841	1.5085
1418796_at	Clec11a	7	51559642	51562256	2.7553	3.0568	2.5463	2.4023	8.7213	11.4765	11.7780	11.2675	11.1236	1.5079
1426065_a_at	Trib3	2	152163158	152169768	0.0389	1.2187	2.7248	2.1484	8.1304	8.1693	9.3490	10.8552	10.2788	1.5024
1451161_a_at	Emr1	17	57498114	57622950	0.1136	0.8541	1.9795	2.8207	8.1676	8.2812	9.0217	10.1471	10.9883	1.4909
1423555_a_at	Ifi44	3	151393886	151412911	0.1744	0.8945	2.6284	2.3516	6.2049	6.3793	7.0994	8.8333	8.5565	1.4878
1427445_a_at	Ttn	2	76542037	76820604	-1.8925	-2.6668	-2.8605	-2.8655	8.8546	6.9621	6.1878	5.9941	5.9891	1.4824
1449340_at	Sostdc1	12	37040823	37044311	-0.0157	-0.6852	-1.9050	-2.7441	9.3378	9.3221	8.6526	7.4328	6.5936	1.4761
1417355_at	Peg3	7	6656603	6683132	-0.3648	-0.8342	-1.9077	-2.9600	12.7220	12.3573	11.8878	10.8143	9.7620	1.4673
1426766_at	6330403K07Rik	11	70845451	70847015	-1.1680	-1.5252	-2.6469	-3.0232	10.8787	9.7106	9.3535	8.2318	7.8555	1.4618
1433924_at	Peg3	7	6656603	6683132	-0.3347	-0.7144	-1.9229	-2.8999	12.4878	12.1532	11.7735	10.5649	9.5879	1.4584
1417268_at	Cd14	18	36884721	36886308	0.3907	1.6521	2.2274	2.8508	8.1271	8.5178	9.7792	10.3546	10.9780	1.4572
1422573_at	Ampd3	7	117911737	117955919	0.6630	1.6751	2.4620	2.8840	6.6925	7.3555	8.3676	9.1545	9.5765	1.4538
1425214_at	P2ry6	7	108086148	108112876	0.1182	0.9522	2.1771	2.6761	7.5834	7.7016	8.5357	9.7605	10.2595	1.4511
1418933_at	Slc1a6	10	78243247	78277510	0.4052	1.3686	2.3317	2.7984	7.0612	7.4664	8.4298	9.3929	9.8596	1.4430
1433474_at	Edil3	13	88961077	89462828	1.7866	2.6944	2.8303	2.7219	7.2611	9.0477	9.9555	10.0914	9.9830	1.4345
1448550_at	Lbp	2	158132229	158158588	1.7660	2.5848	2.9058	2.7207	8.7546	10.5206	11.3394	11.6605	11.4753	1.4342
1416246_a_at	Coro1a	7	133843287	133848330	0.0895	1.2538	2.2017	2.6345	7.7792	7.8687	9.0329	9.9809	10.4137	1.4327
1418420_at	Myod1	7	53631852	53634465	-1.7544	-2.6692	-2.6723	-2.8437	9.6673	7.9128	6.9981	6.9950	6.8235	1.4178
1424683_at	Fam134b	15	25773019	25903442	1.5952	2.2882	2.9184	2.7531	7.3089	8.9041	9.5971	10.2273	10.0620	1.4045
1433919_at	Asb4	6	5333386	5383022	-1.2612	-2.3596	-2.6639	-2.8011	8.4463	7.1851	6.0868	5.7824	5.6453	1.3976
1419209_at	Cxcl1	5	91320267	91322141	0.7029	0.6661	2.7291	2.3285	6.3197	7.0226	6.9858	9.0488	8.6482	1.3869
1448929_at	F13a1	13	36959056	37141073	1.1068	2.4484	2.2283	2.8310	6.5224	7.6292	8.9708	8.7507	9.3534	1.3394
1427789_s_at	Gnas	2	174109821	174172245	1.3891	0.9752	-0.6803	-1.4129	8.6558	10.0448	9.6310	7.9755	7.2429	1.3312
1448380_at	Lgals3bp	11	118254065	118263406	0.7867	1.8883	2.5906	2.6196	8.6079	9.3945	10.4961	11.1985	11.2275	1.3307
1451755_a_at	Apobec1	6	122527810	122552462	0.3552	1.1164	2.2272	2.6589	8.2039	8.5590	9.3203	10.4310	10.8628	1.3297

					log2(fold-change from day 3)				Mean expression values (RMA)					
probe_id	gene symbol	chr	start	end	day 6	day 9	day 12	day 15	day 3	day 6	day 9	day 12	day 15	variance
1418945_at	Mmp3	9	7445822	7455972	0.3690	1.1181	2.4513	2.4786	6.3560	6.7250	7.4742	8.8073	8.8346	1.3258
1417741_at	Pygl	12	71291798	71332475	1.8192	2.7071	2.4485	2.7599	6.3835	8.2027	9.0907	8.8320	9.1435	1.3243
1419482_at	C3ar1	6	122797156	122806179	0.3841	1.2690	2.0607	2.7529	7.9959	8.3800	9.2649	10.0566	10.7488	1.3048
1415832_at	Agtr2	X	21061670	21066290	1.8691	2.0254	0.8123	-0.5799	8.3915	10.2606	10.4169	9.2038	7.8115	1.2964
1416985_at	Sirpa	2	129418571	129457964	0.7414	1.4202	2.1223	2.8882	8.4517	9.1931	9.8719	10.5739	11.3399	1.2811
1420760_s_at	Ndrg1	15	66760880	66801203	1.8805	2.3518	2.6669	2.7424	8.6277	10.5082	10.9795	11.2947	11.3702	1.2771
1423593_a_at	Csf1r	18	61265226	61290788	0.7662	1.1940	1.9332	2.9473	8.2913	9.0575	9.4853	10.2245	11.2386	1.2694
1418203_at	Pmaip1	18	66618258	66625212	-0.8079	-2.1150	-2.3205	-2.6306	10.2849	9.4770	8.1699	7.9645	7.6544	1.2576
1416871_at	Adam8	7	147164831	147178461	0.5736	1.6600	2.2980	2.6303	7.6512	8.2249	9.3112	9.9492	10.2815	1.2563
1415997_at	Txnip	3	96361880	96365806	1.0483	1.9633	2.5873	2.6288	10.3356	11.3839	12.2989	12.9229	12.9643	1.2548
1448377_at	Slpi	2	164179806	164182243	1.2417	1.9522	2.4498	2.8323	9.5863	10.8280	11.5385	12.0361	12.4187	1.2520
1421792_s_at	Trem2	17	48485726	48493472	0.2258	0.9220	1.9142	2.6313	7.7879	8.0137	8.7099	9.7021	10.4192	1.2516
1450976_at	Ndrg1	15	66760880	66801203	1.8723	2.2839	2.6750	2.7032	8.2004	10.0726	10.4843	10.8754	10.9036	1.2509
1448898_at	Ccl9	11	83386421	83392138	1.0145	1.4411	2.2531	2.8877	6.4450	7.4595	7.8861	8.6980	9.3326	1.2450
1452244_at	6330406I15Rik	5	150171051	150234298	1.6238	2.0810	2.6752	2.7254	6.7810	8.4047	8.8619	9.4562	9.5063	1.2425
1418745_at	Omd	13	49678118	49687978	0.7054	2.2997	2.2601	2.4323	6.9269	7.6323	9.2267	9.1870	9.3592	1.2400
1427919_at	Srpx2	X	130442965	130466985	1.7403	2.5487	2.5822	2.5590	7.8253	9.5655	10.3740	10.4075	10.3842	1.2388
1422046_at	Itgam	7	135206154	135271674	0.1300	0.8870	2.0591	2.4410	5.8753	6.0053	6.7623	7.9344	8.3162	1.2286
1422317_a_at	Il1rl1	1	40496621	40522241	1.2138	2.1699	2.5471	2.5950	7.2707	8.4845	9.4406	9.8178	9.8657	1.2164
1419015_at	Wisp2	2	163646597	163658882	1.3655	2.1057	2.6202	2.6264	9.4349	10.8004	11.5406	12.0551	12.0613	1.2155
1417492_at	Ctsb	14	63741353	63761971	0.6481	1.3584	2.0413	2.7654	7.8106	8.4587	9.1689	9.8519	10.5760	1.1989
1424354_at	Tmem140	6	34813146	34824946	0.5055	1.5117	2.0834	2.6310	6.1300	6.6356	7.6417	8.2134	8.7611	1.1851
1423166_at	Cd36	5	17287834	17341514	0.0214	1.1671	1.7000	2.4993	6.0240	6.0454	7.1911	7.7240	8.5233	1.1734
1455269_a_at	Coro1a	7	133843287	133848330	-0.0364	0.9564	1.8363	2.3742	6.5481	6.5117	7.5044	8.3844	8.9223	1.1651
1428922_at	1200009O22Rik	6	53765460	53770824	-1.6909	-1.7142	-2.3788	-2.8366	10.4854	8.7945	8.7712	8.1066	7.6488	1.1600
1424680_at	Fam26e	10	33811158	33816325	1.0350	1.8641	2.5423	2.4947	7.2461	8.2811	9.1103	9.7884	9.7409	1.1592
1425622_at	Edil3	13	88961077	89462828	1.6190	2.2074	2.5283	2.5924	7.2759	8.8949	9.4833	9.8041	9.8683	1.1491
1415996_at	Txnip	3	96361880	96365806	0.9760	1.8973	2.4142	2.5426	10.3755	11.3515	12.2728	12.7897	12.9181	1.1459
1433678_at	Pld4	12	113998866	114007197	0.2280	0.9032	1.8058	2.5343	7.1007	7.3287	8.0039	8.9065	9.6350	1.1411
1448754_at	Rbp1	9	98325236	98346967	-1.1153	-1.6188	-2.2979	-2.7362	11.0806	9.9653	9.4618	8.7827	8.3444	1.1406
1417516_at	Ddit3	10	126727830	126733342	0.1845	1.0862	2.4739	1.8811	8.8738	9.0582	9.9599	11.3476	10.7548	1.1357
1417481_at	Ramp1	1	93076623	93120973	0.4148	1.8350	2.1553	2.3235	7.8723	8.2872	9.7074	10.0276	10.1959	1.1321

					log2(fold-change from day 3)				Mean expression values (RMA)					
probe_id	gene symbol	chr	start	end	day 6	day 9	day 12	day 15	day 3	day 6	day 9	day 12	day 15	variance
1418511_at	Dpt	1	166726863	166754397	2.2206	2.6672	2.3575	2.0215	7.7617	9.9823	10.4289	10.1192	9.7832	1.1286
1450199_a_at	Stab1	14	31952199	31981827	0.3878	0.8977	1.6222	2.6745	7.0516	7.4394	7.9493	8.6738	9.7261	1.1271
1460693_a_at	Col9a3	2	180332495	180356890	0.5150	0.3117	-0.7275	-2.0983	8.7034	9.2184	9.0151	7.9759	6.6050	1.1238
1451174_at	Lrrc33	16	32142871	32165680	0.2043	0.8340	1.8302	2.4690	7.1197	7.3240	7.9537	8.9498	9.5887	1.1213
1434366_x_at	C1qb	4	136436047	136436439	0.1290	0.6386	0.9944	2.6286	8.2101	8.3391	8.8487	9.2045	10.8386	1.1168
1419442_at	Matn2	15	34236436	34365997	1.9299	2.3850	2.5404	2.3512	6.4715	8.4014	8.8565	9.0119	8.8227	1.1107
1451190_a_at	Sbk1	7	133416133	133438505	-1.2544	-1.9142	-2.4354	-2.5857	10.3652	9.1108	8.4510	7.9298	7.7795	1.1101
1418240_at	Gbp2	3	142283627	142300972	0.7210	1.3881	2.2688	2.5246	6.1325	6.8534	7.5205	8.4013	8.6571	1.1097
