# Supplementary material for: Systems Genetics Analysis of Mouse Chondrocyte Differentiation
Source: J Bone Miner Res. 2010 Oct 14;26(4):747–60. doi: 10.1002/jbmr.271 (PMC3179327; doi:10.1002/jbmr.271)
Supplement: Supplementary file 8 [file jbmr0026-0747-SD8.pdf]

Probe set	Gene	Module in WGCNA	Kin in WGCNA
scl25652.4_232-S	Tmem64	salmon	17.03693371
scl40222.12.1_44-S	Slc22a4	salmon	16.40012276
scl012575.1_2-S	Cdkn1a	salmon	16.39528538
scl0228608.7_126-S	Smox	salmon	15.85678545
scl00209630.2_217-S	Frmd4a	salmon	14.89609383
scl00100169.1_330-S	Phactr4	salmon	14.85181292
scl015528.3_20-S	Hspe1	salmon	14.69251978
scl25869.12_138-S	Hrbl	salmon	14.47026869
scl0015516.2_144-S	Hspcb	salmon	14.31607453
scl0017936.1_141-S	Nab1	salmon	14.1615444
scl013046.9_64-S	Cugbp1	salmon	13.91585672
scl000928.1_86-S	Hspd1	salmon	13.68627175
scl36963.5_95-S	Pou2af1	salmon	13.57138861
scl54620.5_512-S	Bhlhb9	salmon	13.18117907
scl015519.1_32-S	Hspca	salmon	13.04863819
scl54754.6.1_12-S	Igbp1	salmon	12.74380494
gi_7305154_ref_NM_013556.1__205-S	Hprt	salmon	12.62001376
scl52490.15_282-S	Smbp	salmon	12.51335773
scl066989.6_85-S	2410004N1	salmon	12.49581422
scl015516.6_124-S	Hspcb	salmon	12.09307584
scl22141.1.362_235-S	Wwtr1	salmon	11.89727524
scl0003360.1_0-S	Smox	salmon	11.75918015
scl0001816.1_0-S	Eif4a2	salmon	11.50012715
scl44868.8_25-S	Gcnt2	salmon	11.42565679
scl54358.13_95-S	Syn1	salmon	10.97342916
scl52902.14.1_38-S	Stip1	salmon	10.58241196
scl0015516.2_121-S	Hspcb	salmon	10.35842317
scl44387.13_320-S	Plk2	salmon	10.19910771
scl19176.21_114-S	Stk39	salmon	10.1012166
scl37865.9_4-S	D10Ucla1	salmon	10.05775173
scl37195.16.1_126-S	Bmper	salmon	9.95871652
scl0170750.1_173-S	Xpnpep1	salmon	9.264498558
scl24120.2_589-S	Cdkn2b	salmon	8.974098456
scl39328.7_317-S	Grb2	salmon	8.84565466
ri|D130063H01|PX00185M14|AK051669|2725-S	D130063H(	salmon	8.591490436
scl0020964.1_0-S	Syn1	salmon	8.343166749
scl0003512.1_8-S	Hspa8	salmon	8.150190131
scl019935.5_129-S	Mrpl23	salmon	7.989049449
ri|A830081L15|PX00155H24|AK044025|2877-S	A830081L1	salmon	6.076359003
ri|D030019N20|PX00179B01|AK050786|1930-S	D030019N2	salmon	5.881308863
GI_38080488-S	LOC27001	salmon	4.094786825

Module in MMC Pearson	Correlation of Genes in the Module of MMC Pearson
42	0.35155
42	0.35155
42	0.35155
42	0.35155
42	0.35155
42	0.35155
42	0.35155
42	0.35155
67	0
42	0.35155
42	0.35155
42	0.35155
42	0.35155
44	0.23013
42	0.35155
42	0.35155
42	0.35155
42	0.35155
42	0.35155
75	0
42	0.35155
42	0.35155
41	0.48805
42	0.35155
42	0.35155
42	0.35155
42	0.35155
44	0.23013
42	0.35155
42	0.35155
42	0.35155
44	0.23013
44	0.23013
44	0.23013
44	0.23013
42	0.35155
42	0.35155
42	0.35155
25	0.94832
25	0.94832
33	0.92433

Module in MMC Spearman	Correlation of Genes in the Module of MMC Spearman
144	0.46046
144	0.46046
144	0.46046
129	0.52685
140	0.48535
143	0.46582
129	0.52685
140	0.48535
131	0.51675
131	0.51675
131	0.51675
131	0.51675
144	0.46046
140	0.48535
93	0.62294
140	0.48535
144	0.46046
140	0.48535
141	0.4817
131	0.51675
102	0.60416
129	0.52685
131	0.51675
90	0.6297
23	0.82751
138	0.48828
131	0.51675
140	0.48535
114	0.56915
114	0.56915
107	0.59577
131	0.51675
139	0.48602
89	0.63024
131	0.51675
23	0.88343
93	0.62294
144	0.46046
97	0.61539
97	0.61539
97	0.61539
