# Supplementary material for: Systems Genetics Analysis of Mouse Chondrocyte Differentiation
Source: J Bone Miner Res. 2010 Oct 14;26(4):747–60. doi: 10.1002/jbmr.271 (PMC3179327; doi:10.1002/jbmr.271)
Supplement: Supplementary file 9 [file jbmr0026-0747-SD9.docx]

Figure S1: Base-2 logarithms of fold-changes relative to day 3 are plotted against days 3, 6, 9, 12 and 15 for the 200 most-varying probes.

Figure S2: Base-2 logarithms of fold-changes relative to day 3 are plotted against days 3, 6, 9, 12 and 15 for the 100 most-varying probes.

Figure S3: Modulated Modularity Clustering (MMC) with Pearson correlation

Figure S4: Modulated Modularity Clustering (MMC) with Spearman correlation
