# Supplementary material for: Systems Genetics Analysis of Mouse Chondrocyte Differentiation
Source: J Bone Miner Res. 2010 Oct 14;26(4):747–60. doi: 10.1002/jbmr.271 (PMC3179327; doi:10.1002/jbmr.271)
Supplement: Supplementary file 10 [file jbmr0026-0747-SD10.docx]

Supplementary Table 3

Previously published microarray expression data^(51)^ was downloaded from the Gene Expression Ombibus (GEO)(accession number GSE2154). RMA expression values were calculated using the *affy* R/Bioconductor package. The mean expression value of each probe was calculated using the three biological replicates for each time point, and the variance of those mean values was calculated across the five available time points. Fold-changes were all calculated relative to the first time point by taking the difference between each of the last four time points and the first time point and averaging the differences across the three biological replicates.

Supplementary Table 4

List of genes in salmon module from Weighted Gene Co-expression Network Analysis (WGCNA) and Modulated Modularity Clustering (MMC) method
